# Discovery of a small-molecule binder of the oncoprotein gankyrin that modulates gankyrin activity in the cell

**DOI:** 10.1038/srep23732

**Published:** 2016-04-05

**Authors:** Anasuya Chattopadhyay, Cornelius J. O’Connor, Fengzhi Zhang, Celine Galvagnion, Warren R. J. D. Galloway, Yaw Sing Tan, Jamie E. Stokes, Taufiq Rahman, Chandra Verma, David R. Spring, Laura S. Itzhaki

**Affiliations:** 1 Department of Pharmacology, Tennis Court Road, Cambridge CB2 1PD, UK; 2 Department of Chemistry, Lensfield Road, Cambridge CB2 1EW, UK; 3 Bioinformatics Institute (A*STAR), 30 Biopolis Street, #07-01 Matrix, Singapore 138671; 4 School of Biological Sciences, Nanyang Technological University, 60 Nanyang Drive, Singapore 637551; 5 Department of Biological Sciences, National University of Singapore, 14 Science Drive 4, Singapore 117543

## Abstract

Gankyrin is an ankyrin-repeat oncoprotein whose overexpression has been implicated in the development of many cancer types. Elevated gankyrin levels are linked to aberrant cellular events including enhanced degradation of tumour suppressor protein p53, and inhibition of gankyrin activity has therefore been identified as an attractive anticancer strategy. Gankyrin interacts with several partner proteins, and a number of these protein-protein interactions (PPIs) are of relevance to cancer. Thus, molecules that bind the PPI interface of gankyrin and interrupt these interactions are of considerable interest. Herein, we report the discovery of a small molecule termed cjoc42 that is capable of binding to gankyrin. Cell-based experiments demonstrate that cjoc42 can inhibit gankyrin activity in a dose-dependent manner: cjoc42 prevents the decrease in p53 protein levels normally associated with high amounts of gankyrin, and it restores p53-dependent transcription and sensitivity to DNA damage. The results represent the first evidence that gankyrin is a “druggable” target with small molecules.

Repeat proteins, such as ankyrin, leucine-rich, HEAT and tetratricopeptide repeats, are a distinctive class of proteins comprising tandem arrays of small structural motifs that stack in a linear manner to form elongated architectures. Gankyrin is an ankyrin-repeat oncoprotein^1^,^2^ with potent cell-cycle stimulatory and anti-apoptotic properties that is overexpressed early in hepatocellular carcinoma (HCC) and whose upregulation is associated with poor prognosis in esophageal squamous cell carcinoma (ESCC), glioblastomas, colorectal, pancreatic and lung carcinomas^3^,^4^,^5^. Existing treatment options for these cancers are limited, and no small-molecule inhibitors of gankyrin have been published to date; a broad-spectrum histone deacetylase inhibitor LBH589 is known to affect the proliferative and metastatic potential of gankyrin over-expressing HCCs, but it lacks selectivity^6^. Inhibitors of gankyrin are expected to have a negative effect on tumour formation and growth (indeed, inhibition of gankyrin expression by siRNA has been shown to have such effects both *in vitro* and *in vivo*^4^,^6^,^7^), and gankyrin is therefore regarded as a promising target for potential anticancer therapeutic agents^8^,^9^.

Gankyrin is composed of seven helix-turn-helix-loop ankyrin modules, which together generate a relatively featureless concave surface (Fig. 1a)^1^,^2^,^10^. This surface is presumed to be the interface through which gankyrin contacts several physiological binding partners. A number of these protein-protein interactions (PPIs) are known to be of relevance to cancer. For example, gankyrin enhances the phosphorylation of tumour suppressor protein pRb by binding to cyclin-dependent kinase 4 (CDK4) and abrogating CDK4 inhibition by tumour suppressor protein p16NK4a (referred to subsequently as p16). Gankyrin also binds to the ubiquitin ligase MDM2 and enhances the ubiquitination of p53^8^,^9^,^11^,^12^. Additionally, gankyrin is one of a number of repeat-protein “chaperones” required for the assembly of the proteasome regulatory particle. It binds the S6 ATPase subunit (also known as rpt3) of this particle^13^, and it is thought that through this interaction it further enhances the targeting of p53 (and possibly of pRb also) to the proteasome for degradation. Thus, gankyrin PPIs serve to modulate the destruction of principal tumour suppressor proteins p53 and pRb. Gankyrin overexpression results in decreased cellular levels of these two key proteins, leading ultimately to the onset of oncogenic cell functions and fate^11^,^14^,^15^. Modulation of disease-associated gankyrin activity will require physical disruption or inhibition of its PPIs, and this has been identified as an attractive therapeutic strategy for the treatment of numerous cancer types. Agents that are capable of binding the PPI surface of gankyrin would be of considerable value not only as potential starting points for the development of new cancer treatments but also as tools with which to further delineate the mechanisms of gankyrin function. Chapman and McNaughton have recently reported the synthesis of resurfaced proteins whose shapes are complementary to the putative PPI face of gankyrin; these proteins selectively bind to gankyrin^10^ and modulate its activity *in vitro*^14^. However, no selective, direct small-molecule binders of gankyrin capable of modulating gankyrin activity within a cancer cell are currently known. If such drug-like molecules could be identified they would ultimately have greater therapeutic potential than engineered proteins due to their small size and ease of cell penetration. Thakur and co-workers have reported computational modelling studies directed towards the design of a potent ligand to inhibit the activity of gankyrin, but these molecules were not experimentally validated^7^.

Herein, we report the discovery of a small molecule (designated “cjoc42”, Fig. 1b) that is capable of binding to (human) gankyrin. Our data suggest that cjoc42 targets the proposed PPI binding surface of gankyrin at the region where the protein interacts with the C-terminal portion of the S6 binding partner (referred to as S6C herein; the atomic structure of the complex between mouse gankyrin (mGankyrin) and S6C is known and shows that S6C binds to the concave face of gankyrin^13^; see Fig. 1a and Supplementary Fig. S1). In human osteosarcoma U2OS cells overexpressing gankyrin, low micromolar concentrations of cjoc42 prevented the decrease in p53 protein levels normally associated with high amounts of gankyrin, restored p53-dependent transcription and sensitivity to DNA damage. These results provide proof-of-principle that a small molecule targeting the PPI surface of gankyrin is able to modulate gankyrin activity in the cell. Small molecules of this kind should be useful as chemical probes with which to dissect further mechanistic details of gankyrin’s function. Of wider significance, these findings provide the first proof that gankyrin is a druggable target with small molecules.

## Results

### Screening small molecules for gankyrin binding using a thermal shift assay

**Figure 1:**
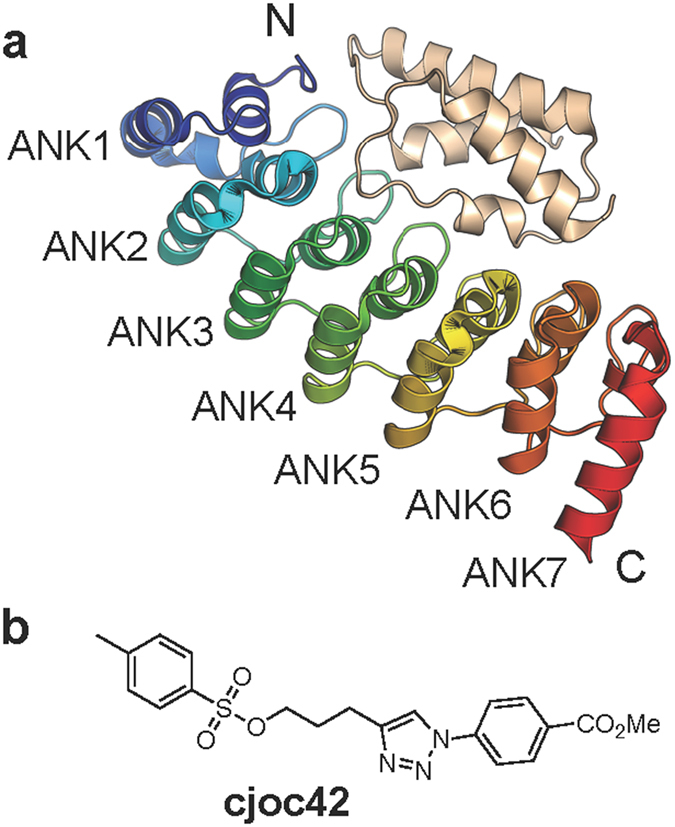
Relevant structures. (**a**) Schematic of the structure of gankyrin (in colour) complex with S6C. (**b**) Chemical structure of cjoc42.

Small molecules from diversity-oriented synthesis (DOS) libraries generated in-house were screened in a thermal shift assay to determine their ability to bind gankyrin^16^. Experiments were performed at a protein concentration of 20 μM and a compound concentration of 300 μM. Primary hits were identified as compounds causing an increase in melting temperature ≥0.5 °C. The screening process led to the identification of a hit molecule, designated as cjoc42 (Fig. 1b). See Supplementary Fig. S2 for thermal shift assay data.

### Analysis of the gankyrin-cjoc42 interaction

#### Thermodynamic characterization of the gankyrin-cjoc42 interaction

**Figure 2:**
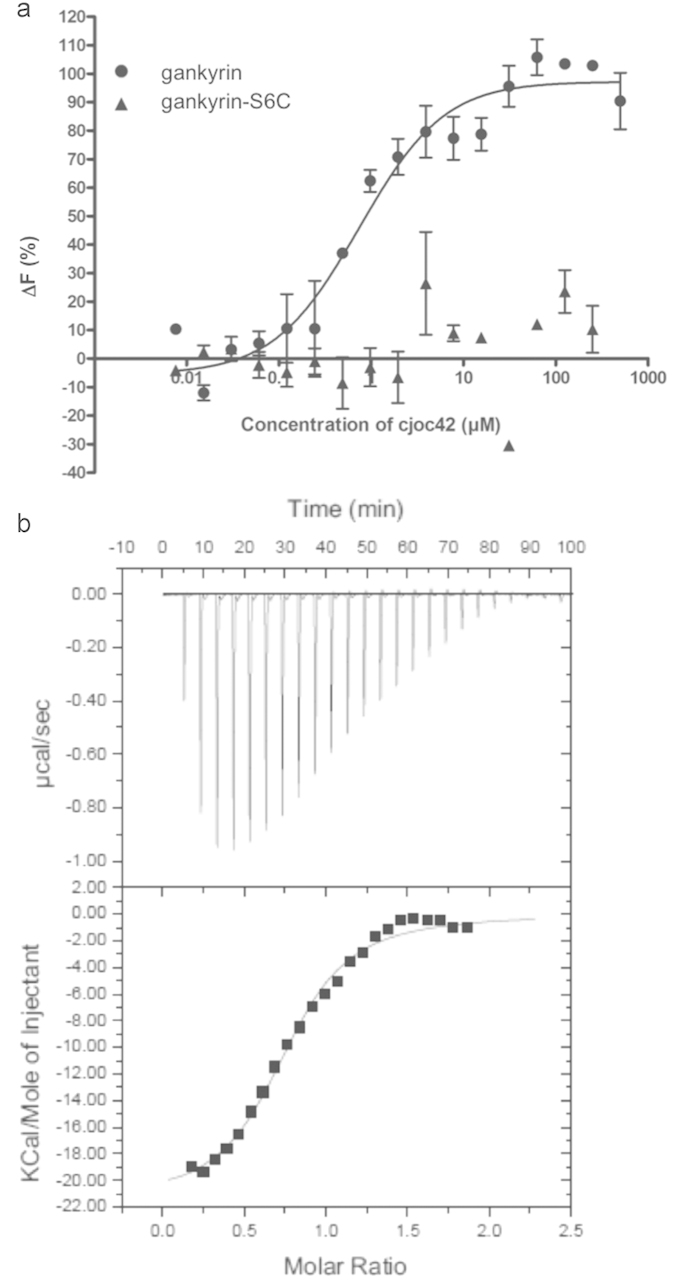
Thermodynamic characterization of the gankyrin-cjoc42 interaction. (**a**) Titration of cjoc42 into gankyrin and the gankyrin-S6C preformed complex, measured by MST. The protein concentration was 5 μM. (**b**) ITC trace of cjoc42 (100 μM) titrated into gankyrin (10 μM).

Label-free microscale thermophoresis (MST) was employed to determine the affinity of cjoc42 for gankyrin. Using this method, a dissociation constant (*K*_d_) of 630 ± 40 nM was obtained (Fig. 2a). Given the relevance of the gankyrin-S6 interaction to cancer, we were interested in exploring the possibility that cjoc42 and S6 bind to the same region of gankyrin. It is not possible to express S6C (or even full-length S6) in isolation from gankyrin (in a non-grafted form), as it appears that S6 must be bound to gankyrin to remain in solution^10^. Instead, cjoc42 was titrated into the preformed complex of gankyrin and S6C. No detectable binding was observed, and this result suggests that the binding sites on gankyrin for cjoc42 and S6C overlap with each other (Fig. 2a).

The interaction between cjoc42 and gankyrin was also measured using isothermal calorimetry (ITC, Fig. 2b), and values for the *K*_d_ of 580 ± 70 nM and stoichiometry of 0.8 ± 0.1 were obtained. To investigate the specificity of the interaction, cjoc42 was titrated into the ankyrin-repeat protein p16, which has sequence similarity to gankyrin and which like gankyrin also binds to CDK4. p16 had no detectable affinity for cjoc42 (not shown).

**Figure 3:**
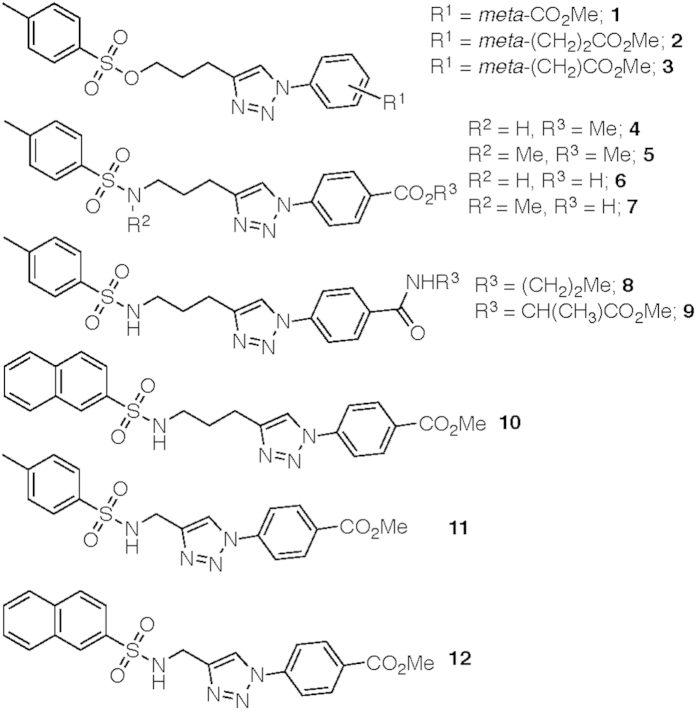
Analogues of cjoc42. See Supplementary Information for full synthetic details.

A focused library of 12 analogues of cjoc42 (compounds **1–12**, Fig. 3, see Supplementary Information for synthetic procedures) was then generated to explore the chemical space around the hit scaffold.

In addition to three alternative sulfonic esters, the compound set also included nine sulfonamides. These varied in: (i) the nature of the substituent on the phenyl ring linked directly to the triazole ring (methyl ester in **4**,**5**,**10–12**, free acid in **6–7** and amide in **8–9**); (ii) the length of the linker between the triazole ring and the sulfonamide amine (compare **4–10** to **11–12**); (iii) the alkylation state of the sulfonamide nitrogen (compare **6–7** with **4**,**5**,**8–12**); and (iv) the nature of the aromatic portion of the sulfonamide moiety (*para*-tolyl in **4–9** and **11**, naphthyl in **10** and **12**). The cjoc42 analogues (and late-stage synthetic intermediates) were subsequently screened using ITC to determine their affinities for gankyrin. Of these compounds only **8** and **12** were found to have moderate binding affinities to gankyrin (*K*_d_ values of approximately 10 μM and 56 μM, respectively, as determined by ITC, see Supplementary Fig. S3 and S4, respectively). In addition to these analogues, a series of tosylate fragments were screened, based on the premise that the sulfone portion of cjoc42 may be a significant contributor to its overall binding affinity for gankyrin (see Supplementary Figs S5–S8). However, all the tosylate fragments were found to bind very weakly (*K*_d_ values greater than 200 μM as determined by ITC, see Supplementary Fig. S4). Overall, none of the compounds examined were found to have a stronger binding affinity for gankyrin than did cjoc42. Thus, attention turned towards further biophysical analysis of the binding of cjoc42 to gankyrin.

#### NMR analysis of the gankyrin-cjoc42 interaction

**Figure 4:**
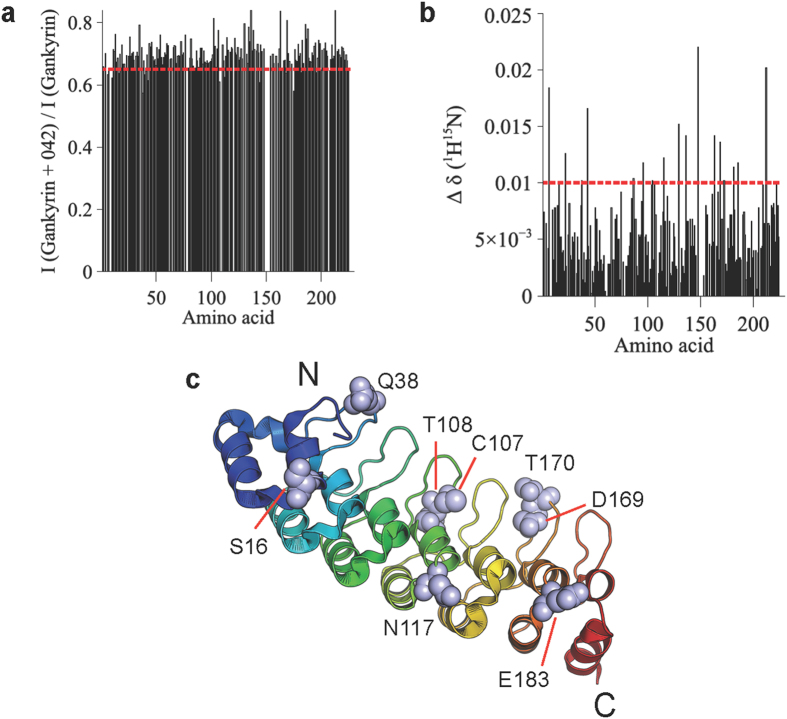
NMR analysis of the gankyrin-cjoc42 interaction. (**a**) Changes in cross-peak intensity of the amide backbone of gankyrin in the presence of cjoc42. (**b**) Changes in chemical shifts of the amide backbone of gankyrin in the presence of cjoc42. (**c**) Residues 16, 38, 107, 108, 117, 169, 170, 183, with chemical shift changes (Δδ (^15^N-^1^H)) of >0.01 that also show significant intensity changes in the presence of cjoc42, are shown on the gankyrin structure.

The previously reported solution NMR structure of gankyrin^2^ was used to obtain information about the binding interface of cjoc42 on gankyrin (Fig. 4). NMR data showed changes in both chemical shifts and line broadening for a subset of gankyrin residues in the presence of cjoc42, which, when mapped onto the structure, reveal a number of solvent-exposed clusters located along the concave surface at which S6C binds (Fig. 4).

The fact that we observe by ITC an approximate 1:1 stoichiometry for the gankyrin-cjoc42 interaction suggests that cjoc42 binds to one site rather than multiple sites; the delocalized nature of the residues found by NMR to be perturbed in the presence would then indicate that the effect of cjoc42 binding at one site is propagated to distant sites throughout the protein’s length. This finding is consistent with long-range effects of ligand binding that have previously been observed both for repeat proteins^17^,^18^ and for numerous other proteins^19^,^20^,^21^,^22^,^23^.

#### In silico analysis of the gankyrin-cjoc42 interaction

**Figure 5:**
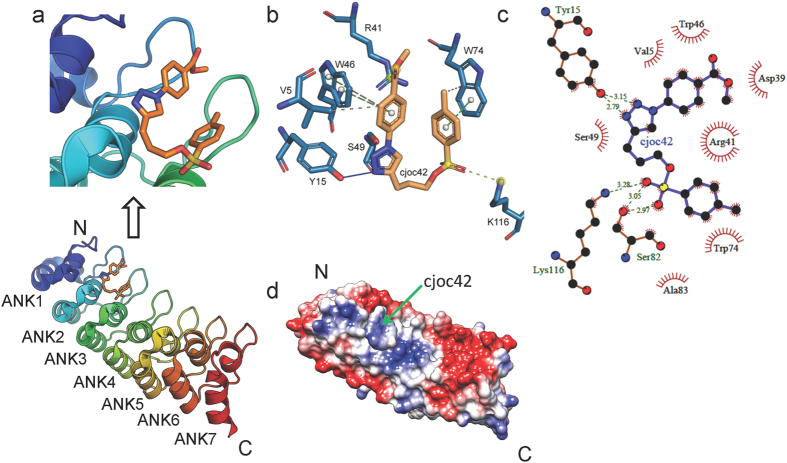
*In silico* analysis of the gankyrin-cjoc42 interaction. (**a**) The docking site of cjoc42 on gankyrin (pdb 1QYM) with a close-up of the interacting interface shown above. The pose represents the top ranked (i.e. lowest energy, ∆*G* = −6.3 kcal/mol) pose obtained from blind docking using AutoDock Vina. Similar results were obtained using AutoDock 4.2 and EADock DSS. (**b**,**c**) Ligand interaction diagram of the docked pose of cjoc42 on gankyrin. The diagrams were generated using PLIP (**b**) and LigPlot+ (**c**). (**d**) The electrostatic surface potential representation of gankyrin with cjoc42 docked onto it. Red, blue, and white represent acidic, basic, and neutral (hydrophobic) regions, respectively, with the sliding colour scale (±5 kTe^−1^, where k is the Boltzmann constant, T is temperature and e is the elementary charge) below indicating the charge distribution across the gankyrin surface.

The NMR experiments showed chemical shift changes and line broadening of clusters of solvent-exposed residues of gankyrin in the presence of cjoc42. To further delineate the cjoc42-binding site on gankyrin, molecular docking was carried out in an unbiased approach (blind docking) with three independent docking algorithms (AutoDock Vina, AutoDock 4.2 and EADock DSS) applied with multiple repeats (Fig. 5 and Supplementary Figs S9 and S10). By overlaying the docked structure with the gankyrin-S6C crystal structure (Supplementary Fig. S11) we can see that the top-ranked poses (i.e. the ones with the lowest free energy of binding) in all of the runs showed cjoc42 docking onto a region of the gankyrin surface at which S6C makes contacts *via* the latter’s exposed loops (specifically S6 residues ^355^SEEVD^359^ and ^393^I-L^395^) (Fig. 5a and Supplementary Fig. S11). The electronegative EEVD loop interacts predominantly with an electropositive patch on the surface of gankyrin. Supplementary Fig. S11 shows that cjoc42 overlays on this loop and docks close to the electropositive patch of gankyrin.

Ligand interaction diagrams constructed using PLIP (Fig. 5b) and LigPlus (Fig. 5c) show that the two nitrogen atoms from the triazole ring engage in hydrogen bonding with the phenolic hydroxy group of Tyr 15. The indole ring of Trp74 and the aromatic ring of the tosylate moiety of cjoc42 engage in a π-π stacking interaction, whilst the sulfonyl oxygen of the tosylate group forms hydrogen bonds with the amino group of Lys116 and hydroxyl group of Ser82. The phenyl ring linked directly to the triazole ring appears also to engage in a π-π stacking interaction with the indole ring of Trp46.

Inspection of the docked cjoc42 pose shows that the tosyl group of cjoc42 is positioned in such a way as to serve as surrogate for the carbonyl groups of Glu356 and Glu357 of the S6 ^355^SEEVD^359^ motif. The aromatic methyl-phenyl moiety of cjoc42 is poised similarly to mimic the Val358 of the S6 ^355^SEEVD^359^ motif. The other phenyl group of cjoc42 (that is linked directly with the triazole ring) is poised similarly to mimic residue Leu395 of S6 (Supplementary Fig. S11). Thus some atoms of cjoc42 in its docked pose mimic the spatial distribution of the critical residues of the exposed loop of S6C that interact with mGankyrin, adopting a 3D shape that is largely complementary to the local cavity (Supplementary Fig. S9). It is worth noting that this site (i.e. the cjoc42-interacting site on hGankyrin/the S6 ‘SEEVD’-interacting site on mGankyrin) is predicted by FTMap^24^ as the most likely hot-spot on the gankyrin surface for interaction with small molecules or proteins. Indeed, a number of proteins, which, like S6, have an EEVD motif, were recently shown to interact with gankyrin^25^. Given the electropositive/electroneutral nature of the site, as shown in the surface electrostatics representation (Fig. 5d), it seems entirely plausible that electronegative EEVD peptides as well as the sulfonyl group of the cjoc42 can be accommodated there.

#### Control experiments

A number of control experiments were carried out to address the possibility of cjoc42 being a false positive hit from the thermal shift screen (see Supplementary Information). Mass spectrometry was used to test for the possibility of the binding observed in our biophysical assays being due to a covalent attachment between gankyrin and cjoc42. Time-of-flight (TOF) electrospray mass spectrometry spectra of gankyrin alone (50 μM) and gankyrin (50 μM) incubated with cjoc42 (300 μM) showed a single major peak corresponding to the molecular weight of unconjugated gankyrin (see Supplementary Fig. S12). No peak corresponding to alkylated protein was observed, indicating that cjoc42 does not bind in a covalent manner to gankyrin. Compound cjoc42 at a concentration of 200 μM was found to have no effect on two unrelated and well-characterized PPIs (Rad51-BRC4 and Aurora A-TPX2) in a fluorescence polarization assay (see Supplementary Fig. S13), indicating that cjoc42 does not show non-specific binding. In addition, before optimizing the cellular assays that used the osteosarcoma U2OS cell line as a model system (*vide infra*), potential system-wide effects of cjoc42 addition to U2OS cells were investigated. No changes in mitotic index (see Supplementary Fig. S14) or cell viability (see Supplementary Fig. S15) were observed (compound concentrations up to 50 μM), indicating that any observed changes that might result from the addition of cjoc42 are unlikely to be due to non-specific adverse effects related to cell proliferation or toxicity.

### Cellular activity of cjoc42

Collectively, the biophysical experiments strongly indicated that cjoc42 binds to the putative PPI interface of gankyrin at the region where the protein interacts with the S6 binding partner. We turned our attention next towards evaluating the cellular activity of cjoc42 by determining its effect on p53 levels and activity in the osteosarcoma U2OS cell line as a model system.

#### cjoc42 counteracts gankyrin-induced decrease in p53 protein levels

**Figure 6:**
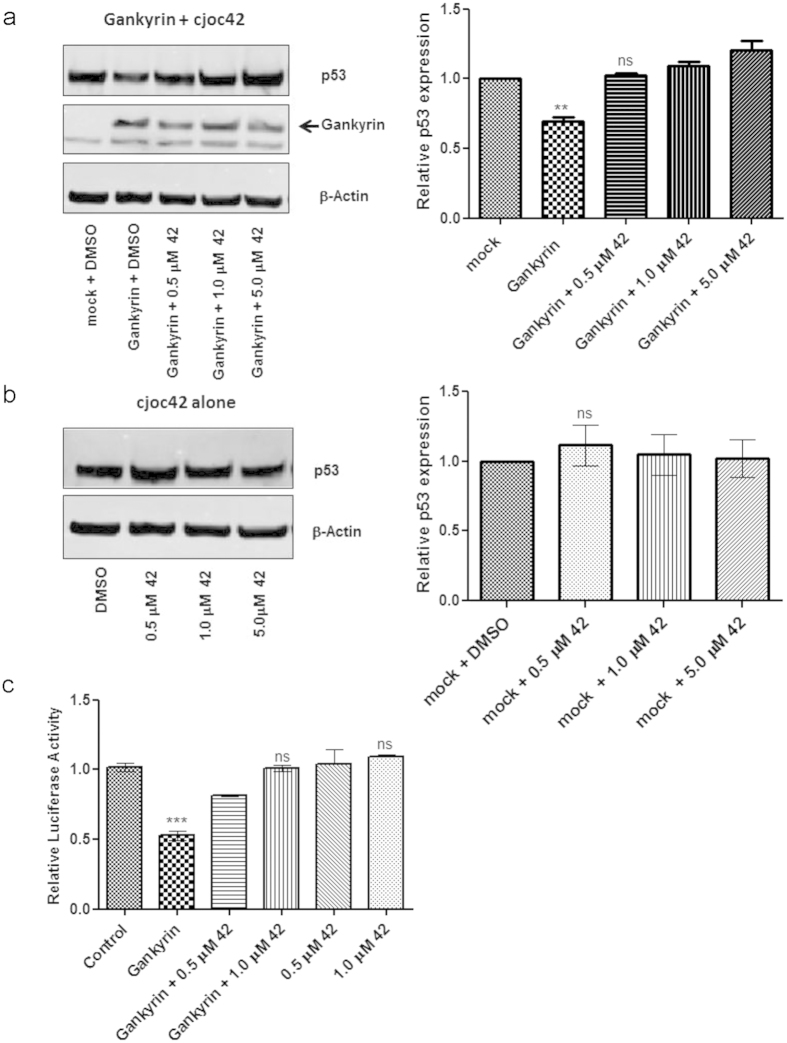
Cellular activity of cjoc42 (abbreviated to ‘42’). In (**a**,**b**) the left-hand figures show representative images and the right-hand figures show densitometry analysis of band intensities (n = 3 and SEM). All data were normalized to untreated mock-transfected cells, and all statistical comparisons (t tests) were made to these. ns, non-significant; *p < 0.05; **p < 0.01; ***p < 0.001; (**a**) Effects of gankyrin over-expression on levels of p53 and β-actin in U2OS cells 48 hours post transfection with 2 μg gankyrin plasmid, p53 expression levels are reduced by ~40% compared with control cells. cjoc42 (abbreviated to ‘42’) addition at the time of transfection inhibits this decrease in p53 levels. (**b**) Cells treated with cjoc42 alone do not show any changes in p53 levels compared to mock transfections. (**c**) Reduction of p53 luciferase reporter activity in gankyrin-overexpressing U2OS cells can be inhibited by cjoc42 (abbreviated to ‘42’) in a dose-dependent manner. cjoc42 alone at equivalent doses does not alter the luciferase reporter activity.

The effects of cjoc42 in gankyrin-overexpressing cells were first examined. FLAG-tagged gankyrin over-expression was first optimised in the (p53 normal) osteosarcoma cell line U2OS to enable the detection of small changes in intracellular protein levels in whole cell lysates 48 hours post transfection, a significant reduction in p53 levels could be detected without any change in the levels of housekeeping protein β-actin (Fig. 6a). When the gankyrin-overexpressing cells were incubated with cjoc42, a dose-dependent restoration of p53 levels (comparable to mock transfected cells) could be seen. There was no significant change in p53 levels when cells were treated with cjoc42 alone without gankyrin (Fig. 6b). Thus, it can be concluded that cjoc42 is capable of inhibiting gankyrin-induced lowering of p53 levels in cells.

It was found that p53 levels are also affected by the addition of cjoc42 in the liver cancer cell line HepG2 where gankyrin expression is a key oncogenic driver, indicating that the compound can affect endogenous gankyrin in a manner similar to that observed for gankyrin over-expressed by transfection (Supplementary Fig. S16). Thus, with a view to studying the effects of the compound in a cellular environment with normal p53 background and where gankyrin expression (+/−) and levels can be modulated at will, we chose to perform the subsequent functional assays in the transfection-amenable cell line U2OS. We further found that cjoc42 does not alter the mRNA levels of p53 or housekeeping genes like GAPDH (Supplemental Fig. S17), indicating that the compound does not exert its cellular effect on p53 at the level of transcription.

#### cjoc42 inhibits gankyrin-induced decrease in p53 luciferase reporter activity

A p53 luciferase reporter assay was used to examine the downstream consequences of reduction in p53 protein levels due to gankyrin overexpression. Reduced p53 levels lead to a decrease in the binding of p53 to its cognate site on a co-transfected luciferase reporter construct, which results in reduction of luciferase activity compared to mock-transfected cells. The addition of cjoc42 to gankyrin-overexpressing U2OS cells was able to prevent this reduction of luciferase activity. This result suggests that gankyrin-mediated decrease in p53 reporter binding is inhibited by the presence of cjoc42. Addition of cjoc42 alone did not affect reporter activity when compared with mock-transfected cells, confirming that the effect of cjoc42 is gankyrin-dependent (Fig. 6c).

#### cjoc42 inhibits gankyrin-induced decrease in p53 downstream target expression and resensitises cells to DNA damage

**Figure 7:**
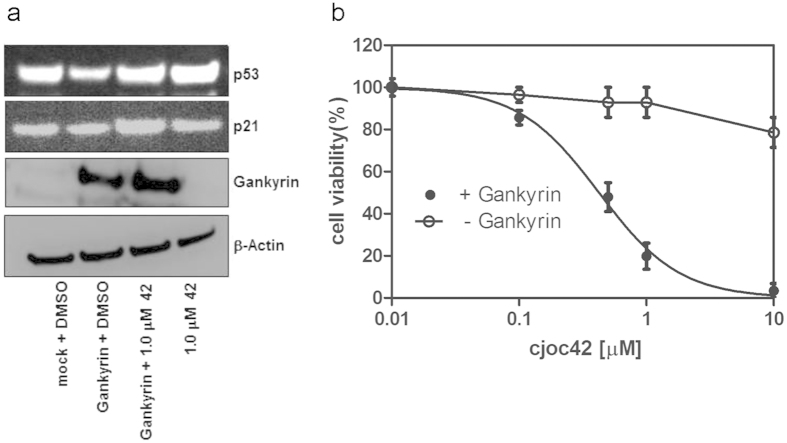
cjoc42 (abbreviated to ‘42’) inhibits gankyrin-induced decrease in p53 downstream target expression and resensitises cells to DNA damage. (**a**) Four hours after DNA damage with Etoposide a reduction of p21 upregulation is seen in gankyrin-overexpressing U2OS cells (lane 2) compared to mock transected cells (lane 1). This can be prevented by cjoc42 addition (lane 3). cjoc42 alone shows upregulation of p21 (in the presence of DNA damage) to levels comparable with mock-transfected cells (lane 4). (**b**) Cell viability (expressed as a percentage of control) 24 hours after Etoposide treatment. Gankyrin-overexpressing U2OS cells incubated with cjoc42 show a dose-dependent loss of viability. Cells not expressing gankyrin (— Gankyrin) show no such dose-dependent change in cell viability.

Etoposide concentrations were first optimized at 0.2 μM to trigger DNA damage, resulting in loss of cell viability. Next, assays were performed using these conditions, for gankyrin-transfected or mock-transfected U2OS cells with simultaneous cjoc42 incubation. Gankyrin-transfected cells showed a modest reduction in the levels of p21 (a p53 downstream target) expression, which was restored upon cjoc42 addition (Fig. 7a). Mock-transfected cells did not show significant changes in p21 expression when exposed to cjoc42 (Fig. 7a). Both the Trypan blue dye exclusion assay (Invitrogen) and the Cell Titre Glo cell viability assay (Promega) showed that there was enhanced cell viability for gankyrin-transfected cells compared to mock-transfected cells. Simultaneous incubation with cjoc42 was able to restore sensitivity to DNA damage (as indicated by lower cell viability) in the gankyrin-overexpressing cells in a dose-dependent manner (Fig. 7b). Cells incubated with cjoc42 alone showed no dose-dependent change in cell viability, indicating that the loss in cell viability induced by cjoc42 is mediated by gankyrin.

## Discussion

Herein, we report the discovery of cjoc42, a novel small molecule that is capable of binding directly to the oncoprotein gankyrin. Cell-based experiments showed that cjoc42 is not toxic. In cells overexpressing gankyrin, cjoc42 in a dose-dependent manner prevents the decrease in p53 protein levels normally associated with high amounts of gankyrin, and it restores p53-dependent transcription and sensitivity to DNA damage. Our data suggest that cjoc42 targets the proposed PPI interface of gankyrin in the region where the protein interacts with its binding partner S6; the compound does not appear to modulate PPIs non-specifically. Thus cjoc42 represents the first selective small-molecule binder and modulator of gankyrin activity, and our study provides proof-of-principle that small molecules that target the PPI surface of gankyrin are able to inhibit its cellular function.

Gankyrin is a central regulatory hub for numerous proteins involved in p53 and pRb tumour suppressor pathways. Neither the locations of the binding sites for these proteins on gankyrin nor the dynamics between them *in vivo* are known; however, given that cjoc42 produces a cellular effect at low micromolar concentrations, we propose that cjoc42 binds to and increases the pool of intracellular gankyrin that is not complexed with S6; cjoc42 is thereby simultaneously able to block and/or disrupt interactions of this surface with partner proteins too. Of note in this regard, McNaughton and colleagues recently reported the attenuation of the ubiquitination function of the gankyrin-MDM2 complex *in vitro* upon addition of a synthetic gankyrin-binding protein designed to mimic S6C^14^, but in the absence of structural information about the gankyrin-MDM2 complex it is unclear how this S6-mimetic protein achieves this effect. Compound cjoc42 represents a small-molecule starting point that targets the S6-binding interface of gankyrin and that is able to enhance p53 levels and activity in the cell, likely doing so by modulating the relative compositions of gankyrin complexes with its various partner proteins and shifting the equilibrium between them. Compound cjoc42 is thus both a valuable chemical probe with which to investigate the spatio-temporal sequence and dynamics of such gankyrin-partner protein complex formation and also a starting point for the development of a small-molecule anticancer therapy directed against gankyrin.

## Methods

### Protein expression

The *E. coli* expression construct for His-tagged full-length human gankyrin was a kind gift from A. J. Wilkinson (University of York, UK). The protein was purified as described previously^1^. The genes encoding human gankyrin and S6C were cloned into pETDuet1 (Novagen). The complex was purified as previously described^13^. The buffer used in all subsequent experiments was 20 mM sodium borate buffer, pH 7.5, unless stated otherwise.

### Thermal shift assay

The thermal shift assay was performed in a 96-well plate with 20 μM gankyrin in buffer containing Sypro Orange (Invitrogen). Compounds were added, from a master stock of 10 mM to obtain a final concentration of 300 μM, keeping the DMSO concentration at 5% vol/vol in all wells. All measurements were done in triplicates. Unfolding of gankyrin was monitored between 15 °C and 65 °C and melting curves were generated by plotting SYPRO Orange fluorescence as a function of temperature. The resulting data were fitted to obtain the melting temperature T_m_ (point of sigmoidal inflection) as the maximum of each curve’s derivative. A hit was defined as a compound that increased the T_m_ by at least 0.5 °C.

### Microscale thermophoresis (MST)

MST measurements were made using the Monolith NT.Label.Free device with standard treated capillaries (NanoTemper Technologies) and the manufacturer’s standard settings (Laser-power 20%, Laser-on time 60s, LED power 20%). Measurements were performed in 20 mM Na-Borate buffer, pH 7.5, 10% DMSO. The relative change in fluorescence intensity due to thermophoresis was measured by varying the concentration of cjoc42 (a 1:1 serial dilution series) against a fixed concentration of gankyrin or gankyrin-S6C complex (5 μM).

### Isothermal titration calorimetry (ITC)

Gankyrin samples were dialysed overnight into 20 mM sodium borate buffer pH 7.5. cjoc42 (or other compounds) solution was adjusted to the appropriate concentration using the same dialysis buffer. DMSO concentration was added to 5% vol/vol (final) for both protein and compound samples. ITC measurements were performed at 25 °C on a Microcal VP ITC calorimeter (Malvern, UK) with gankyrin in the sample chamber and compound added via the syringe using 30 successive additions of 10 μl for 12 s (with an initial injection of 5 μl for 6 s). The interval between each injection was 240 s. Data were analysed using the instrument’s ORIGIN analysis software.

### NMR spectroscopy analysis of the gankyrin-cjoc42 interaction

NMR samples comprised 100 μM gankyrin in buffer containing 5% dimethyl sulfoxide (DMSO), 10% D_2_O and 0.01% 4, 4-dimethyl-4-silapentane-1-sulfonic acid (DSS). Cjoc42, when included, was at a concentration of 100 μM. 2D ^1^H-^15^N HSQCs were monitored at 300 K on a Bruker Avance 700 MHz Ultrashield spectrometer equipped with a TXI cryoprobe. NMR data were processed using NMRPipe^26^ and analyzed using Sparky. The changes in chemical shift (Δδ (^15^N-^1^H)) and signal intensity (ΔI) of the backbone amide group of each residue of gankyrin were calculated using equations (1) and (2), respectively:


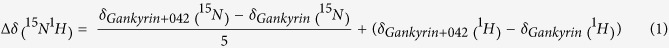



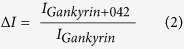


where *δ*_*Gankyrin*_(^15^*N*), *δ*_*Gankyrin*_(^1^*H*) *δ*_*Gankyrin*+042_(^15^*N*) and *δ*_*Gankyrin*+042_(^1^*H*) are the ^15^N and ^1^H chemical shifts of the peptide amide group of each residue of gankyrin when the protein in the absence and presence of cjoc42, and *I*_*Gankyrin*_ and *δ*_*Gankyrin*+042_ are the intensities of the peptide amide group of each residue of gankyrin in the absence and presence of cjoc42.

### *In silico* methods

Docking experiments were performed using a blind docking approach and several software packages based on different algorithms. These include AutoDock Vina version 1.1.2 (http://vina.scripps.edu/)^27^, AutoDock 4.2 (http://autodock.scripps.edu/) and EADock DSS implemented in the SwissDock server (http://www.swissdock.ch/)^28^. In each case, docking was performed as three independent runs to assess reproducibility and only the lowest energy pose was considered for each case. For detailed inspection and analyses of the docked poses, ligand interaction diagrams were generated using LigPlot + version 1.4^29^ and Protein-ligand interaction profiler (PLIP)^30^. FTMAP server (http://ftmap.bu.edu/)^24^ was used for predicting the hot spots for ligand binding on the gankyrin surface.

### Cell culture, gankyrin overexpression and DNA damage

U2OS cells were grown using standard cell culture conditions as described in Higashitsuji^9^ and with the same FLAG-gankyrin overexpression vector (Agilent Technologies, Santa Clara, CA) kindly provided by Dr J. Fujita (Japan). Cells were transiently transfected with 2 μg of this construct using Lipofectamine 200 (Life Technologies) with 1 × 10^6^ cells per transfection. Gankyrin, p53, p21 and β-actin expression levels were subsequently assessed at 24–72 h by Western blot both with and without incubation of varying concentrations of cjoc42 that had been freshly dissolved in 0.5% DMSO (final). DNA damage, where necessary, was induced with 0.2 μM etoposide treatment, 24 hours post gankyrin transfection. Control transfections were performed simultaneously using 2μg pmaxGFP expression vector (Amaxa) to determine transfection efficiency.

### p53 luciferase reporter assay

U2OS cells were plated at 2 × 10^4^ cells/well of a 96 well plate 1 day before the transfection. The p53 promoter-reporter plasmid PGL4.38 (Promega), an internal control plasmid, pRL-TK (Promega) and the FLAG-gankyrin expression plasmid, were co-transfected into cells the next day with or without the addition of cjoc42 in DMSO (1 μM final concentration). Control wells had 1% DMSO alone. The cells were harvested for the luciferase assay 48 hours post transfection. Luciferase activity was measured by using the dual-luciferase reporter assay system (Promega) following the manufacturer’s instructions. The results were analysed as the fold induction of the reporter plasmid alone after normalization with the internal control plasmid pRL-TK for each transfection condition, and they are presented as normalized change relative to mock-transfected cells.

### Cell viability assay

Cell viability was assessed using the Cell Titre Glo assay (Promega) coupled to a Tecan Infinite F200 microplate reader and the Trypan Blue dye exclusion assay coupled to the Countess cell counter (Invitrogen) following manufacturer’s instructions, after FLAG-gankyrin/mock transfection, incubation with cjoc42 or DMSO vehicle and DNA damage with 0.2 μM Etoposide. All assays were performed in triplicate, and the data were normalised to vehicle-treated control cells.

### Western blotting

Whole cell lysates were subjected to SDS-PAGE and proteins transferred onto polyvinylidene fluoride membranes. The following primary antibodies were used: Gankyrin and p53-DO1 (Santa Cruz Biotech), p21and β-actin (Abcam, Cambridge, UK). Secondary HRP-conjugated antibodies were from Dako. Luminograms were obtained with Chemiluminescence Detection kits (GE Healthcare, Uppsala, Sweden, Thermo Scientific), and the densitometry of the blots was performed using ImageJ software^31^.

### qPCR experiments

Total RNA was extracted from gankyrin over-expressing U2OS cells treated with 1 μM cjoc42 or DMSO (control) for 48 hours using the RNeasy Mini kit (Qiagen). Relative mRNA transcript levels were measured in triplicate using the Lightcycler 480 Sybr Green 1 kit and the LightCycler(Roche), following reverse transcription of 1 μg total RNA. Primers were obtained from Qiagen (Quantitect primers). p53 expression levels were assessed in terms of the threshold cycle (Ct) using GAPDH as the housekeeping gene for comparison.

### Control experiments

Potential disruption of the RAD51-BRC4 and Aurora A-TPX2 PPIs by cjoc42 was analyzed using fluorescence polarization, as described elsewhere^32^. cjoc42 was tested in human osteosarcoma cells (U2OS) for its effect on cell proliferation and for its ability to induce mitotic arrest (the latter assay uses a high content screening approach wherein cells are stained with an antibody against the mitotic marker phospho-Histone H3 (pH3) that allowed a calculation of the percentage of mitotic cells), as described previously^32^.

## Additional Information

**How to cite this article**: Chattopadhyay, A. *et al.* Discovery of a small-molecule binder of the oncoprotein gankyrin that modulates gankyrin activity in the cell. *Sci. Rep.* **6**, 23732; doi: 10.1038/srep23732 (2016).

## Supplementary Material

Supplementary Information
