# Supplementary material for: Discovery of a small-molecule binder of the oncoprotein gankyrin that modulates gankyrin activity in the cell
Source: Sci Rep. 2016 Apr 5;6:23732. doi: 10.1038/srep23732 (PMC4820706; doi:10.1038/srep23732)
Supplement: Supplementary Information [file srep23732-s1.pdf]

Supplementary Information

Discovery of a Small Molecule Binder of the Oncoprotein Gankyrin that Modulates Gankyrin Activity in the Cell

Anasuya Chattopadhyay,[1] Cornelius J. O'Connor,[2] Fengzhi Zhang,[2] Celine Galvagnion,[2] Warren R. J. D. Galloway,[2] Yaw Sing Tan,[2] Jamie E. Stokes,[2] Taufiq Rahman,[1] Chandra Verma,[3,4,5] David R. Spring,[2*] and Laura S. Itzhaki[1*]

[1]Department of Pharmacology, Tennis Court Road, Cambridge CB2 1PD, UK.

[2]Department of Chemistry, Lensfield Road, Cambridge CB2 1EW, UK.

[3]Bioinformatic Institute (A*STAR), 30 Biopolis Street, #07-01 Matrix, Singapore 138671.

[4]School of Biological Sciences, Nanyang Technological University, 60 Nanyang Drive, Singapore 637551.

[5]Department of Biological Sciences, National University of Singapore, 14 Science Drive 4, Singapore 117543.

*Correspondence: lsi10@cam.ac.uk (L. S. I), spring@ch.cam.ac.uk (D. R. S.)

Index

1. **Supplementary Data** 4

Supplementary Fig S1. Structure-based sequence alignment of mouse Gankyrin (pdb: 2DVW) and human Gankyrin (pdb: 1QYM)..4

Supplementary Figure S2. Thermal shift assay data for compound cjoc42................5

Supplementary Figure S3. ITC trace for titration of compound 8 into gankyrin.......6

Supplementary Figure S4. ITC trace for titration of compound 12 into gankyrin.....7

Supplementary Figure S5. ITC trace for compound shown into gankyrin. 8

Supplementary Figure S6. ITC trace for compound shown into gankyrin. 9

Supplementary Figure S7. ITC trace for compound shown into gankyrin. 10

Supplementary Figure S8. ITC trace for compound shown into gankyrin. 11

Supplementary Fig. S9. Shape complementarity between cjoc42 and its putative docking site on human gankyrin (pdb: 1QYM)... 13

Supplementary Figure S11. cjoc42 binding mode compared with that of the SEEVD motif of S6C. 14

Supplementary Figure S12. Mass spectrometry data...15

Supplementary Figure S13. Compound cjoc042 does not affect unrelated PPIs......16

Supplementary Figure S14. Compound cjoc42 does not induce mitotic arrest.......16

Supplementary Figure S15. Compound cjoc42 does not affect cell viability..........17

Supplementary Figure S16. Effects of cjoc42 addition on levels of p53 and β-actin in HepG2 cells that naturally over-express gankyrin..18

Supplementary Figure S17. qRT-PCR analysis of the effects of cjoc42 addition on p53 transcription in U2OS cells transfected to over-express gankyrin........................19

References...20

4. Supplementary Experimental Procedures...21

4.1. Chemical Synthesis: General details and equipment 21

4.2. Chemical Synthseis: General Procedures...22

4.2.1. General Procedure 1...22

4.2.2. General Procedure 2...22

4.2.3. General Procedure 3...23

4.2.4. General Procedure 4...23

4.2.5. General Procedure 5...23

4.3. Chemical Synthesis: Experimental procedures and characterization data...24

4.4 Chemical Synthesis: ^{1}H and ^{13}C NMR spectra...51

1. Supplementary Data

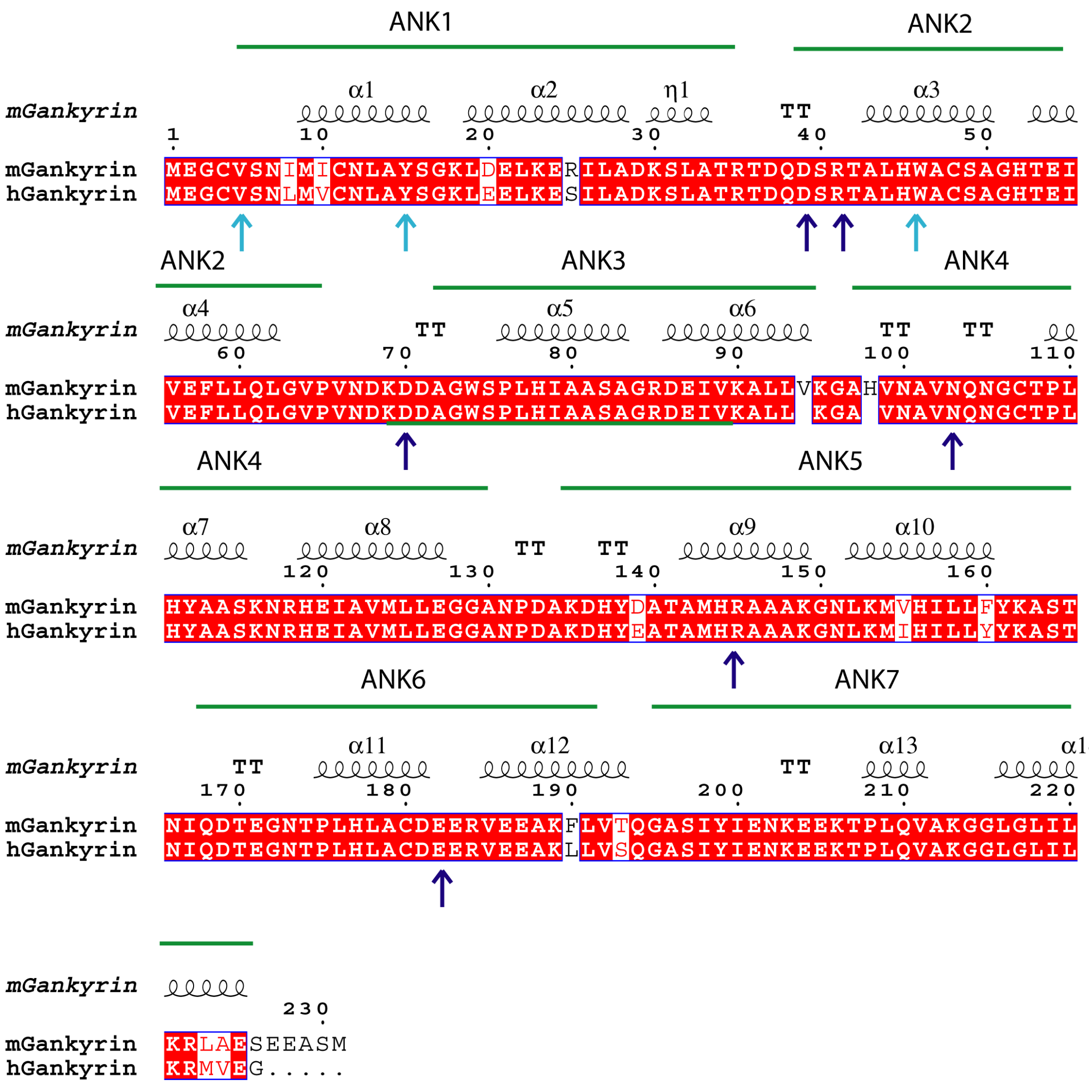

Supplementary Figure S1. Structure-based sequence alignment of mouse Gankyrin (pdb: 2DVW) and human Gankyrin (pdb: 1QYM). Ankyrin repeats 1-7 are denoted ANK1-7. Key residues of mGankyrin implicated in salt bridge formation and hydrophobic interaction with S6C are shown with blue and cyan arrows, respectively.[1] The basic alignment was generated using ESPript 3.0.[2]

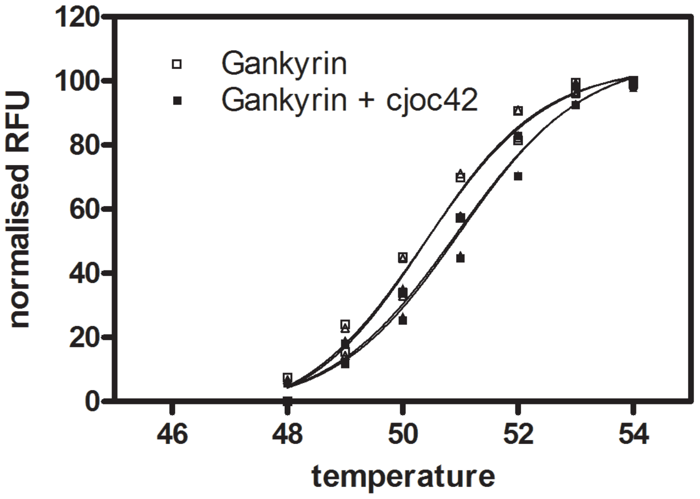

Supplementary Figure S2. Thermal shift assay data for compound cjoc42. Representative plot of thermal unfolding for gankyrin alone and gankyrin in the presence of cjoc042. Thermal denaturation was monitored by SYPRO Orange (2.5x) fluorescence emission intensity.

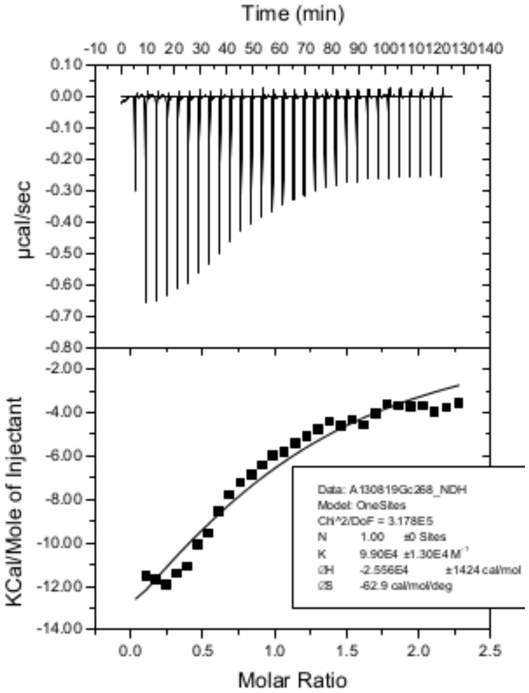

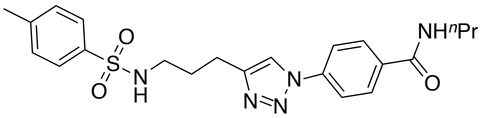

8
N= 1.0 (fixed)
*K*d= 10 μM

Supplementary Figure S3. ITC trace for titration of compound 8 into gankyrin

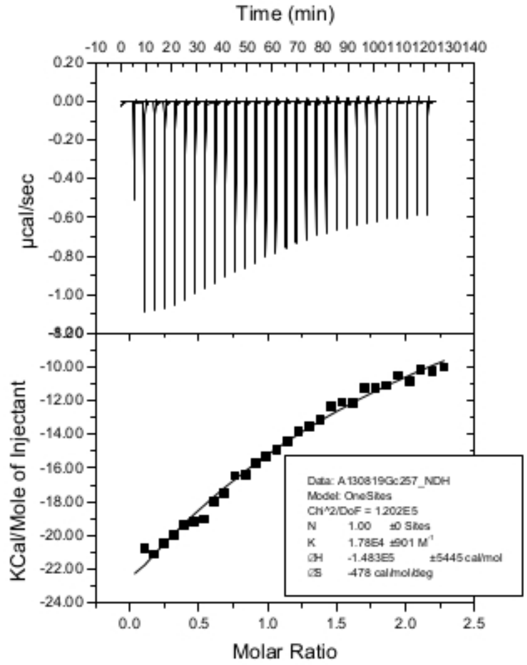

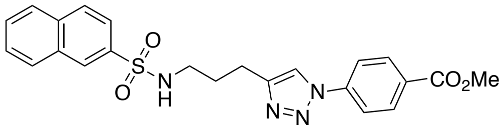

12
N= 1.0 (fixed)
*K*d= 56 μM

Supplementary Figure S4. ITC trace for titration of compound 12 into gankyrin.

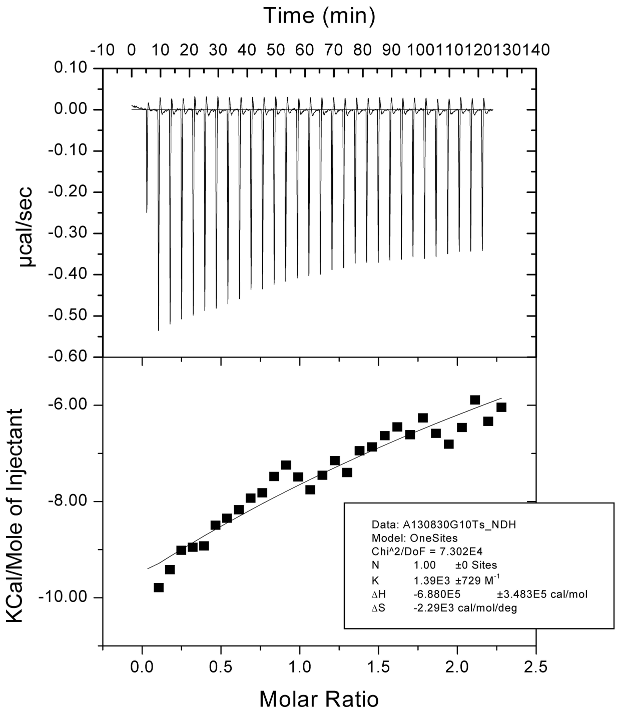

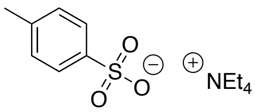

N= 1.0 (fixed)

*K*d= 719 μM

Supplementary Figure S5. ITC trace for compound shown into gankyrin.

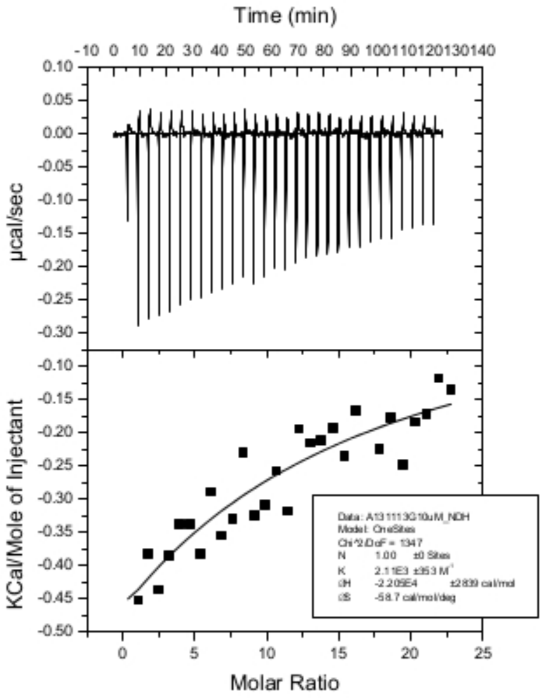

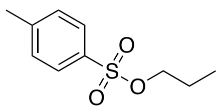

N= 1.0 (fixed)
*K*d= 474 μM

Supplementary Figure S6. ITC trace for compound shown into gankyrin.

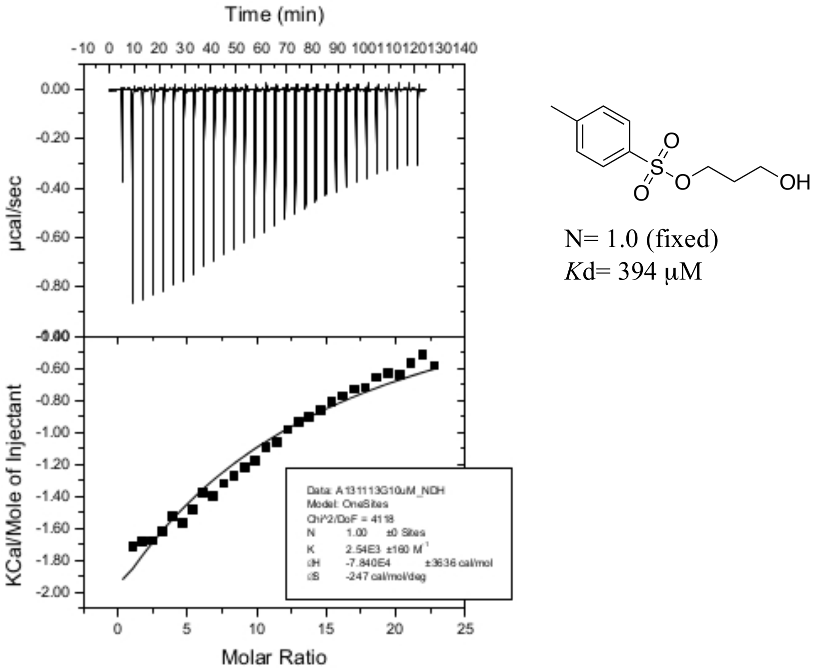

Supplementary Figure S7. ITC trace for compound shown into gankyrin.

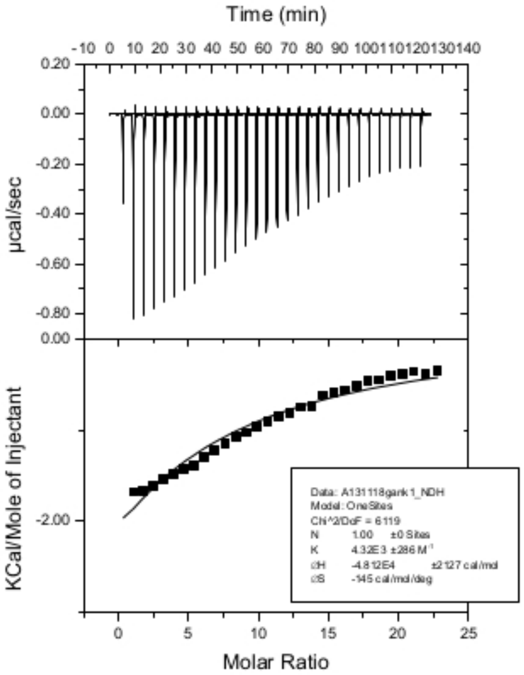

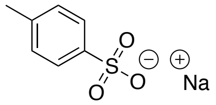

N= 1.0 (fixed)
*K*d= 231 μM

Supplementary Figure S8. ITC trace for compound shown into gankyrin.

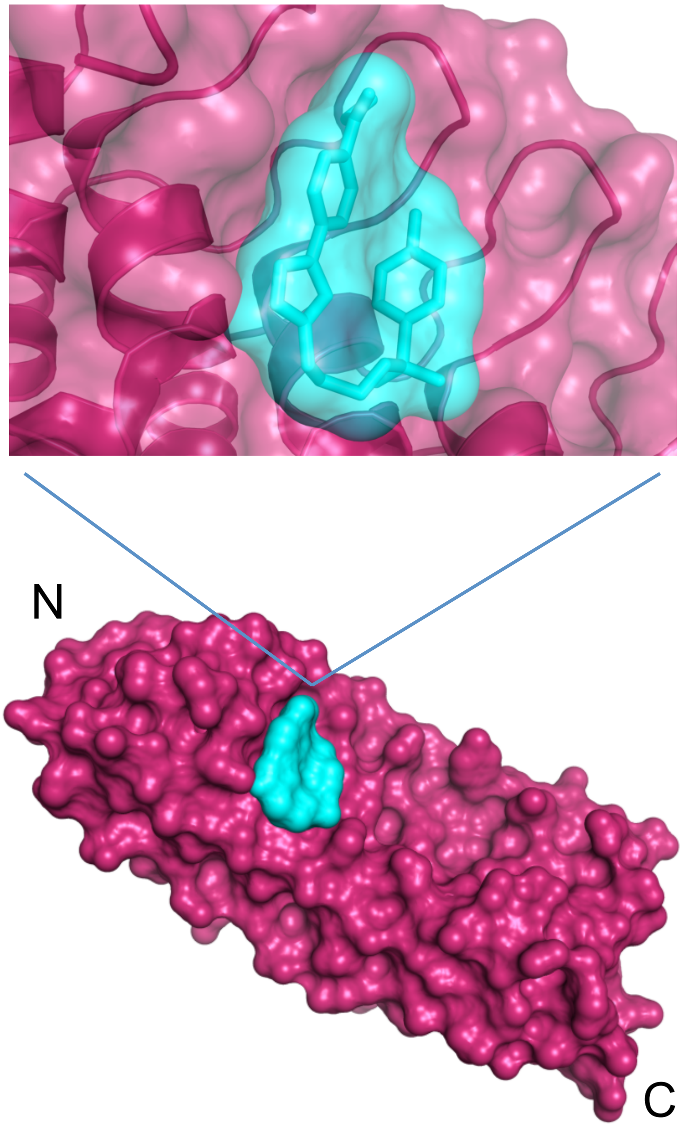

Supplementary Figure S9 Shape complementarity between cjoc42 and its putative docking site on human gankyrin (pdb: 1QYM). Molecular surfaces of cjoc42 (cyan) and hGankyrin (purple), generated by PyMol, are shown.

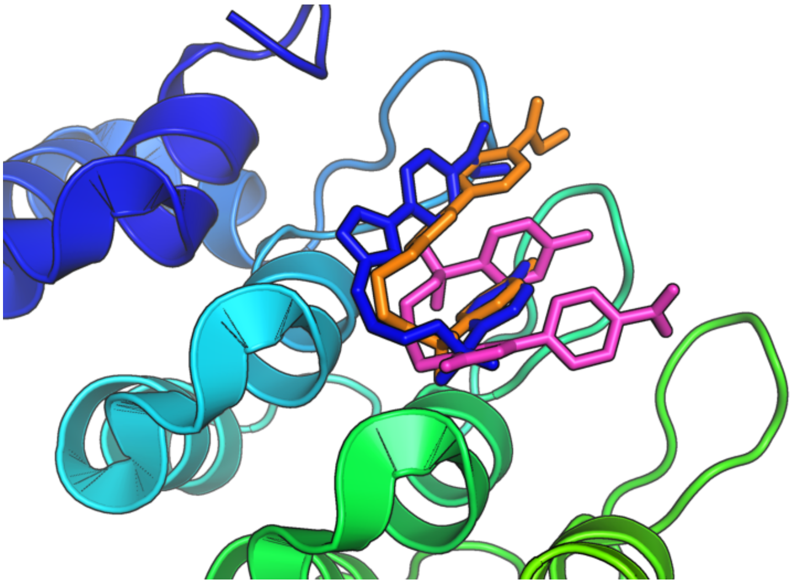

Supplementary Figure S10. Top three poses of cjoc42 docked to gankyrin (pdb: 1QYM). Blind docking was carried out using AutoDockVina (http://vina.scripps.edu/). First, second and third ranked poses are shown as blue, orange and purple sticks, respectively, and the predicted interaction energy values (ΔG) were -6.3 kcal/mol, -6.1 kcal/mol and -5 kcal/mol, respectively. Poses similar to the to the top three poses pose from AutoDock Vina were observed in blind docking with AutoDock 4.2 (ΔG values ranging from -5.0 to -4.2 kcal/mol for the top three poses) and EADock DSS implemented in the SwissDock server ΔG values ranging from -6.9 to -5.9 kcal/mol). The algorithms and the underlying scoring functions for these softwares differ considerably from those of AutoDock Vina, making comparison of ΔG values between softwares impossible; however, the spread is ~1.2 kT, which is reasonably similar at thermal equilibrium. Thus we used the docking approaches to predict the most likely binding site of cjoc42 on gankyrin, with the ΔG values allowing us to rank poses rather than being absolute indicators of the binding affinities.

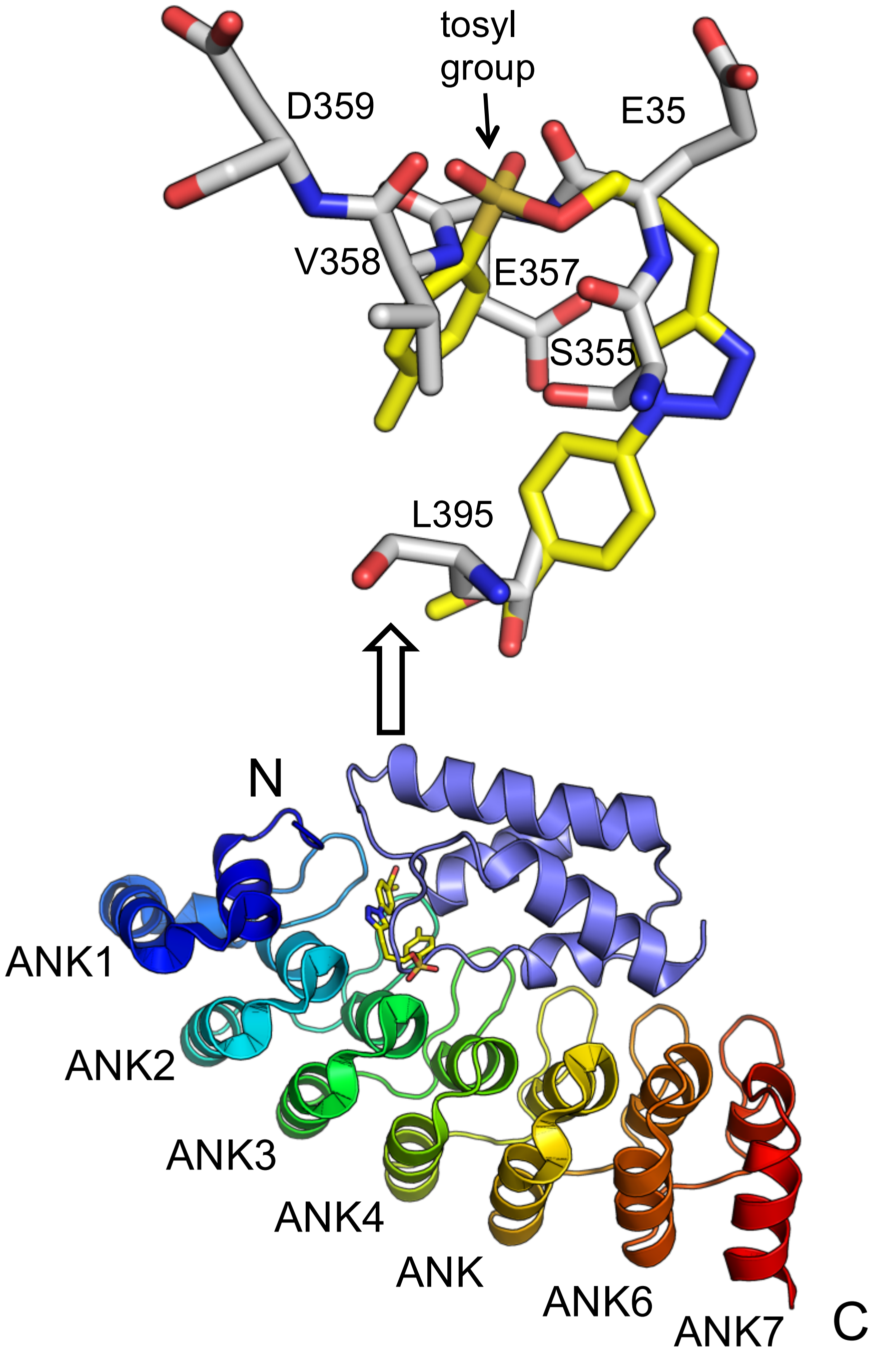

Supplementary Figure S11. cjoc42 binding mode compared with that of the SEEVD motif of S6C. The latter was generated by superimposing cjoc42-bound hGankyrin with S6C-bound mGankyrin.[3]

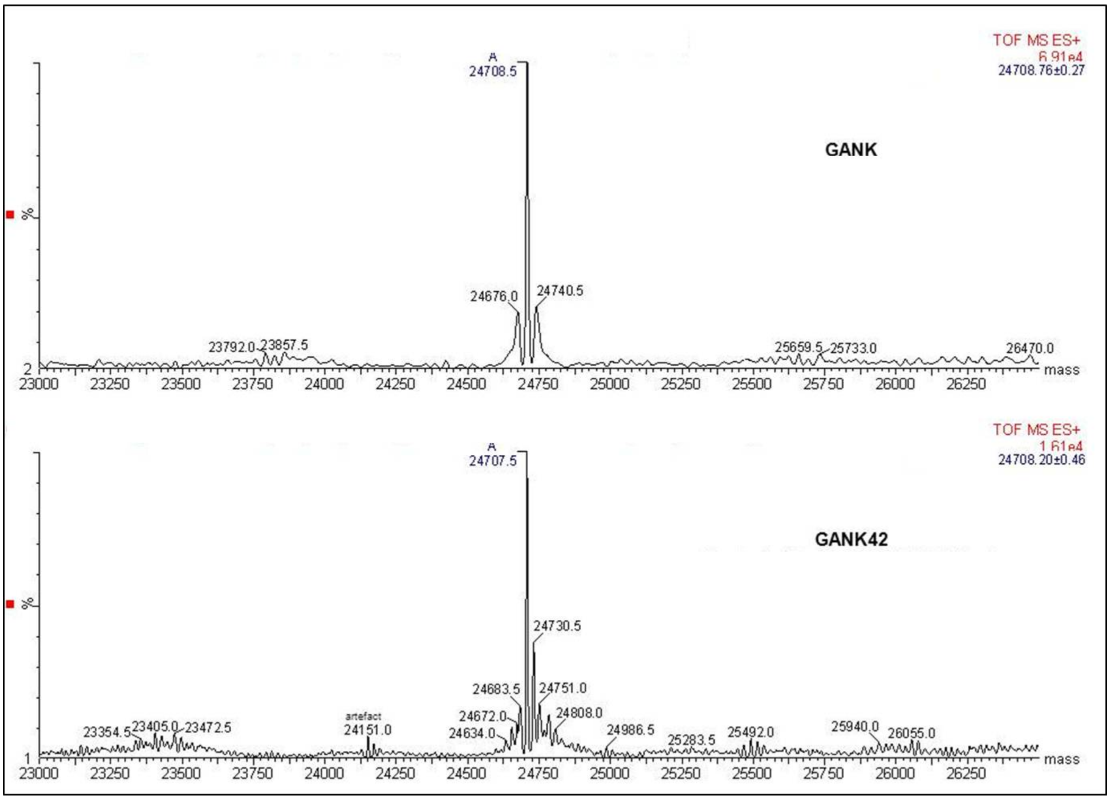

Supplementary Figure S12. Mass spectrometry data. TOF MS ES + mass spectra of of 50 mM gankyrin alone (top) and 50 mM gankyrin incubated with 300 mM cjoc042 (bottom). A a single major peak corresponding to the molecular weight of unconjugated gankyrin was observed in both cases, indicating that there was no covalent binding of cjoc42 to gankyrin.

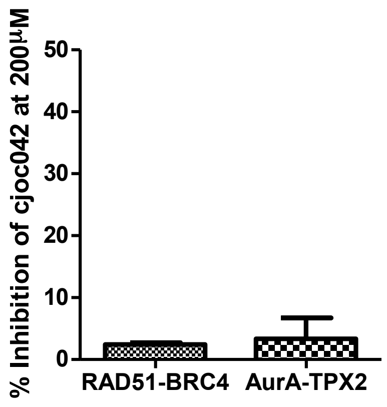

Supplementary Figure S13. Compound cjoc042 does not affect unrelated PPIs. cjoc42 behaves as a control molecule that does not show non-specific binding for two unrelated, well-characterized protein-protein interactions (RAD51-BRC4 and Aurora A-TPX2)

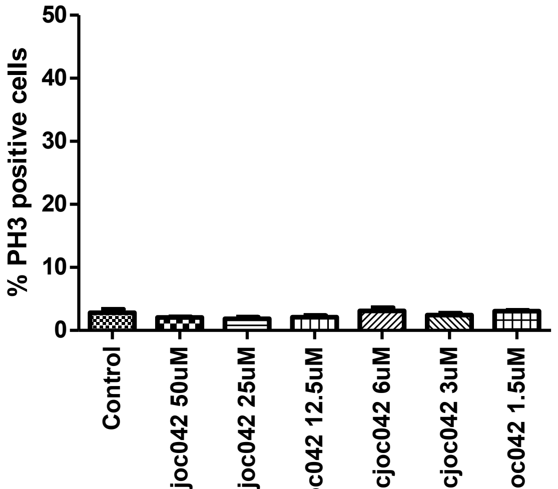

Supplementary Figure S14. Compound cjoc42 does not induce mitotic arrest. Compound cjoc42 at concentrations up to 50 μM does not induce mitotic arrest, as determined by the percentage of PH3-positive U2OS cells.

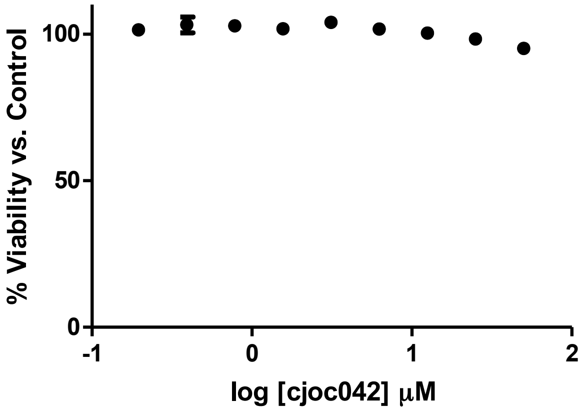

Supplementary Figure S15. Compound cjoc42 does not affect cell viability. Compound cjoc42 at concentrations up to 50 μM does not affect the proliferation of wild type U2OS cells in a 72 hr viability assay.

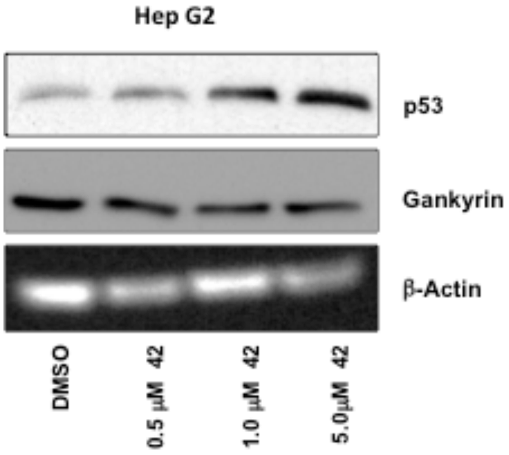

Supplementary Figure S16. Effects of cjoc42 addition on levels of p53 and β-actin in HepG2 cells that naturally over-express Gankyrin. After 48 hours in cells treated with cjoc42 (abbreviated to '42'), p53 expression levels show a dose dependant stabilisation compared to control cells.

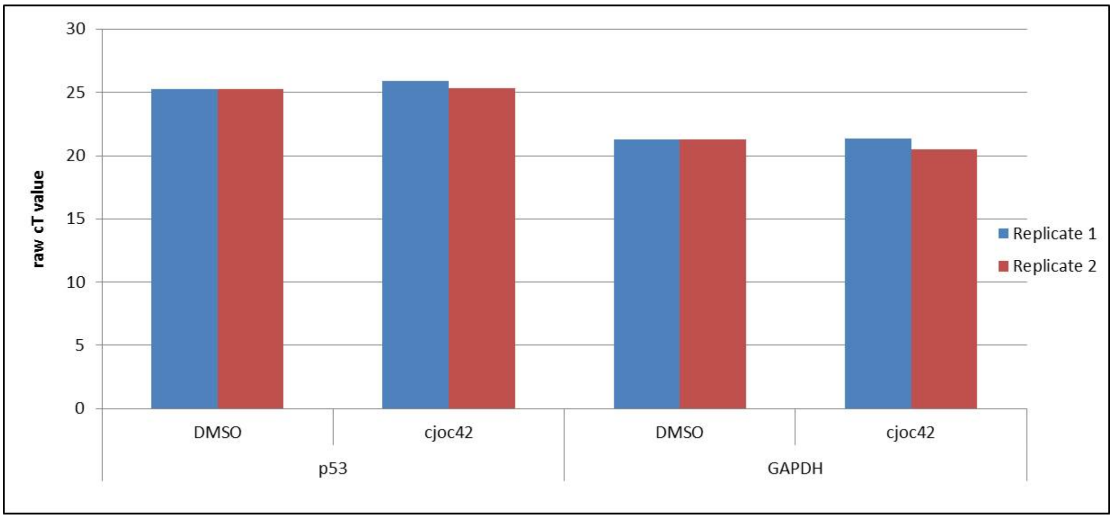

Supplementary Figure S17: qRT-PCR analysis of the effects of cjoc42 addition on p53 transcription in U2OS cells transfected to over-express gankyrin. GAPDH transcription was used as a housekeeping control. After 48 hours, cells treated with cjoc42 show no change in amplification threshold cycle (raw cT values) compared to control cells treated with DMSO, both for p53 and GAPDH. The ΔΔ cT ratio is 0.6, suggesting that cjoc42 does not alter the transcriptional profile of p53 in gankyrin overexpressing U2OS cells.

References

1. Nakamura, Y. *et al.* Structure of the oncoprotein gankyrin in complex with S6 ATPase of the 26S proteasome. *Structure* **15**, 179-189 (2007).

2. Robert, X. and Gouet, P. (2014) Deciphering key features in protein structures with the new ENDscript server. *Nucl. Acids Res*. **42**, W320-W324 (2014).

3. Nakamura, Y. *et al.* Structure of the oncoprotein gankyrin in complex with S6 ATPase of the 26S proteasome. *Structure* **15**, 179-189 (2007).

4. Supplementary Experimental Procedures

4.1. Chemical Synthesis: General details and equipment

Except as otherwise indicated, reactions were carried out using oven-dried glassware under nitrogen with dry, freshly distilled solvents. Tetrahydrofuran was distilled from calcium hydride and $LiAlH_4$ in the presence of triphenyl methane. Diethyl ether was distilled from calcium hydride and $LiAlH_4$. CH_2Cl_2, MeOH, toluene, MeCN and hexane were distilled from calcium hydride. Petrol ether refers to the 30-40 °C fractions. All other reagents were used as obtained from commercial sources. Room temperature (rt) refers to ambient temperature. Temperatures at 0 °C were maintained using an ice-water bath. Where possible, reactions were monitored by thin layer chromatography (TLC) using glass plates precoated with Merck silica gel 60 F_{254}. Visualization was by the quenching of UV fluorescence (λ_{max} = 254 nm) or by staining with potassium permanganate. Flash column chromatography was carried out using slurry-packed Merck 9385 Kieselgel 60 silica gel under a positive pressure of air or nitrogen. Infrared (IR) spectra were recorded on a Perkin-Elmer Spectrum One FT-IR spectrometer with internal referencing as neat films. Selected absorption maxima (ν_{max}) are reported in wavenumbers (cm^{-1}). Nuclear magnetic resonance (NMR) spectra were recorded using an internal deuterium lock on Bruker DPX 400 (400MHz), Bruker Avance 400 QNP Ultrashield (400 MHz), Bruker Avance 500 BB ATM (500 MHz) and Bruker Avance 500 Cryo Ultrashield (500 MHz) spectrometers. Chemical shifts (δ) are referenced to the solvent signal and are quoted in ppm to the nearest 0.01 ppm for ^{1}H NMR and to the nearest 0.1 ppm for ^{13}C NMR. Coupling constants (J) are reported in Hertz to the nearest 0.1 Hz. Assignments are supported by DEPT-135, ^{1}H-^{1}H COSY, HMQC, HMBC and NOESY spectra where necessary. Data are reported as follows: chemical shift, integration, multiplicity (app, apparent;

br, broad; s, singlet; d, doublet; t, triplet; q, quartet; quin, quintet; m, multiplet; or as a combination of these), coupling constant(s) and assignment. The numbering schemes used on selected spectra do not follow the IUPAC naming system and are used for the clear assignment of ^{1}H and ^{13}C spectra. High resolution mass spectrometry (HRMS) was carried out with a Micromass QTOF or a Waters LCT Premier Mass Spectrometer using electospray ionisation [ESI] or electron ionisation [EI]. The calculated mass value relative to found mass value is within the error limits of ±5 ppm mass units.

4.2. Chemical Synthseis: General Procedures

4.2.1. General Procedure 1

To a solution of amine (3.00 mmol) in HCl (1.0-1.5 eq) at 0 °C was added a solution of sodium nitrite (1.0-1.1 eq) in the dark. The reaction was stirred for 15 minutes and then a solution of sodium azide (1.05-1.1 eq) was added. The reaction was allowed to warm to room temperature and stirred overnight. The resulting precipitate was filtered and washed with H_2O (2 x 20 mL). The aqueous filtrate was acidified with dilute HCl and extracted with EtOAc (3 x 20 mL). The combined organic layers were washed with brine, dried over $MgSO_4$ and concentrated in vacuo.

4.2.2. General Procedure 2

To a stirred suspension of azido acid (6.00 mmol) in MeOH (20 mL) at room temperature were added carefully 2 drops of concentrated H_2SO_4. The reaction was heated at reflux and stirred overnight. Volatiles were removed at reduced pressure and the resulting residue was dissolved in EtOAc (100 mL), washed with saturate $NaHCO_3$ (2 x 50 mL) and brine. The organic layer was dried over $MgSO_4$ and

concentrated in vacuo. The resulting crude product was purified by column chromatography if required.

4.2.3. General Procedure 3

To a solution of alkyne (1.00 mmol) in *t*BuOH:THF (3:2; 6 mL) was added the appropriate azide (1.0 eq). The reaction was stirred at room temperature until the reagents dissolved and then H_2O (4 mL) was added. A solution of sodium ascorbate (1.0 M, 0.1-0.2 eq) and a solution of copper sulfate (0.3 M, 0.03-0.10 eq) were then added. The reaction was stirred at room temperature overnight. The reaction mixture was then partitioned between EtOAc (100 mL) and H_2O (50 mL). The layers were separated and the aqueous layer re-extracted with EtOAc (2 x 50 mL). The combined organic layers were washed with brine, dried over $MgSO_4$ and concentrated in vacuo. The resulting crude product was purified by column chromatography if required.

4.2.4. General Procedure 4

To a solution of tosyl chloride (1.5 eq) and the appropriate alcohol (2.00 mmol) in CH_2Cl_2 (5.00 mL) at 0 °C, were added treiethylamine (1 eq) and a DMAP (0.1 eq). The reaction was allowed to warm to room temperature and stirred overnight. The reaction mixture was partitioned between EtOAc (100 mL) and H_2O (50 mL). The layers were separated and the aqueous layer was re-extracted with EtOAc (2 x 50 mL). The combined organic layers were then washed with saturated ammonium chloride solution and brine before being dried over $MgSO_4$ and concentrated in vacuo. The resulting residue was purified by column chromatography.

4.2.5. General Procedure 5

To a solution of amino alkyne (6.00 mmol) in dichloromethane (15 mL) at 0 °C, were added the appropriate sulfonating agent (1.0-1.1 eq) and DIPEA (2.5 eq). The

reaction was allowed to warm to room temperature and stirred overnight. The reaction was partitioned between dichloromethane (100 mL) and water (50 mL). The aqueous layer was extracted with dichloromethane (2 x 50 mL) and the combined organic layers were washed with saturated ammonium chloride (100 mL) and brine (100 mL). The organics were dried over $MgSO_4$ and concentrated in vacuo. The crude product was purified by column chromatography if required.

4.3. Chemical Synthesis: Experimental procedures and characterization data

4-Azidobenzoic acid

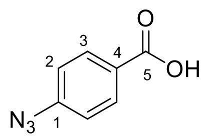

Prepared according to General Procedure 1 using 4-aminobenzoic acid (2.00 g, 14.58 mmol), sodium nitrite (1.0 M, 16.00 mL, 16.00 mmol), HCl (2.0 M, 8.00 mL, 16.00 mmol) and sodium azide (1.0 M, 15.30 mL, 15.30 mmol). The title compound was isolated as a yellow solid (2.12 g, 13.00 mmol, 89%).

IR (ν_{max} cm^{-1}): 2095, 1669, 1599, 1576, 1423, 1282, 1118, 932. **^{1}H NMR** (400 MHz, DMSO-d_6): δ = 12.95 (1H, br. s, OH), 7.96 (1H, d, *J*=8.9 Hz, H3) 7.22 (1H, d, *J*=8.9 Hz, H2) ppm. **^{13}C NMR** (100 MHz, DMSO-d_6) δ = 166.6 (C5), 144.0 (C1), 131.3 (C3), 127.4 (C4), 119.3 (C2) ppm. **HRMS** (ESI+): *m/z* found $[M+Na]^+$ 186.0274, $C_7H_5N_3O_2Na^+$ required 186.0277.

3-Azidobenzoic acid

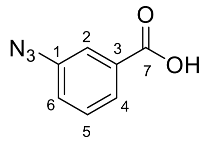

Prepared according to General Procedure 1 using 3-aminobenzoic acid (2.00 g, 14.58 mmol), sodium nitrite (1.0 M, 16.00 mL, 16.00 mmol), HCl (2.0 M, 8.00 mL, 16.00 mmol) and sodium azide (1.0 M, 15.30 mL, 15.30 mmol). The title compound was isolated as a yellow solid (1.95 g, 12.00 mmol, 82%).

IR (ν_{max} cm^{-1}): 2129, 1680, 1580, 1452, 1304, 1261. **^{1}H NMR** (500 MHz, DMSO-d_6): δ = 13.23 (1H, br. s, -OH), 7.74 (1H, dt, *J*=7.6, 1.4 Hz, H4), 7.56-7.57 (1H, m, H2), 7.53 (1H, app. t, *J*=7.6 Hz, H5), 7.34-7.38 (1H, m, H6) ppm. **^{13}C NMR** (100 MHz, DMSO-d_6): δ = 166.5 (C7), 140.0 (C1), 132.6 (C3), 130.4 (C5), 125.9 (C4), 123.6 (C6), 119.5 (C2) ppm. **HRMS** (ESI+): *m/*z found $[M+Na]^+$ 186.0274, $C_7H_5N_3O_2Na^+$ required 186.0277.

2-(3-Azidophenyl)acetic acid

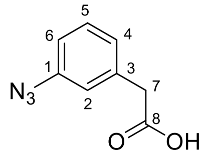

Prepared according to General Procedure 1 using 2-(3-aminophenyl)acetic acid (1.00 g, 6.62 mmol), sodium nitrite (1.0 M, 7.30 mL, 7.30 mmol), HCl (2.0 M, 3.50 mL, 7.00 mmol) and sodium azide (1.0 M, 7.00 mL, 7.00 mmol). The title compound was isolated as a yellow oil (985 mg, 5.56 mmol, 84%).

IR (ν_{max} cm^{-1}): 2113, 1694, 1584, 1446, 1403, 1288, 944. **^{1}H NMR** (400 MHz, DMSO-d_6): δ =12.54 (1H, br. s, -OH), 7.34 (1H, t, *J*=7.6 Hz, H5), 7.08 (1H, dd, *J*=8.3, 0.9 Hz, H4), 6.96-7.04 (2H, m, H2 & H6), 3.58 (2H, s, H7) ppm. **^{13}C NMR** (100 MHz, DMSO-d_6): δ = 172.6 (C8), 139.2 (C1), 137.5 (C3), 129.9 (C5), 126.5 (C4), 120.1 (C2), 117.4 (C6), 40.6 (C7) ppm. **HRMS** (ESI+): *m/z* found $[M+Na]^+$ 200.0430, $C_8H_7N_3O_2Na^+$ required 200.0431.

3-(3-Azidophenyl)propanoic acid

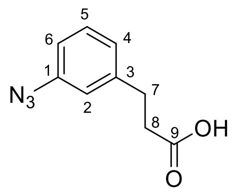

Prepared according to General Procedure 1 using 3-(3-aminophenyl)propanoic acid (2.00 g, 12.10 mmol), sodium nitrite (1.0 M, 13.30 mL, 13.30 mmol), HCl (2.0 M, 8.00 mL, 16.00 mmol) and sodium azide (1.0 M, 12.70 mL, 12.70 mmol). The title compound was isolated as a yellow oil (1.94 g, 10.16 mmol, 84%).

IR (ν_{max} cm^{-1}): 2114, 1694, 1606, 1434, 1414, 1289, 1212, 934. **^{1}H NMR** (500 MHz, DMSO-d_6): δ = 12.18 (1H, br. s, -OH), 7.34 (1H, t, *J*=7.8 Hz, H5), 7.06 (1H, d, *J*=7.8 Hz, H4), 7.01 (1H, s, H2), 6.93-6.98 (1H, m, H6), 2.84 (2H, t, *J*=7.5 Hz, H8), 2.56 (2H, t, *J*=7.5 Hz, H7) ppm. **^{13}C NMR** (100 MHz, DMSO-d_6): δ = 174.1 (C9), 143.6 (C1), 139.7 (C3), 130.3 (C5), 125.7 (C4), 119.3 (C2), 117.2 (C6), 35.4 (C8), 30.5 (C7) ppm.

Methyl 4-azidobenzoate

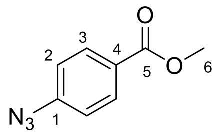

Prepared according to General Procedure 2 using 4-azidobenzoic acid (1.00 g, 6.13 mmol). The desired ester was isolated as a yellow solid (1.02 g, 5.76 mmol, 94%).

IR (ν_{max} cm^{-1}): 2116, 1717, 1600, 1504, 1431, 1272, 1171, 1103. **^{1}H NMR** (400 MHz, $CDCl_3$): δ = 8.03 (2H, d, *J*=8.7 Hz, H3) 7.06 (2H, d, *J*=8.7 Hz, H2) 3.91 (3H, s, H6) ppm. **^{13}C NMR** (100 MHz, $CDCl_3$): δ = 166.3 (C5), 144.8 (C1), 131.4 (C3), 126.7 (C4), 118.8 (C2), 52.2 (C6) ppm. **HRMS** (EI+): *m/z* found $[M+Na]^+$ 200.0430, $C_8H_7N_3O_2Na^+$ required 200.0439.

Methyl 3-azidobenzoate

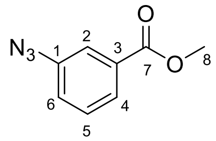

Prepared according to General Procedure 2 using 3-azidobenzoic acid (1.00 g, 6.13 mmol). The title compound was isolated as a yellow solid (855 mg, 4.47 mmol, 73%).

IR (ν_{max} cm^{-1}): 2107, 1721, 1585, 1439, 1293, 1253, 1138, 1081, 984. **^{1}H NMR** (500 MHz, DMSO-d_6): δ = 7.68-7.71 (1H, m, H4), 7.49 (1H, t, *J*=7.6 Hz, H5), 7.46-7.48 (1H, m, H2), 7.30-7.33 (1H, ddd, *J*=7.9, 2.4, 1.2 Hz, H6), 3.83 (3H, s, H8) ppm. **^{13}C NMR** (100 MHz, DMSO-d_6): δ = 165.4 (C7), 140.1 (C1), 131.3 (C3), 130.4 (C5), 125.7 (C4), 123.8 (C6), 119.2 (C2), 52.4 (C8) ppm. **HRMS** (ESI+): *m/z* found $[M+Na]^+$ 200.0430, $C_8H_7N_3O_2Na^+$ required 200.0439.

Methyl 2-(3-azidophenyl)acetate

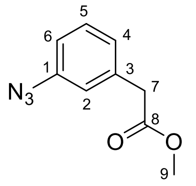

Prepared according to General Procedure 2 using 2-(3-aminophenyl)acetic acid (1.00 g, 5.64 mmol). The desired ester was isolated as a yellow solid (912 mg, 4.77 mmol, 85%).

IR (ν_{max} cm^{-1}): 2107, 1753, 1605, 1588, 1487, 1435, 1287, 1256, 1157, 1012. **^{1}H NMR** (400 MHz, DMSO-d_6): δ = 7.36 (1H, t, J=7.8 Hz, H5), 7.09 (1H, d, J=7.8 Hz, H4), 6.99-7.05 (2H, m, H2 & H6), 3.72 (2H, s, H7), 3.62 (3H, s, H9) ppm. **^{13}C NMR** (100 MHz, DMSO-d_6): δ = 171.8 (C8), 139.8 (C1), 137.0 (C3), 130.4 (C5), 126.8 (C4), 120.5 (C2), 118.1 (C6), 52.2 (C9), 40.1 (C7) ppm. **HRMS** (ESI+)**:** *m/z* found $[M+Na]^+$ 214.0587, $C_9H_9N_3O_2Na^+$ required 214.0597.

Methyl 3-(3-azidophenyl)propanoate

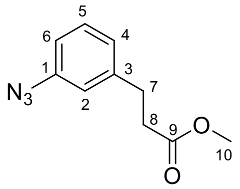

Prepared according to General Procedure 2 using 3-(3-azidophenyl)propanoic acid (1.20 g, 6.30 mmol). The title compound was isolated as a yellow solid (1.10 g, 5.36 mmol, 85%).

IR (ν_{max} cm^{-1}): 2107, 1734, 1606, 1587, 1487, 1436, 1285, 1196, 1163, 875. ^{1}**H NMR** (400 MHz, DMSO-d_6): δ = 7.32 (1H, t, *J*=7.8 Hz, H5), 7.05 (1H, d, *J*=7.2 Hz, H4), 7.00-6.98 (1H, m, H2), 6.94 (1H, dd, *J*=7.8, 1.7 Hz, H6), 3.58 (3H, s, H10), 2.85 (2H, t, *J*=7.5 Hz, H8), 2.64 (2H, t, *J*=7.5 Hz, H7) ppm. ^{13}**C NMR** (100 MHz, DMSO-d_6): δ = 172.6 (C9), 142.9 (C1), 139.4 (C3), 130.0 (C5), 125.3 (C4), 119.0 (C2), 117.0 C6), 51.4 (C10), 34.6 (C7), 30.0 (C8) ppm. **HRMS** (ESI+): *m/z* found $[M+Na]^+$ 228.0743, $C_{10}H_{11}N_3O_2Na^+$ required 228.0748.

Methyl 4-(4-(3-hydroxypropyl)-1*H*-1,2,3-triazol-1-yl)benzoate

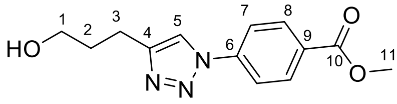

Prepared according to General Procedure 3 using methyl 4-azidobenzoate (1.00 g, 5.64 mmol), 4-pentynol (525 µL, 5.64 mmol), copper sulfate (0.3 M, 560 µL, 0.17 mmol) and sodium ascorbate (1.0 M, 560 µL, 0.56 mmol). The crude product was purified by column chromatography eluting with EtOAc. The title compound was isolated as a yellow oil (980 mg, 3.75 mmol, 66%).

IR (ν_{max} cm^{-1}): 3231, 3131, 2951, 1719, 1607, 1435, 1281, 1112, 1043. **^{1}H NMR** (500 MHz, DMSO-d_6): δ = 8.71 (1H, s, H5), 8.15 (2H, d, *J*=8.8 Hz, H8), 8.08 (2H, d, *J*=8.8 Hz, H7), 4.53 (1 H, t, *J*=5.2 Hz, -OH), 3.89 (3H, s, H11), 3.45-3.51 (2H, m, H1), 2.74-2.78 (2H, m, H3), 1.79-1.86 (2H, m, H2) ppm. **^{13}C NMR** (100 MHz, DMSO-d_6) δ = 165.5 (C10), 148.7 (C4), 140.1 (C6), 131.1 (C8), 129.1 (C9), 120.4 (C5), 119.7 (C7), 60.1 (C1), 52.5 (C11), 32.1 (C2), 21.8 (C3) ppm. **HRMS** (ESI+): *m/z* found [M+H]$^+$ 262.1181, $C_{11}H_{16}N_3O_3^+$ required 262.1192.

Methyl 3-(4-(3-hydroxypropyl)-1*H*-1,2,3-triazol-1-yl)benzoate

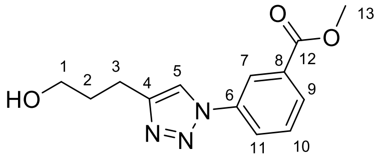

Prepared according to general procedure 3 using methyl 3-azidobenzoate (196 mg, 1.11 mmol), 4-pentynol (103 μL, 1.11 mmol), copper sulfate (0.3 M, 110 μL, 0.03 mmol) and sodium ascorbate (1.0 M, 110 μL, 0.11 mmol). The crude product was purified by column chromatography eluting with EtOAc. The title compound was isolated as a colourless oil which solidified slowly (180 mg, 0.70 mmol, 63%).

IR (ν_{max} cm^{-1}): 3356, 3143, 2950, 1712, 1594, 1483, 1283, 1055. **^{1}H NMR** (400 MHz, DMSO-d_6): δ = 8.72 (1H, s, H5), 8.42 (1H, s, H7), 8.18 (1H, dd, *J*=8.0, 0.9 Hz, H11), 8.03 (1H, d, *J*=7.8 Hz, H9), 7.75 (1H, t, *J*=7.8 Hz, H10), 4.52 (1H, t, *J*=5.1 Hz, H-OH), 3.92 (3H, s, H13), 3.49 (2H, app. q, *J*=6.0 Hz, H1), 2.75 (2H, t, *J*=7.7 Hz, H3), 1.76-1.90 (2H, m, H2) ppm. **^{13}C NMR** (100 MHz, DMSO-d_6): δ = 165.4 (C12) 148.4 (C4), 137.1 (C6), 131.3 (C8), 130.7 (C10), 128.8 (C9), 124.3 (C11), 120.4 (C5), 120.0 (C7), 60.1 (C1), 52.7 (C13), 32.1 (C2), 21.8 (C3) ppm. **HRMS** (ESI+): *m/z* found [M+H]$^+$ 262.1192, $C_{11}H_{16}N_3O_3^+$ required 262.1192.

Methyl 2-(3-(4-(3-hydroxypropyl)-1*H*-1,2,3-triazol-1-yl)phenyl)acetate

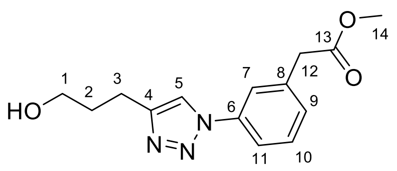

Prepared according to general procedure 3 using methyl 2-(3-azidophenyl)acetate (705 mg, 3.70 mmol), 4-pentynol (343 μL, 3.70 mmol), copper sulfate (0.3 M, 400 μL, 0.11 mmol) and sodium ascorbate (1.0 M, 400 μL, 0.40 mmol). The crude product was purified by column chromatography eluting with EtOAc. The title compound was isolated as a yellow oil (642 mg, 2.33 mmol, 63%).

IR (ν_{max} cm^{-1}): 3386, 2951, 1730, 1612, 1593, 1437, 1199, 1164, 1044. ^{1}**H NMR** (500 MHz, $CDCl_3$): δ = 7.79 (1H, s, H5), 7.67 (1H, t, *J*=1.7 Hz, H7), 7.62 (1H, ddd, *J*=7.9, 2.2, 1.1 Hz, H9), 7.47 (1H, t, *J*=7.9 Hz, H10), 7.33-7.36 (1H, m, H11), 3.75 (2H, t, *J*=6.1 Hz, H1), 3.71 (5H, s, H12 & H14), 2.93 (2H, t, *J*=7.3 Hz, H3), 1.97-2.04 (2H, m, H2) ppm. ^{13}**C NMR** (125 MHz, $CDCl_3$): δ = 171.3 (C13), 148.1 (C4), 137.2 (C6), 135.9 (C8), 129.9 (C10), 129.6 (C11), 121.4 (C7), 119.4 (C5), 119.2 (C9), 61.7 (C1), 52.5 (C12), 40.8 (C14), 31.9 (C2), 22.0 (C3) ppm. **HRMS** (ESI+): *m/z* found $[M+H]^+$ 276.1334, $C_{14}H_{18}O_3N_3^+$ required 276.1343.

Methyl 3-(3-(4-(3-hydroxypropyl)-1*H*-1,2,3-triazol-1-yl)phenyl)propanoate

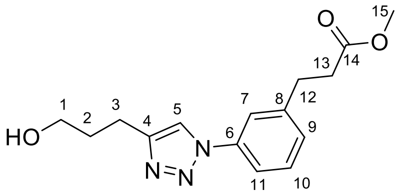

Prepared according to general procedure 3 using methyl 3-(3-azidophenyl)propanoate (710 mg, 3.46 mmol), 4-pentynol (321 μL, 3.46 mmol), copper sulfate (0.3 M, 400 μL, 0.11 mmol) and sodium ascorbate (1.0 M, 400 μL, 0.40 mmol). The crude product was purified by column chromatography eluting with EtOAc. The title compound was isolated as a yellow oil (746 mg, 2.58 mmol, 75%).

IR (ν_{max} cm^{-1}): 3345, 2952, 1732, 1612, 1595, 1435, 1228, 1159, 1045, 1012. **^{1}H NMR** (500 MHz, $CDCl_3$): δ = 7.78 (1H, s, H5), 7.59 (1H, t, *J*=1.7 Hz, H7), 7.53 (1H, ddd, *J*=8.1, 2.1, 1.1 Hz, H9), 7.41 (1 H, t, *J*=7.8 Hz, H10), 7.24-7.27 (1H, m, H11), 3.75 (2H, t, *J*=6.1 Hz, H1), 3.66 (3H, s, H15), 3.02 (2H, t, *J*=7.6 Hz, 13), 2.92 (2 H, t, *J*=7.3 Hz, H3), 2.68 (2H, t, *J*=7.8 Hz, H12), 2.00 (2 H, tt, *J*=7.3, 6.1 Hz, H2) ppm. **^{13}C NMR** (125 MHz, $CDCl_3$): δ = 172.9 (C14), 148.1 (C4), 142.6 (C6), 137.2 (C8), 129.8 (C10), 128.6 (C11), 120.4 (C7), 119.1 (C5), 118.3 (C9), 61.7 (C1), 51.7 (C15), 35.2 (C12), 31.9 (C2), 30.7 (C13), 22.0 (C3) ppm. **HRMS** (ESI+): *m/z* found $[M+H]^+$ 290.1501, $C_{15}H_{20}N_3O_3^+$ required 290.1505.

Methyl 4-(4-(3-(tosyloxy)propyl)-1*H*-1,2,3-triazol-1-yl)benzoate (cjoc42)

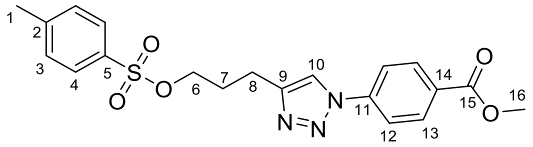

Prepared as per general procedure 4 using methyl 4-(4-(3-hydroxypropyl)-1*H*-1,2,3-triazol-1-yl)benzoate (500 mg, 1.91 mmol), tosyl chloride (550 mg, 2.87 mmol), triethylamine (270 μL, 1.91 mmol) and DMAP (24 mg, 0.20 mmol). The crude product was purified by column chromatography eluting in gradient from 30% EtOAc in petrol ether (30-40) to 50% EtOAc in petrol ether (30-40). The title compound **(cjoc42)** was isolated as a white solid (650 mg, 1.57 mmol, 82%).

IR (ν_{max} cm^{-1}): 2957, 1723, 1610, 1438, 1351, 1272, 1172, 1100, 1042, 962. **^{1}H NMR** (400 MHz, $CDCl_3$): δ = 8.19 (2H, d, *J*=7.8 Hz, H13), 7.73-7.87 (5H, m, H4, H10 & H12), 7.33 (2H, d, *J*=7.8 Hz, H3), 4.10 (2H, t, *J*=6.1 Hz, H6), 3.96 (3H, s, H16), 2.89 (2H, t, *J*=7.2 Hz, H8), 2.42 (3H, s, H1), 2.09-2.20 (2H, m, H7) ppm. **^{13}C NMR** (100 MHz, DMSO-d_6): δ = 165.9 (C15), 147.1 (C9), 144.9 (C2), 140.1 (C11), 132.9 (C5), 131.4 (C13), 130.1 (C14), 129.1 (C3), 127.9 (C4), 119.8 (C12), 119.4 (C10), 69.3 (C6), 52.5 (C16), 28.2 (C7), 21.6 (C8), 21.4 (C1) ppm. **HRMS** (ESI+): *m/z* found $[M+H]^+$ 416.1270, $C_{20}H_{22}O_5N_3S^+$ required416.1275.

Methyl 3-(4-(3-(tosyloxy)propyl)-1*H*-1,2,3-triazol-1-yl)benzoate (1)

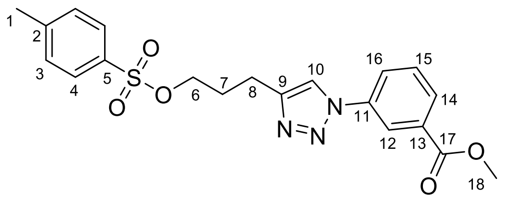

Prepared according to general procedure 4 using methyl 3-(4-(3-hydroxypropyl)-1*H*-1,2,3-triazol-1-yl)benzoate (50 mg, 0.19 mmol), tosyl chloride (55 mg, 0.29 mmol), triethylamine (27 μL, 0.19 mmol) and DMAP (2 mg, 0.02 mmol). The crude product was purified by column chromatography eluting with 30% EtOAc in petrol ether (30-40). The title compound **(1)** was isolated as a colourless oil (65 mg, 0.16 mmol, 85%).

IR (ν_{max} cm^{-1}): 2953, 1724, 1594, 1354, 1273, 1174, 926. **^{1}H NMR** (500 MHz, DMSO-d_6): δ = 8.66 (1H, s, H10), 8.38 (1H, t, *J*=1.7 Hz, H12), 8.12-8.16 (1H, m, H14), 8.01-8.06 (1H, m, H16), 7.73-7.80 (3H, m, H4 & H15), 7.41-7.45 (2H, m, H3), 4.11 (2H, t, *J*=6.3 Hz, H6), 3.92 (3H, s, H18), 2.72 (2H, t, *J*=7.5 Hz, H8), 2.35 (3H, s, H1), 2.01 (2H, s, H7) ppm. **^{13}C NMR** (126 MHz, DMSO-d_6): δ = 165.4 (C17), 146.8 (C9), 144.8 (C2), 136.9 (C2), 132.3 (C5), 131.26 (C13), 130.6 (C15), 130.1 (C3), 128.8 (C16), 127.6 (C4), 124.2 (C14), 120.6 (C10), 119.9 (C12), 70.0 (C6), 52.6 (C18), 27.6 (C7), 21.0 (C1), 20.9 (C8) ppm. **HRMS** (ESI+): *m/z* found [M+H]$^+$ 416.1281, $C_{20}H_{22}N_3O_5S^+$ required 416.1280.

Methyl 3-(3-(4-(3-(tosyloxy)propyl)-1*H*-1,2,3-triazol-1-yl)phenyl)propanoate (2)

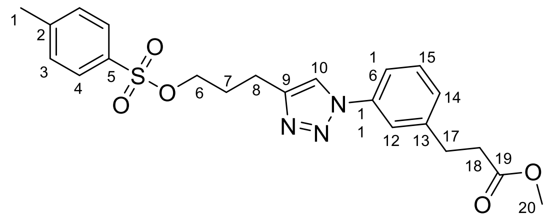

Prepared according to general procedure 4 using methyl 3-(3-(4-(3-hydroxypropyl)-1*H*-1,2,3-triazol-1-yl)phenyl)propanoate (710 mg, 2.45 mmol), tosyl chloride (700 mg, 3.68 mmol), triethylamine (340 µL, 2.45 mmol) and DMAP (30 mg, 0.25 mmol). The crude product was purified by column chromatography eluting with 30% EtOAc in petrol ether (30-40). The title compound **(2)** was isolated as a colourless oil (777 mg, 1.75 mmol, 72%).

IR (ν_{max} cm^{-1}): 3059, 2952, 1730, 1614, 1495, 1357, 1173, 1120, 1033, 1010, 817. **^{1}H NMR** (400 MHz, $CDCl_3$): δ = 7.78 (2H, d, *J*=8.5 Hz, H4), 7.71 (1H, s, H10), 7.57-7.61 (1H, m, H12), 7.48-7.54 (1H, m, H16), 7.43 (1H, t, *J*=7.8 Hz, H15), 7.33 (2H, d, *J*=8.2 Hz, H3), 7.26-7.29 (1H, m, H14), 4.10 (2H, t, *J*=6.1 Hz, H6), 3.68 (3H, s, H20), 3.04 (2H, t, *J*=7.7 Hz, H17), 2.87 (2H, t, *J*=7.2 Hz, H8), 2.70 (2H, t, *J*=7.7 Hz, H8), 2.42 (3H, s, H1), 2.09-2.17 (2H, m, H7) ppm. **^{13}C NMR** (100 MHz, $CDCl_3$): δ = 172.9 (C20), 146.7 (C9), 144.8 (C2), 142.6 (C11), 137.2 (C13), 132.9 (C5), 129.87 (C3), 129.83 (C15), 128.6 (C14), 127.9 (C4), 120.5 (C12), 119.6 (C10), 118.3 (C16), 69.4 (C6), 51.8 (C20), 35.3 (18), 30.7 (C17), 28.3 (C7), 21.6 (C1), 21.4 (C8) ppm. **HRMS:** (*m/z*-ESI) calcd. for $C_{22}H_{26}N_3O_5S$ [M+H] 444.1593, found 444.1596.

Methyl 2-(3-(4-(3-(tosyloxy)propyl)-1*H*-1,2,3-triazol-1-yl)phenyl)acetate (3)

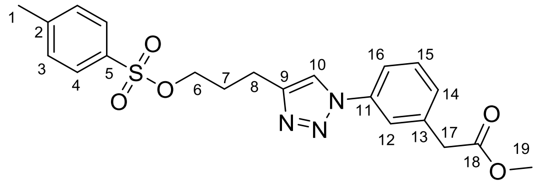

Prepared according to general procedure 4 using methyl 2-(3-(4-(3-hydroxypropyl)-1*H*-1,2,3-triazol-1-yl)phenyl)acetate (642 mg, 2.33 mmol), tosyl chloride (670 mg, 3.50 mmol), triethylamine (320 μL, 2.33 mmol) and DMAP (30 mg, 0.25 mmol). The crude product was purified by column chromatography eluting with 30% EtOAc in petrol ether (30-40). The title compound **(3)** was isolated as a colourless oil (586 mg, 1.36 mmol, 59%).

IR (ν_{max} cm^{-1}): 3058, 2952, 1733, 1436, 1348, 1190, 1172, 1119, 1032, 1011. **^{1}H NMR** (400 MHz, $CDCl_3$): δ = 7.78 (2H, d, *J*=8.2 Hz, H4), 7.72 (1H, s, H10), 7.64-7.68 (1H, m, 12), 7.57-7.62 (1H, m, H16), 7.47 (1H, t, *J*=7.8 Hz, H15), 7.29-7.38 (3H, m, H14 & H3), 4.10 (2H, t, *J*=6.0 Hz, H6), 3.72 (5H, s, H17 & H19), 2.87 (2H, t, *J*=7.3 Hz, H8), 2.41 (3H, s, H1), 2.08-2.17 (2H, m, H7) ppm. **^{13}C NMR** (100 MHz, $CDCl_3$): δ = 171.3 (C18), 146.7 (C9), 144.9 (C2), 137.3 (C11), 135.9 (C13), 132.9 (C5), 129.90 (C15), 129.87 (C3), 129.5 (C14), 127.9 (C4), 121.4 (C12), 119.6 (C10), 119.1 (C16), 69.3 (C6), 52.3 (C19), 40.8 (C17), 28.3 (C7), 21.6 (C8), 21.4 (C1) ppm. **HRMS** (ESI+): *m/z* found $[M+H]^+$ 430.1452, $C_{21}H_{24}N_3O_5S^+$ required 430.1437.

4-Methyl-*N*-(pent-4-yn-1-yl)benzenesulfonamide

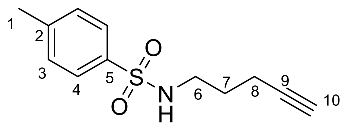

Prepared according to general procedure 5 using 4-pentynamine (500 mg, 6.00 mmol), tosyl chloride (1.20 g, 6.30 mmol) and DIPEA (2.60 mL, 15.00 mmol). The crude product was purified by column chromatography eluting in gradient from 10% EtOAc in hexane to 33% EtOAc in hexane. The title compound was isolated as a yellow solid (885 mg, 3.73, 62%).

IR (ν_{max} cm^{-1}): 3274, 2930, 1318, 1301, 1153, 1088, 1077, 947. **^{1}H NMR** (500 MHz, $CDCl_3$): δ = 7.75 (2H, d, *J*=8.1 Hz, H4), 7.28-7.33 (2H, d, *J*=8.1 Hz, H3), 4.73 (1H, t, *J*=6.1 Hz, -NH), 3.07 (2H, q, *J*=6.7 Hz, H6), 2.43 (3H, s, H1), 2.22 (2H, td, *J*=6.9, 2.6 Hz, H8), 1.94 (1H, t, *J*=2.7 Hz, H11), 1.68 (2H, quin, *J*=6.8 Hz, H7) ppm. **^{13}C NMR** (125 MHz, $CDCl_3$): δ = 143.5 (C2), 136.9 (C5), 129.7 (C3), 127.1 (C4), 82.8 (C9), 69.5 (C11), 42.1 (C6), 28.1 (C7), 21.5 (C1), 15.7 (C8) ppm. **HRMS** (ESI+): *m/z* found $[M+H]^+$ 238.0899, $C_{12}H_{16}NO_2S^+$ required 238.0902.

4-Methyl-*N*-(prop-2-yn-1-yl)benzenesulfonamide

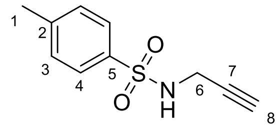

Prepared according to general procedure 5 using propargyl amine (1.30 mL, 20.30 mmol), tosyl chloride (3.87 g, 20.30 mmol) and DIPEA (8.70 mL, 50.00 mmol). The title compound was isolated as a white solid (4.10 g, 19.59 mmol, 97%).

IR (ν_{max} cm^{-1}): 3268, 1320, 1157, 1092, 1067, 869. **^{1}H NMR** (500 MHz, $CDCl_3$): δ = 7.77 (2 H, d, *J*=8.2 Hz, H4), 7.30-7.34 (2H, m, H3), 4.57-4.60 (1H, m, -NH), 3.83 (2H, dd, *J*=6.1, 2.4 Hz, H6), 2.43 (3H, s, H1), 2.11 (1 H, t, *J*=2.4 Hz, H8) ppm. **^{13}C NMR** (125 MHz, $CDCl_3$): δ = 143.9 (C2), 136.5 (C5), 129.7 (C3), 127.4 (C4), 77.9 (C7), 73.0 (C8), 32.9 (C6), 21.6 (C1) ppm. **HRMS** (ESI+): *m/z* found $[M+H]^+$ 210.0597, $C_{10}H_{12}NO_2S^+$ required 210.0589.

***N*-(Pent-4-yn-1-yl)naphthalene-2-sulfonamide**

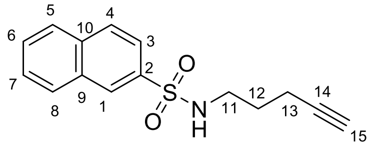

Prepared according to general procedure 5 using 4-pentynamine (500 mg, 6.00 mmol), 2-naphthylsulfonyl chloride (1.36 g, 6.00 mmol) and DIPEA (2.60 mL, 15.00 mmol). The title compound was isolated as a colourless oil which solidifies upon standing (1.04 g, 3.80 mmol, 63%).

IR (ν_{max} cm^{-1}): 3297, 3261, 1309, 1152, 1128, 1060, 971. **^{1}H NMR** (400 MHz, $CDCl_3$): δ = 8.45 (1 H, d, *J*=1.7 Hz, H1), 7.95-8.00 (2H, m, H4 & H8), 7.89-7.94 (1H, m, H5), 7.85 (1H, dd, *J*=8.9, 1.7 Hz, H3), 7.59-7.68 (2H, m, H6 & H7), 4.82

(1H, br. s, -NH), 3.13 (2H, q, *J*=6.6 Hz, H11), 2.22 (2H, td, *J*=6.8, 2.7 Hz, H13), 1.91 (1H, t, *J*=2.7 Hz, H15), 1.70 (2H, quin, *J*=6.8 Hz, H12) ppm. **^{13}C NMR** (100 MHz, $CDCl_3$): δ = 136.6 (C2), 134.8 (C10), 132.1 (C9), 129.6 (C4), 129.2 (C8), 128.8 (C1), 128.5 (C6), 127.9 (C5), 127.6 (C7), 122.2 (C3), 82.8 (C14), 69.5 (C15), 42.2 (C11), 28.1 (C13), 15.7 (C12) ppm. **HRMS** (ESI)+: *m/z* found $[M+H]^+$ 274.0900, $C_{15}H_{16}NO_2S^+$ required 274.0902.

***N*-(Prop-2-yn-1-yl)naphthalene-2-sulfonamide**

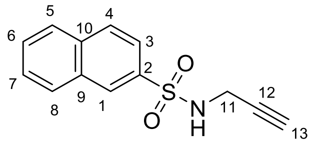

Prepared according to general procedure 5 using propargyl amine (1.30 mL, 20.30 mmol), 2-naphthalenesulfonyl chloride (4.60 g, 20.30 mmol) and DIPEA (8.70 mL, 50.00 mmol). The title compound was isolated as a white solid (3.80 g, 15.49 mmol, 76%).

IR (ν_{max} cm^{-1}): 3262, 1321, 1156, 1128, 1058, 880. **^{1}H NMR** (500 MHz, $CDCl_3$): δ = 8.48 (1H, d, *J*=1.8 Hz, H1), 7.95-8.00 (2H, m, H4 & H8), 7.92 (1H, dd, *J*=7.2, 0.8 Hz, H5), 7.86 (1H, dd, *J*=8.5, 2.1 Hz, H3), 7.59-7.69 (2H, m, H6 & H7), 4.74 (1H, br. s, -NH), 3.89 (2H, dd, *J*=6.0, 2.6 Hz, H11), 2.02 (1H, t, *J*=2.6 Hz, H13) ppm. **^{13}C NMR** (125 MHz, $CDCl_3$): δ = 136.3 (C2), 135.0 (C10), 132.1 (C9), 129.5 (C4), 129.3 (C8), 128.94 (C1), 128.92 (C6), 127.9 (C5), 127.6 (C7), 122.4 (C3), 77.9 (C12), 73.1 (C11), 33.0 (C13) ppm. **HRMS** (ESI+): *m/z* found $[M+H]^+$ 246.0588, $C_{13}H_{12}NO_2S^+$ required 246.0589.

4-Azido-*N*-propylbenzamide

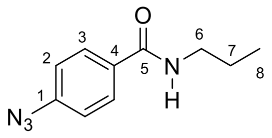

To a solution of 4-azidobenzoic acid (200 mg, 1.23 mmol) and propylamine (100 μL, 1.23 mmol) in EtOAc (10 mL) at 0 °C, were added T3P (50% in EtOAc, 0.95 mL, 1.60 mmol) and DIPEA (430 μL, 2.46 mmol). The reaction was allowed to warm to room temperature and stirred overnight. The reaction mixture was partitioned between EtOAc (30 mL) and water (30 mL). The aqueous layer was extracted with EtOAc (2 x 30 mL) and the combined organic layers were washed with saturated bicarbonate solution (50 mL), brine (100 mL) and dried over $MgSO_4$. Volatiles were removed at reduced pressure providing the title compound as a yellow oil (187 mg, 0.92 mmol, 75%).

IR (ν_{max} cm^{-1}): 3308, 2964, 2117, 1628, 1602, 1548, 1499, 1283, 1248, 1151. **^{1}H NMR** (500 MHz, $CDCl_3$): δ = 7.76 (2H, d, *J*=8.9 Hz, H3), 7.05 (2H, d, *J*=8.9 Hz, H2), 6.14 (1 H, br. s, -NH), 3.39-3.44 (2H, m, H6), 1.64 (2H, sxt, *J*=7.3 Hz, H7), 0.98 (3H, t, *J*=7.5 Hz, H8) ppm. **^{13}C NMR** (125 MHz, $CDCl_3$): δ = 166.5 (C5), 143.1 (C6), 131.3 (C9), 128.6 (C3), 119.0 (C2), 41.8 (C6), 22.9 (C7), 11.4 (C8) ppm. **HRMS** (ESI+): *m/z* found $[M+H]^+$ 205.1093, $C_{10}H_{13}N_4O^+$ required 205.1089.

(*S*)-methyl 2-(4-azidobenzamido)propanoate

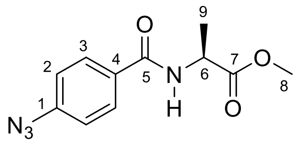

To a solution of 4-azidobenzoic acid (200 mg, 1.23 mmol) and L-alanine methyl ester hydrochloride (172 mg, 1.23 mmol) in EtOAc (10 mL) at 0 °C, were added T3P (50% in EtOAc, 0.95 mL, 1.60 mmol) and DIPEA (860 µL, 4.92 mmol). The reaction was allowed to warm to room temperature and stirred overnight. The reaction mixture was partitioned between EtOAc (30 mL) and water (30 mL). The aqueous layer was extracted with EtOAc (2 x 30 mL) and the combined organic layers were washed with saturated bicarbonate solution (50 mL), brine (100 mL) and dried over $MgSO_4$. Volatiles were removed at reduced pressure providing the title compound as a yellow oil (253 mg, 1.02 mmol, 83%).

IR (ν_{max} cm^{-1}): 3323, 2133, 2086, 1730, 1636, 1604, 1532, 1500, 1288, 1264, 1045, 972, 849. **^{1}H NMR** (500 MHz, $CDCl_3$): δ = 7.81 (2H, d, *J*=8.9 Hz, H3), 7.07 (2H, d, *J*=8.9 Hz, H2), 6.71 (1H, d, *J*=6.4 Hz, -NH), 4.79 (1H, quin, *J*=7.2 Hz, H6), 3.79 (3H, s, H8), 1.52 (3H, d, *J*=7.0 Hz, H9) ppm. **^{13}C NMR** (125 MHz, $CDCl_3$): δ = 173.7 (C7), 165.7 (C5), 143.6 (C1), 130.3 (C4), 128.8 (C3), 119.0 (C2), 52.6 (C8), 48.5 (C6), 18.7 (C9) ppm. **HRMS** (ESI+): *m/z* found $[M+H]^+$ 249.0988, $C_{11}H_{13}N_4O_3$ required 249.0988.

Methyl 4-(4-(3-(4-methylphenylsulfonamido)propyl)-1*H*-1,2,3-triazol-1-yl)benzoate (4)

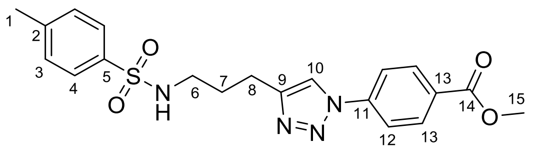

Prepared according to general procedure 3 using 4-methyl-*N*-(pent-4-yn-1-yl)benzenesulfonamide (200 mg, 0.84 mmol), methyl 4-azidobenzoate (149 mg, 0.84 mmol), copper sulfate (0.3 M, 84 μL, 25 μmol) and sodium ascorbate (1.0 M, 84 μL, 84 μmol). The title compound **(4)** was isolated as a yellow solid (300 mg, 0.72 mmol, 86%).

IR (ν_{max} cm^{-1}): 3277, 1699, 1440, 1334, 1290, 1156, 1109, 1029, 989. **^{1}H NMR** (500 MHz, $CDCl_3$): δ = 8.19 (2H, d, *J*=8.8 Hz, H13), 7.89 (1H, br. s, H10), 7.83 (2H, d, *J*=8.8 Hz, H12), 7.73 (2H, d, *J*=8.3 Hz, H4), 7.27-7.31 (2H, m, H3), 3.96 (3H, s, H16), 3.02 (2H, t, *J*=6.5 Hz, H6), 2.88 (2H, t, *J*=7.0 Hz, H8), 2.40 (3H, s, H1), 1.95 (2H, quin, *J*=6.7 Hz, H7) ppm. **^{13}C NMR** (125 MHz, $CDCl_3$): δ = 165.9 (C15), 147.5 (C9), 143.5 (C5), 140.1 (C11), 136.8 (C2), 131.3 (C13), 130.0 (C14), 129.7 (C3), 127.0 (C4), 119.7 (C12), 119.5 (C10), 52.4 (C16), 42.1 (C6), 28.8 (C7), 22.1 (C8), 21.5 (C1) ppm. **HRMS** (ESI+): *m/z* found $[M+H]^+$ 415.1448, $C_{20}H_{23}N_4O_4S^+$ required 415.1440.

Methyl 4-(4-(3-(*N*,4-dimethylphenylsulfonamido)propyl)-1*H*-1,2,3-triazol-1-yl)benzoate (5)

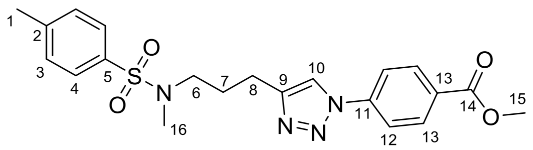

To a suspension of methyl 4-(4-(3-(4-methylphenylsulfonamido)propyl)-1*H*-1,2,3-triazol-1-yl)benzoate (**4**, 200 mg, 0.45 mmol) and Cs_2CO_3 (236 mg, 0.72 mmol) in DMF (5 mL) was added methyl iodide (33 μL, 0.53 mmol). The reaction was stirred at room temperature overnight. The reaction was partitioned between EtOAc (50 mL) and water (30 mL). The aqueous layer was extracted with EtOAc (2 x 50 mL) and the combined organic layers were washed with water (2 x 30 mL) and brine (50 mL) before being dried over $MgSO_4$ and concentrated in vacuo. The title compound **(5)** was isolated as a colourless oil (164 mg, 0.38 mmol, 80%).

IR (ν_{max} cm^{-1}): 1715, 1609, 1441, 1330, 1283, 1229, 1151, 1110, 1006, 925. ^{1}**H NMR** (500 MHz, $CDCl_3$): δ = 8.20 (2H, d, *J*=8.8 Hz, H13), 8.04 (1H, s, H10), 7.87 (2H, d, *J*=8.8 Hz, H12), 7.64 (2H, d, *J*=8.0 Hz, H4), 7.31 (2H, d, *J*=8.0 Hz, H3), 3.96 (3H, s, H16), 3.04 (2H, t, *J*=6.6 Hz, H6), 2.92 (2H, t, *J*=7.0 Hz, H8), 2.71 (3H, s, H17), 2.42 (3H, s, H1), 2.01 (2H, quin, *J*=6.7 Hz, H7) ppm. ^{13}**C NMR** (125 MHz, $CDCl_3$): δ = 166.0 (C15), 147.7 (C9), 143.4 (C5), 140.3 (C11), 134.0 (C2), 131.3 (C13), 129.9 (C14), 129.7 (C3), 127.4 (C4), 120.0 (C10), 119.8 (C12), 52.4 (C16), 48.8 (C6), 34.6 (C17), 26.6 (C7), 21.9 (C8), 21.5 (C1). **HRMS** (ESI+): *m/z* found $[M+H]^+$ 429.1613, $C_{21}H_{25}N_4O_4S^+$ required 429.1597.

4-(4-(3-(4-Methylphenylsulfonamido)propyl)-1*H*-1,2,3-triazol-1-yl)benzoic acid (6)

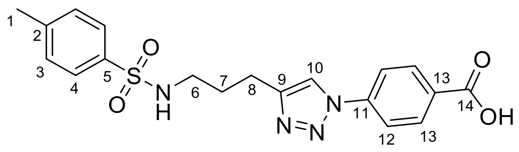

To a solution of methyl 4-(4-(3-(4-methylphenylsulfonamido)propyl)-1*H*-1,2,3-triazol-1-yl)benzoate (**4**, 55 mg, 0.13 mmol) in THF (0.5 mL) was added a solution of lithium hydroxide (8 mg, 0.20 mmol) in EtOH-H_2O (2.0 mL, 3:1). The reaction was stirred at room temperature until the starting material was fully consumed as monitored by TLC. The reaction was acidified with dilute HCl to pH 2, before being partitioned between EtOAc (20 mL) and water (10 mL). The aqueous layer was extracted with EtOAc (2 x 20 mL) and the combined organic layers were washed with water (20 mL) and brine (30 mL) before being dried over $MgSO_4$ and concentrated in vacuo. The title compound **(6)** was isolated as a white solid (48 mg, 0.12 mmol, 92%).

IR (ν_{max} cm^{-1}): 3254, 1685, 1607, 1441, 1322, 1294, 1154, 1067, 909, 860. ^{1}**H NMR** (500 MHz, DMSO-d_6) δ = 8.62 (1H, s, H10), 8.12 (1H, d, *J*=8.8 Hz, H13), 8.01 (1H, d, *J*=8.8 Hz, H12), 7.66 (1H, d, *J*=8.3 Hz, H4), 7.62 (1H, t, *J*=5.7 Hz, -NH), 7.37 (1H, d, *J*=8.0 Hz, H3), 2.79 (2H, dd, *J*=13.0, 6.8 Hz, H6), 2.69 (2H, t, *J*=7.7 Hz, H8), 2.35 (3H, s, H1), 1.75-1.82 (2H, m, H7) ppm. ^{13}**C NMR** (125 MHz, DMSO-d_6) δ = 166.6 (C15), 147.8 (C9), 142.7 (C5), 139.8 (C11), 137.7 (C2), 131.2 (C13), 130.4 (C14), 129.8 (C3), 126.6 (C4), 120.5 (C10), 119.6 (C12), 40.0 (C6), 28.8 (C7), 22.2 (C8), 21.1 (C1) ppm. **HRMS** (ESI+): *m/z* found $[M+H]^+$ 401.1302, $C_{19}H_{21}N_4O_4S^+$ required 401.1284.

4-(4-(3-(*N*,4-Dimethylphenylsulfonamido)propyl)-1*H*-1,2,3-triazol-1-yl)benzoic acid (7)

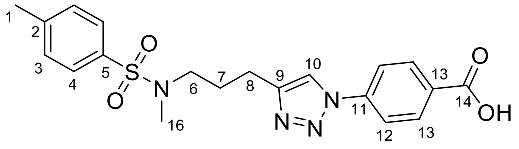

To a solution of methyl 4-(4-(3-(*N*,4-dimethylphenylsulfonamido)propyl)-1*H*-1,2,3-triazol-1-yl)benzoate (**5**, 92 mg, 0.21 mmol) in THF (1.0 mL) was added a solution of lithium hydroxide (14 mg, 0.32 mmol) in EtOH-H_2O (4.0 mL, 3:1). The reaction was stirred at room temperature until the starting material was fully consumed as monitored by TLC. The reaction was acidified with dilute HCl to pH 2, before being partitioned between EtOAc (20 mL) and water (10 mL). The aqueous layer was extracted with EtOAc (2 x 20 mL) and the combined organic layers were washed with water (20 mL) and brine (30 mL) before being dried over $MgSO_4$ and concentrated in vacuo. The title compound **(7)** was isolated as a white solid (79 mg, 0.19 mmol, 90%).

IR (ν_{max} cm^{-1}): 2921, 1682, 1607, 1425, 1332, 1287, 1156, 1040, 965. **^{1}H NMR** (500 MHz, $CDCl_3$): δ = 8.28 (2H, d, *J*=9.1 Hz, H13), 8.08 (1H, s, H10), 7.92 (2H, d, *J*=8.8 Hz, H12), 7.64 (2H, d, *J*=8.3 Hz, H4), 7.31 (2H, d, *J*=8.0 Hz, H3), 3.04 (2H, t, *J*=6.5 Hz, H6), 2.94 (2H, t, *J*=7.0 Hz, H8), 2.71 (3H, s, H16), 2.42 (3H, s, H16), 2.02 (3H, dt, *J*=13.2, 6.4 Hz, H7) ppm. **^{13}C NMR** (125 MHz, $CDCl_3$): δ = 169.4 (C15), 147.7 (C9), 143.5 (C5), 140.8 (C11), 133.9 (C2), 132.0 (C13), 129.7 (C3), 129.0 (C14), 127.4 (C4), 120.1 (C10), 119.9 (C12), 48.8 (C6), 34.5 (C16), 26.5 (C7), 21.9 (C8),

21.5 (C1) ppm. **HRMS** (ESI+): *m/z* found $[M+H]^+$ 415.1453, $C_{20}H_{23}N_4O_4S^+$ required 415.1440.

4-(4-(3-(4-Methylphenylsulfonamido)propyl)-1*H*-1,2,3-triazol-1-yl)-*N*-propylbenzamide (8)

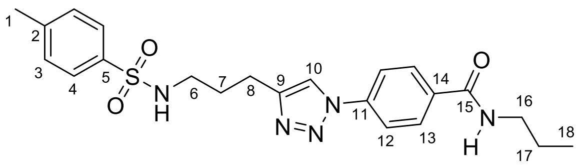

Prepared according to general procedure 3 using 4-methyl-*N*-(pent-4-yn-1-yl)benzenesulfonamide (60 mg, 0.25 mmol), 4-azido-*N*-propylbenzamide (51 mg, 0.25 mmol), copper sulfate (0.3 M, 50 μL, 15 μmol) and sodium ascorbate (1.0 M, 50 μL, 50 μmol). The title compound **(8)** was isolated as a yellow solid (105 mg, 0.24 mmol, 95%).

IR (ν_{max} cm^{-1}): 3367, 3156, 2968, 1629, 1608, 1542, 1509, 1322, 1303, 1160, 1147, 1052, 985. **^{1}H NMR** (500 MHz, DMSO-d_6): δ = 8.59 (1H, s, H10), 8.04 (2H, d, *J*=9.2 Hz, H13), 7.96 (2H, d, *J*=8.9 Hz, H12), 7.67 (2H, d, *J*=8.2 Hz, H4), 7.61 (1H, t, *J*=5.8 Hz, -NH), 7.37 (2H, dd, *J*=8.5, 0.6 Hz, H3), 3.22-3.27 (2H, m, H16), 2.78-2.83 (2H, m, H6), 2.69 (2H, t, *J*=7.5 Hz, H8), 2.35 (3H, s, H1), 1.75-1.82 (2H, m, H7), 1.50-1.60 (2H, m, H17), 0.90 (3H, t, *J*=7.5 Hz, H18) ppm. **^{13}C NMR** (125 MHz, DMSO-d_6): δ = 165.1 (C15), 147.6 (C9), 142.6 (C2), 138.4 (C11), 137.7 (C5), 134.3 (C14), 129.7 (C3), 128.9 (C13), 126.6 (C4), 120.4 (C10), 119.3 (C12), 42.0 (C6), 41.2 (C16), 28.8 (C7), 22.4 (C17), 22.2 (C8), 21.0 (C1), 11.6 (C18) ppm. **HRMS** (ESI+): *m/z* found $[M+H]^+$ 442.1929, $C_{22}H_{27}N_5O_3S^+$ required 442.1913.

(*S*)-methyl 2-(4-(4-(3-(4-methylphenylsulfonamido)propyl)-1*H*-1,2,3-triazol-1-yl)benzamido)propanoate (9)

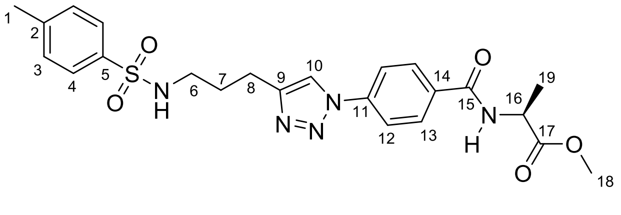

Prepared according to general procedure 3 using 4-methyl-*N*-(pent-4-yn-1-yl)benzenesulfonamide (60 mg, 0.25 mmol), (*S*)-methyl 2-(4-azidobenzamido)propanoate (62 mg, 0.25 mmol), copper sulfate (0.3 M, 50 µL, 15 µmol) and sodium ascorbate (1.0 M, 50 µL, 50 µmol). The title compound **(9)** was isolated as a yellow solid (110 mg, 0.23 mmol, 91%).

IR (ν_{max} cm^{-1}): 3295, 2927, 1750, 1734, 1650, 1506, 1324, 1157, 1043. **^{1}H NMR** (500 MHz, DMSO-d_6): δ = 8.95 (1 H, d, *J*=7.0 Hz, -NH), 8.61 (1H, s, H10), 8.09 (2H, d, *J*=8.9 Hz, H13), 8.00 (2H, d, *J*=8.9 Hz, H12), 7.67 (2H, d, *J*=8.2 Hz, H4), 7.61 (1H, t, *J*=5.8 Hz, -NH), 7.38 (2H, d, *J*=7.9 Hz, H3), 4.51 (1H, quin, *J*=7.2 Hz, H16), 3.66 (3H, s, H18), 2.81 (2H, q, *J*=6.8 Hz, H6), 2.70 (2H, t, *J*=7.6 Hz, H8), 2.36 (3H, s, H1), 1.79 (2H, quin, *J*=7.3 Hz, H7), 1.43 (3H, d, *J*=7.3 Hz, H19) ppm. **^{13}C NMR** (125 MHz, DMSO-d_6): δ = 173.2 (C17), 165.2 (C15), 147.7 (C9), 142.6 (C2), 138.8 (C11), 137.7 (C5), 133.2 (C14), 129.7 (C3), 129.3 (C13), 126.6 (C4), 120.4 (C10), 119.3 (C12), 52.0 (C18), 48.5 (C16), 42.0 (C6), 28.8 (C7), 22.2 (C8), 21.0 (C1), 16.8 (C19). **HRMS** (ESI+): *m/z* found [M+H]$^+$ 486.1809, $C_{23}H_{28}N_5O_5S^+$ required 486.1811.

Methyl 4-(4-(3-(naphthalene-2-sulfonamido)propyl)-1*H*-1,2,3-triazol-1-yl)benzoate (10)

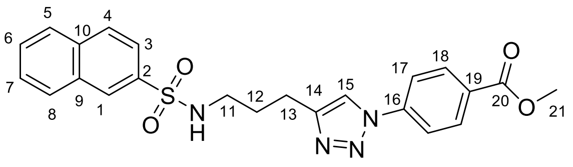

Prepared according to general procedure 3 using *N*-(pent-4-yn-1-yl)naphthalene-2-sulfonamide (770 mg, 2.82 mmol), methyl 4-azidobenzoate (500 mg, 2.82 mmol), copper sulfate (0.3 M, 282 μL, 85 μmol) and sodium ascorbate (282 μL, 0.28 mmol). The crude product was triturated in Et_2O and the title compound **(10)** was isolated by filtration as a yellow solid (1.16 g, 2.57 mmol, 91%).

IR (ν_{max} cm^{-1}): 3252, 1720, 1608, 1440, 1313, 1281, 1151, 1043. **^{1}H NMR** (500 MHz, DMSO-d_6): δ = 8.59 (1H, s, H15), 8.41-8.44 (1H, m, H1), 8.10-8.16 (4H, m, H8, H18 & H4), 7.97-8.04 (3H, m, H17 & H5), 7.78-7.85 (2H, m, H3 & -NH), 7.61-7.71 (2H, m, H7 & H6), 3.89 (3H, s, H21), 2.88 (2H, q, *J*=6.6 Hz, H11), 2.70 (2H, t, *J*=7.5 Hz, H13), 1.76-1.84 (2H, m, H12) ppm. **^{13}C NMR** (125 MHz, DMSO-d_6): δ = 165.4 (C20), 147.8 (C14), 139.9 (C16), 137.6 (C2), 134.2 (C10), 131.8 (C9), 131.0 (C18), 129.5 (C8), 129.2 (C4), 129.1 (C19), 128.7 (C7), 127.9 (C5), 127.6 (C6), 127.4 (C1), 122. **HRMS** (ESI+): *m/z* found $[M+H]^+$ 451.1440, $C_{23}H_{23}N_4O_4S^+$ required 451.1440.

Methyl 4-(4-((4-methylphenylsulfonamido)methyl)-1*H*-1,2,3-triazol-1-yl)benzoate (11)

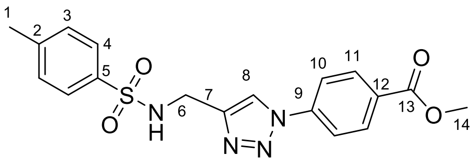

Prepared according to general procedure 3 using 4-methyl-*N*-(prop-2-yn-1-yl)benzenesulfonamide (590 mg, 2.82 mmol), methyl 4-azidobenzoate (500 mg, 2.82 mmol), copper sulfate (0.3 M, 282 μL, 85 μmol) and sodium ascorbate (1.0 M, 282 μL, 0.28 mmol). The crude product was triturated in Et_2O and the title compound was isolated by filtration as a yellow solid (847 mg, 2.19 mmol, 78%).

IR (ν_{max} cm^{-1}): 3285, 1702, 1608, 1445, 1435, 1335, 1293, 1156, 1069, 1040. **^{1}H NMR** (500 MHz, DMSO-d_6) δ = 8.55 (1H, s, H8), 8.11-8.21 (3H, m, -NH & H11), 7.99 (2H, d, *J*=8.9 Hz, H10), 7.66 (2H, d, *J*=7.9 Hz, H4), 7.32 (2H, d, *J*=7.9 Hz, H3), 4.14 (2H, d, *J*=5.8 Hz, H7), 3.90 (3H, s, H14), 2.27 (3H, s, H1) ppm. **^{13}C NMR** (125 MHz, DMSO-d_6): δ = 165.4 (C13), 144.8 (C7), 142.7 (C2), 139.7 (C9), 137.6 (C5), 131.1 (C11), 129.5 (C3), 129.4 (C12), 129.8 (C4), 121.8 (C8), 119.8 (C10), 52.5 (C14), 38.0 (C6), 20.9 (C1) ppm. **HRMS** (ESI+): *m/z* found $[M+H]^+$ 387.1132, $C_{18}H_{19}N_4O_4S^+$ required 387.1127.

Methyl 4-(4-((naphthalene-2-sulfonamido)methyl)-1*H*-1,2,3-triazol-1-yl)benzoate (12)

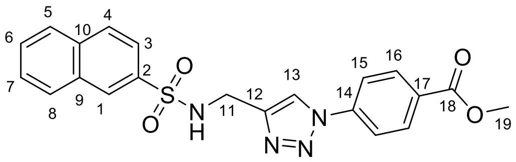

Prepared according to general procedure 3 using *N*-(prop-2-yn-1-yl)naphthalene-2-sulfonamide (692 mg, 2.82 mmol), methyl 4-azidobenzoate (500 mg, 2.82 mmol), copper sulfate (0.3 M, 282 μL, 85 μmol) and sodium ascorbate (1.0 M, 282 μL, 0.28 mmol). The crude product was triturated in Et_2O and the title compound **(12)** was isolated by filtration as a yellow solid (780 mg, 1.85 mmol, 65%).

IR (ν_{max} cm^{-1}): 3268, 1720, 1608, 1438, 1317, 1283, 1156, 1048. **^{1}H NMR** (400 MHz, DMSO-d_6): δ = 8.53 (1H, s, H13), 8.34-8.43 (2H, m, H1 & -NH)), 8.01-8.13 (4H, m, H8, H16 & H4), 7.92-7.98 (1H, m, H5), 7.76-7.82 (3H, m, H15 & H3), 7.58-7.68 (2H, m, H7 & H6), 4.22 (2H, d, *J*=5.8 Hz, H11), 3.89 (3H, s, H19) ppm. **^{13}C NMR** (125 MHz, DMSO-d_6) δ = 165.4 (C18), 144.6 (C12), 139.5 (C14), 137.5 (C2), 134.1 (C10), 131.6 (C9), 130.9 (C16), 129.3 (C17 & C8), 129.2 (C4), 128.7 (C6), 127.8 (C5), 127.6 (C7), 127.5 (C1), 122.4 (C3), 121.8 (C13), 119.7 (C15), 52.5 (C11), 38.0 (C19) ppm. **HRMS** (ESI+): *m/z* found $[M+H]^+$ 423.1125, $C_{21}H_{19}N_4O_4S^+$ required 423.1127.

4.4 Chemical Synthesis: ^{1}H and ^{13}C NMR spectra

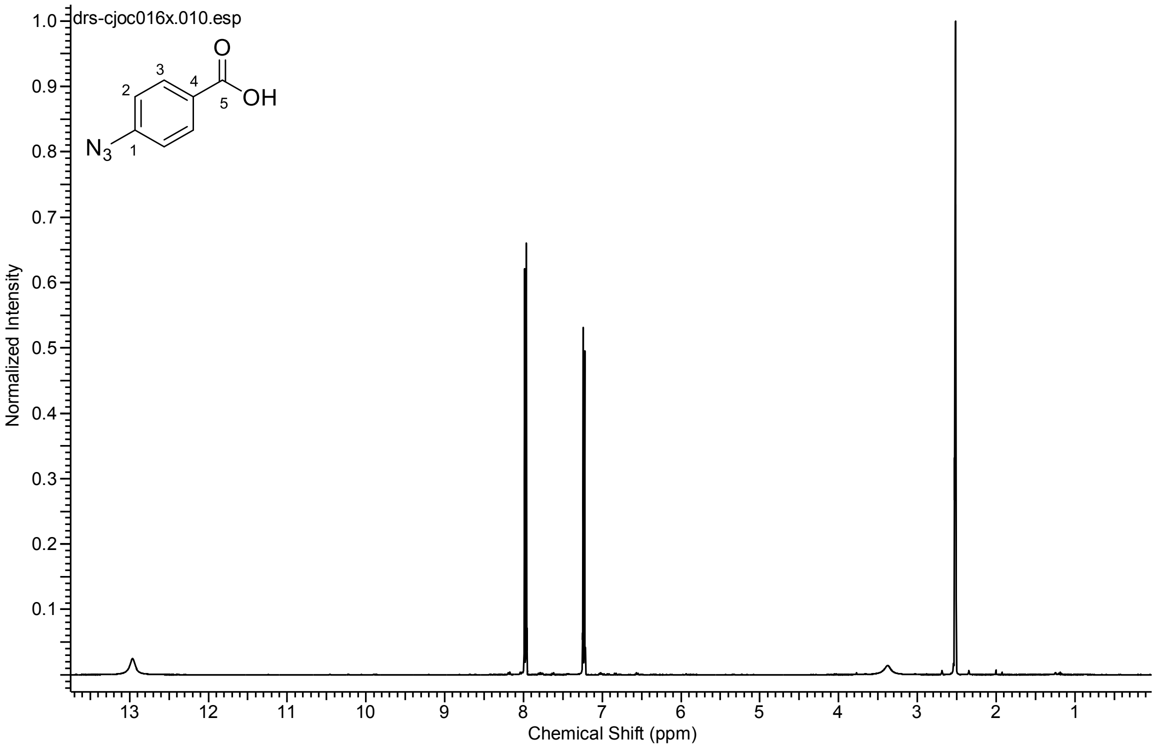

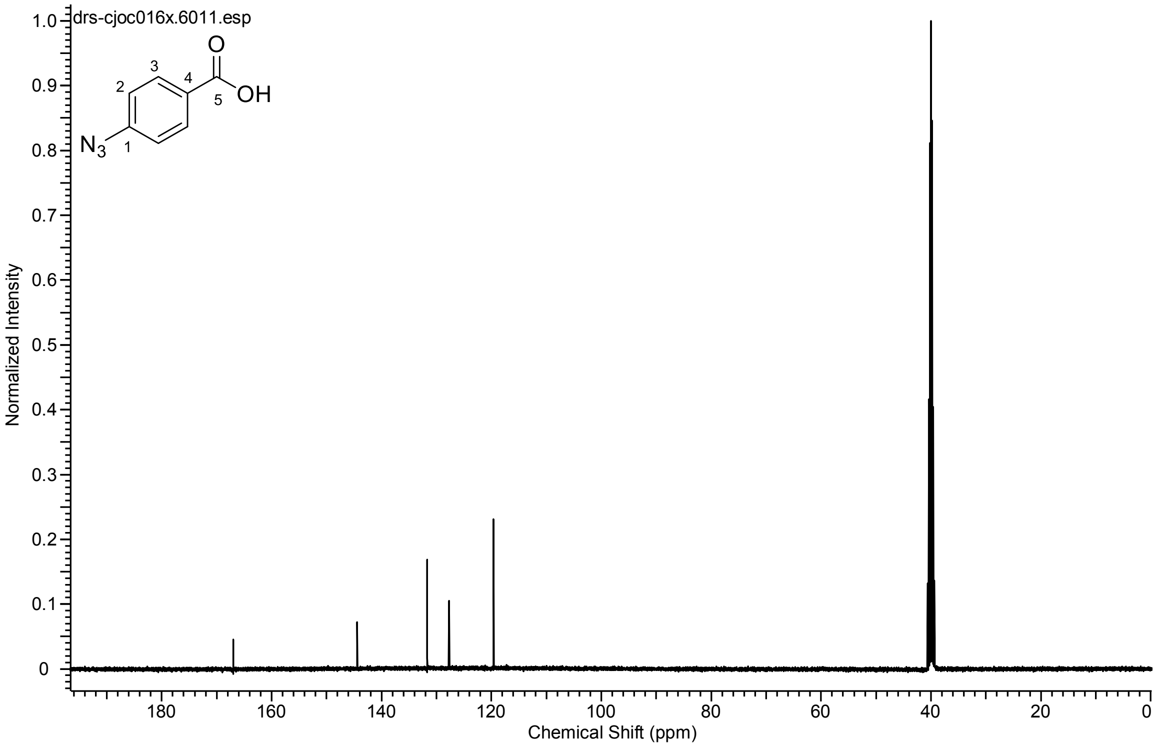

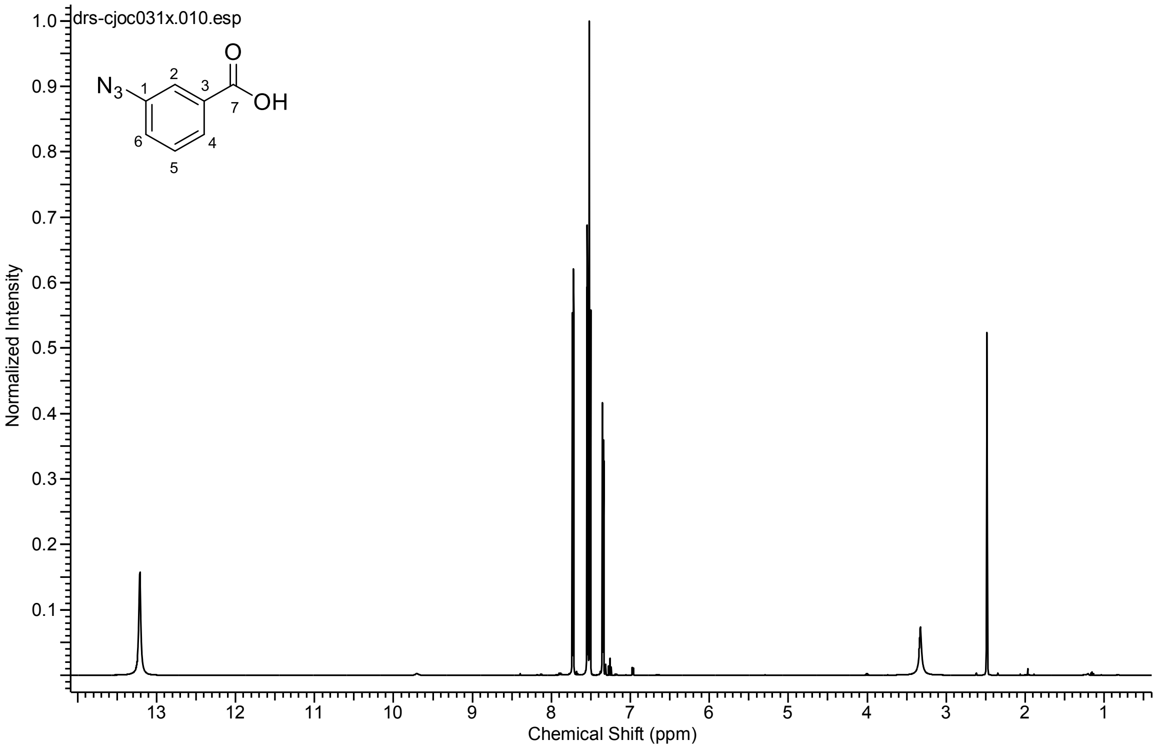
drs-cjoc031x.010.esp
Normalized Intensity
Chemical Shift (ppm)
N3
OH
O

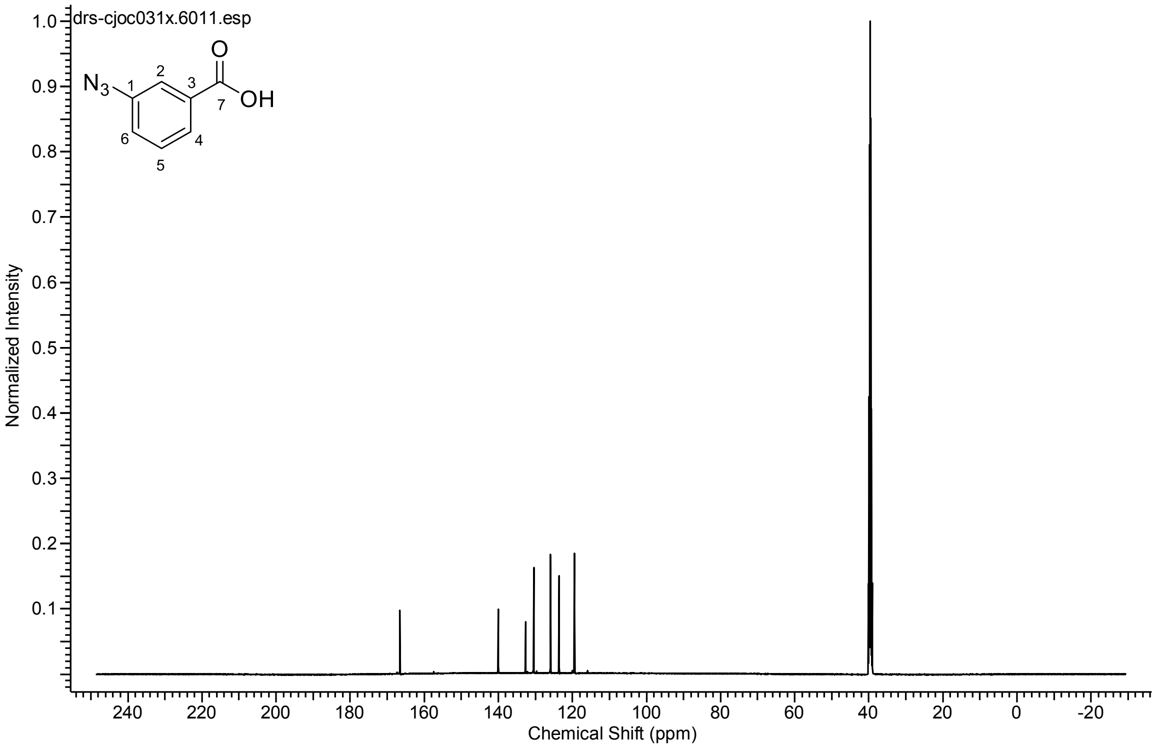
drs-cjoc031x.6011.esp
Normalized Intensity
Chemical Shift (ppm)
N3
OH
O

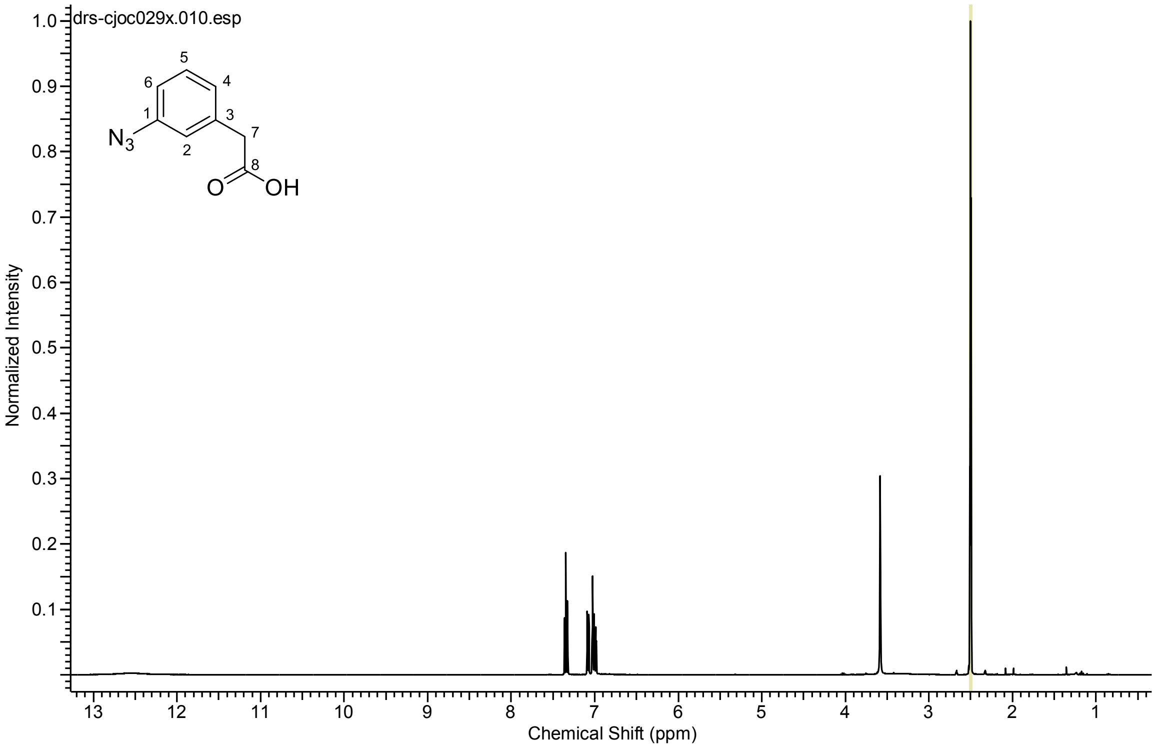
drs-cjoc029x.010.esp
Normalized Intensity
Chemical Shift (ppm)
N_3
OH
O

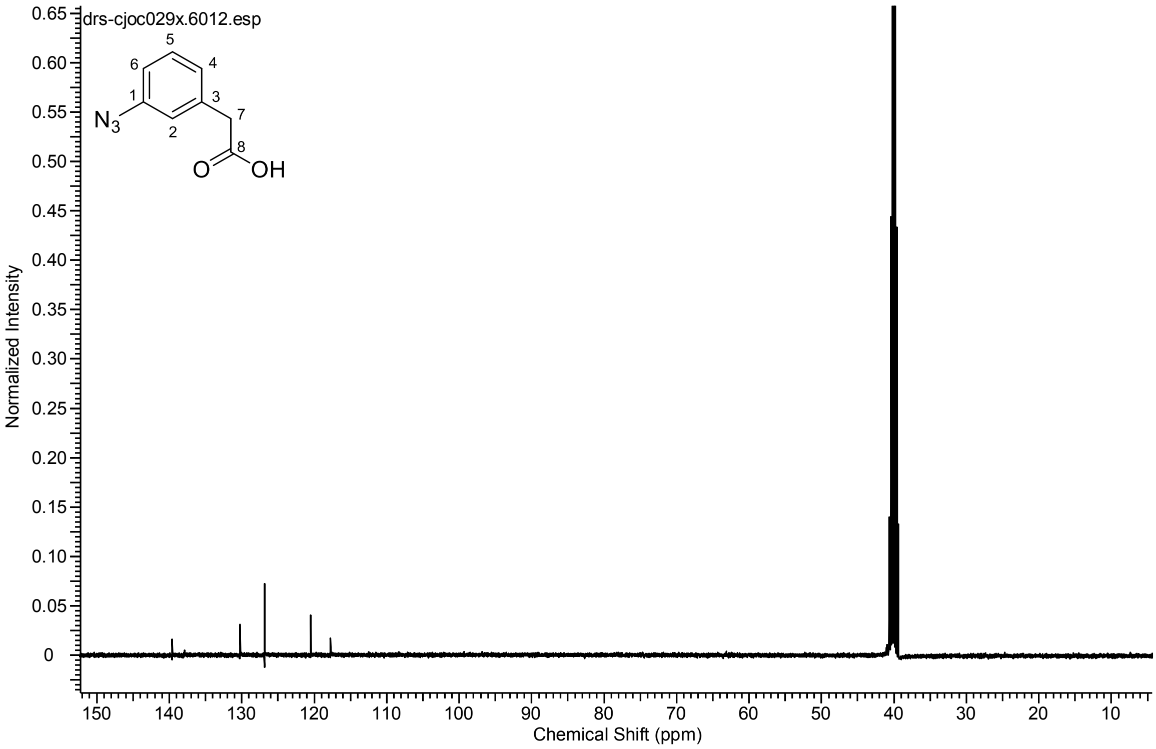
drs-cjoc029x.6012.esp
Normalized Intensity
Chemical Shift (ppm)
N_3
OH
O

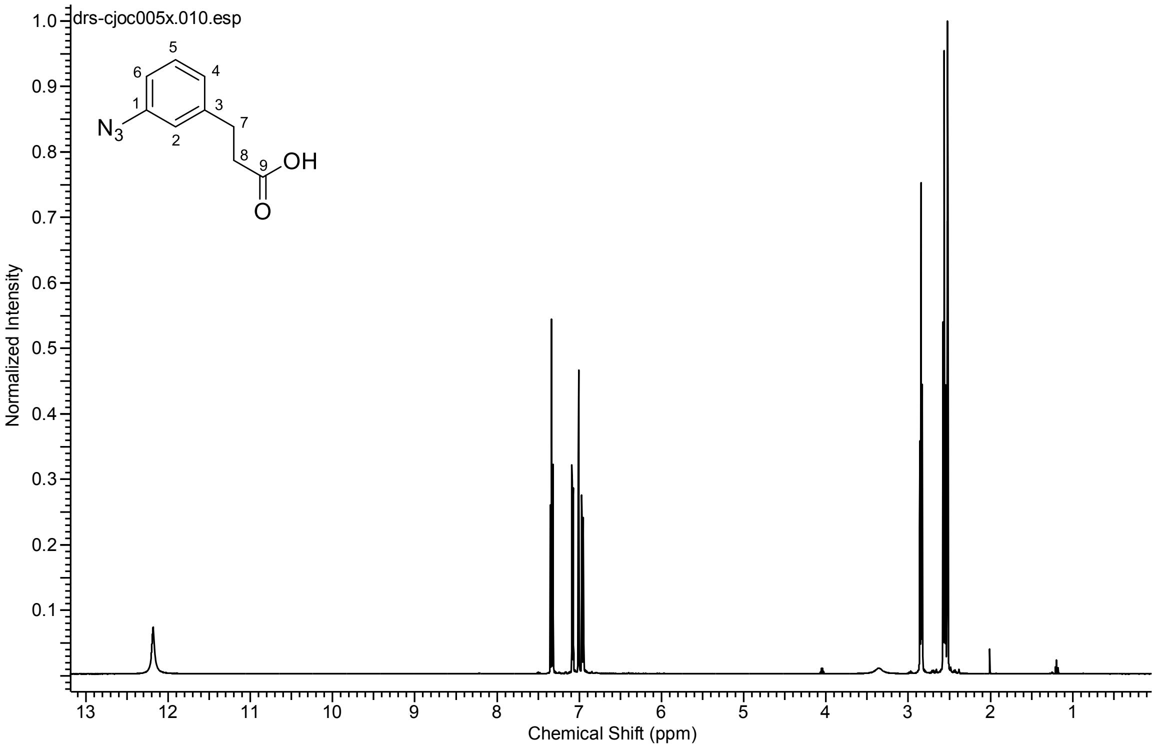

drs-cjoc005x.010.esp
Normalized Intensity
Chemical Shift (ppm)
N3
OH
O

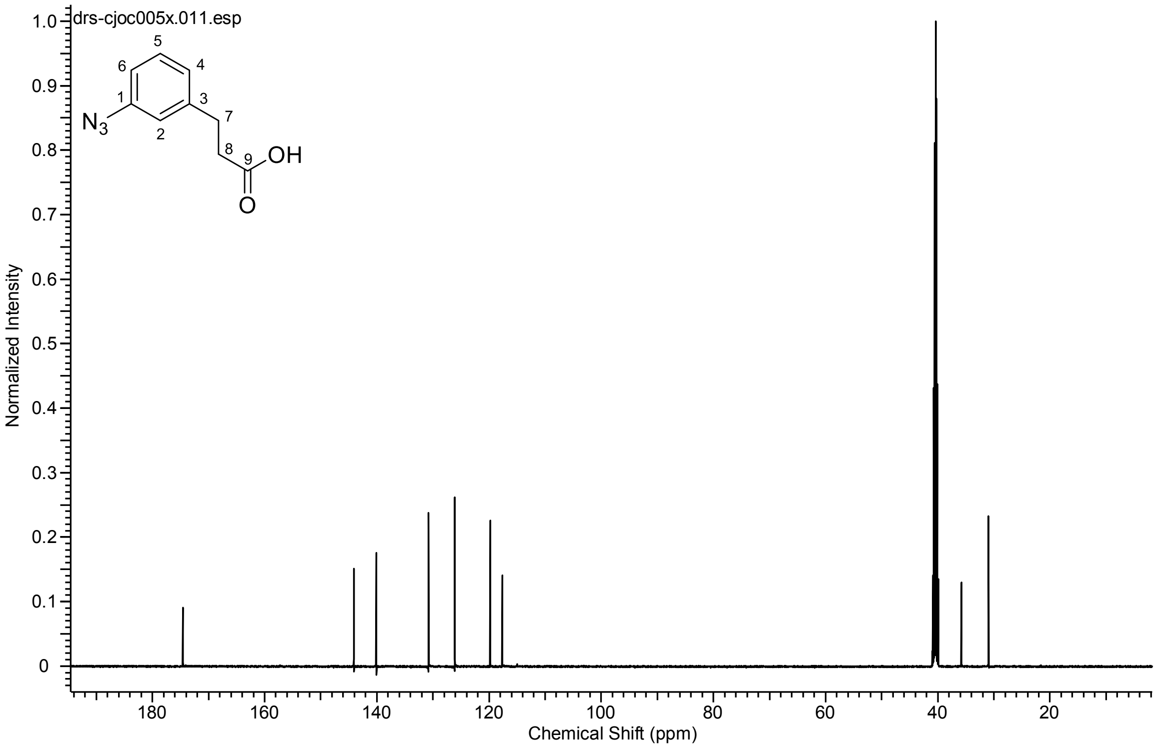

drs-cjoc005x.011.esp
Normalized Intensity
Chemical Shift (ppm)
N3
OH
O

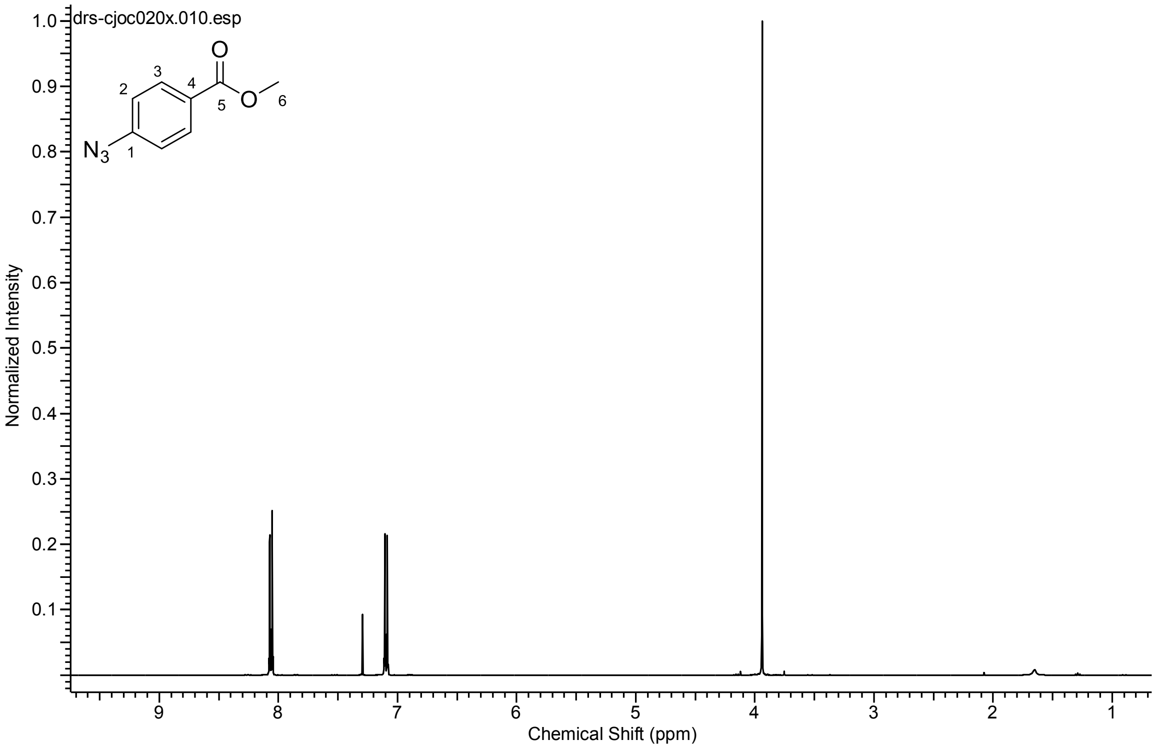
drs-cjoc020x.010.esp
Normalized Intensity
Chemical Shift (ppm)
N3
O
O
1
2
3
4
5
6

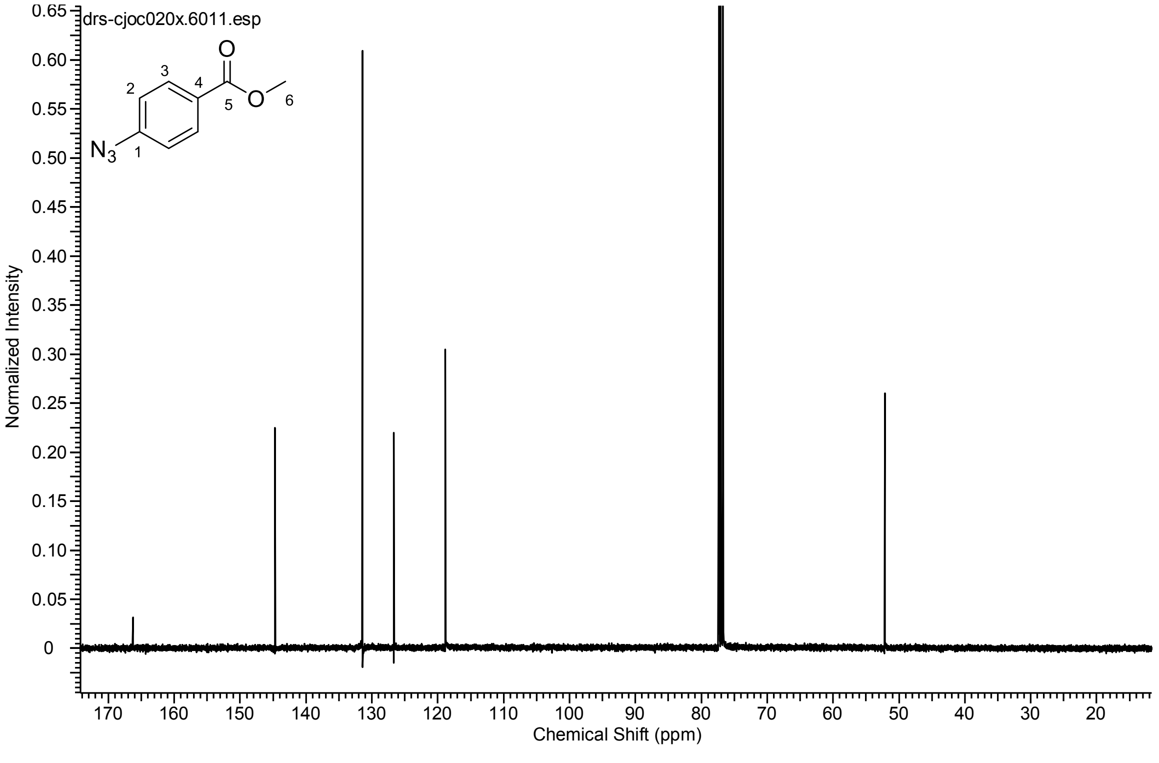
drs-cjoc020x.6011.esp
Normalized Intensity
Chemical Shift (ppm)
N3
O
O
1
2
3
4
5
6

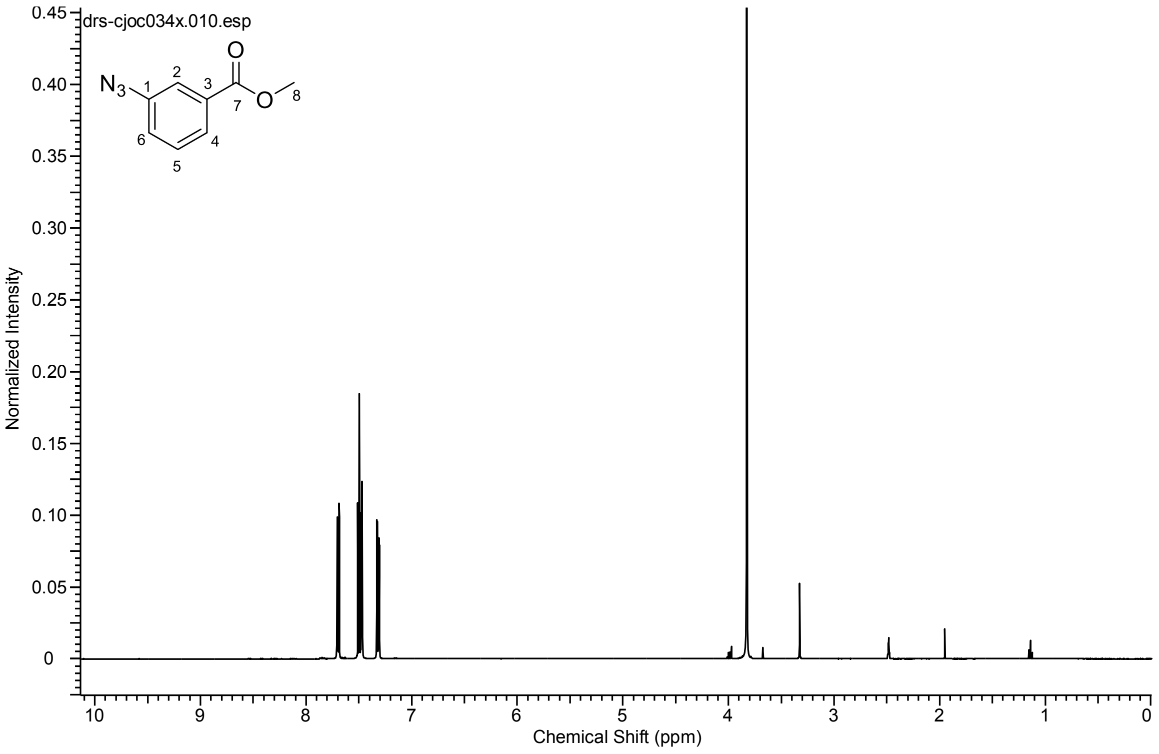

drs-cjoc034x.010.esp
N_3
1
2
3
4
5
6
7
8
O
O
Normalized Intensity
Chemical Shift (ppm)

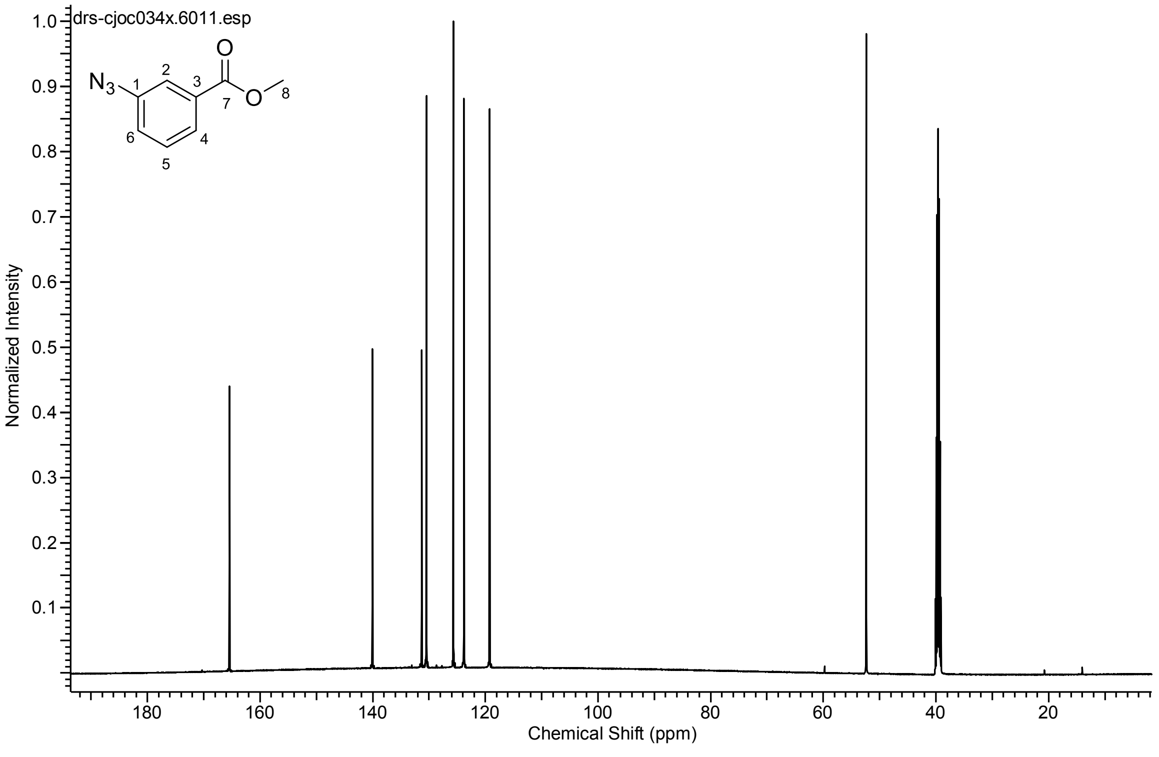

drs-cjoc034x.6011.esp
N_3
1
2
3
4
5
6
7
8
O
O
Normalized Intensity
Chemical Shift (ppm)

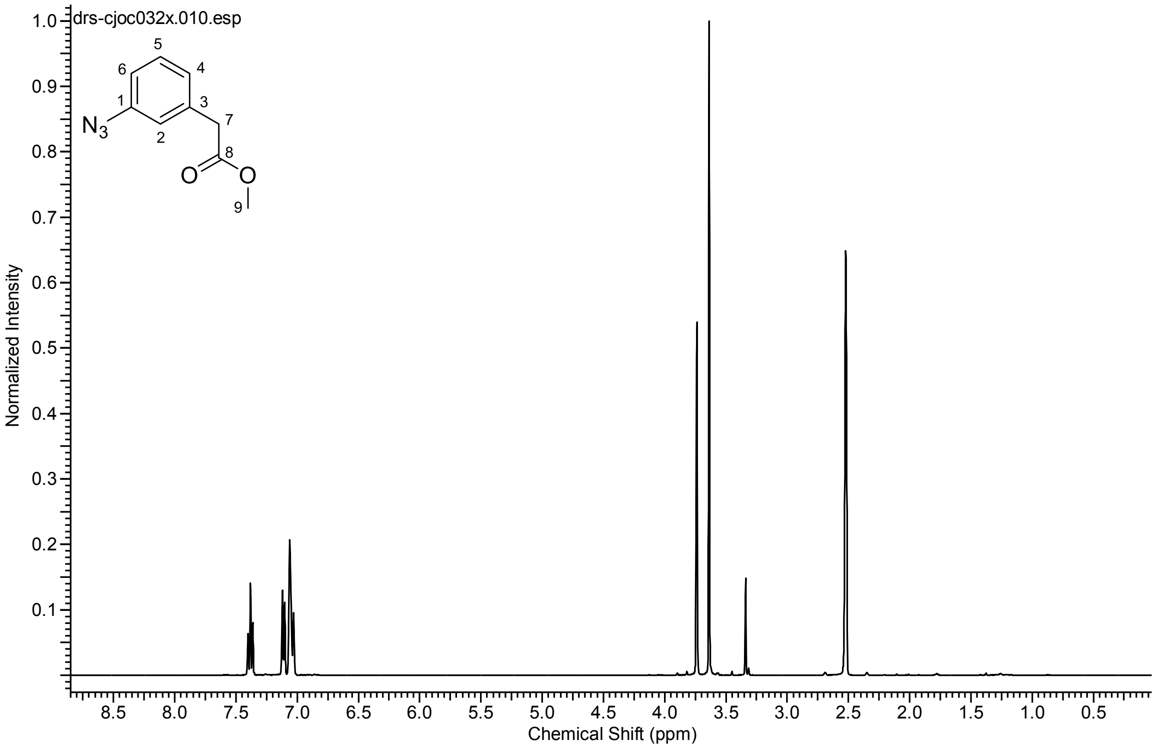
drs-cjoc032x.010.esp
N3
Normalized Intensity
Chemical Shift (ppm)

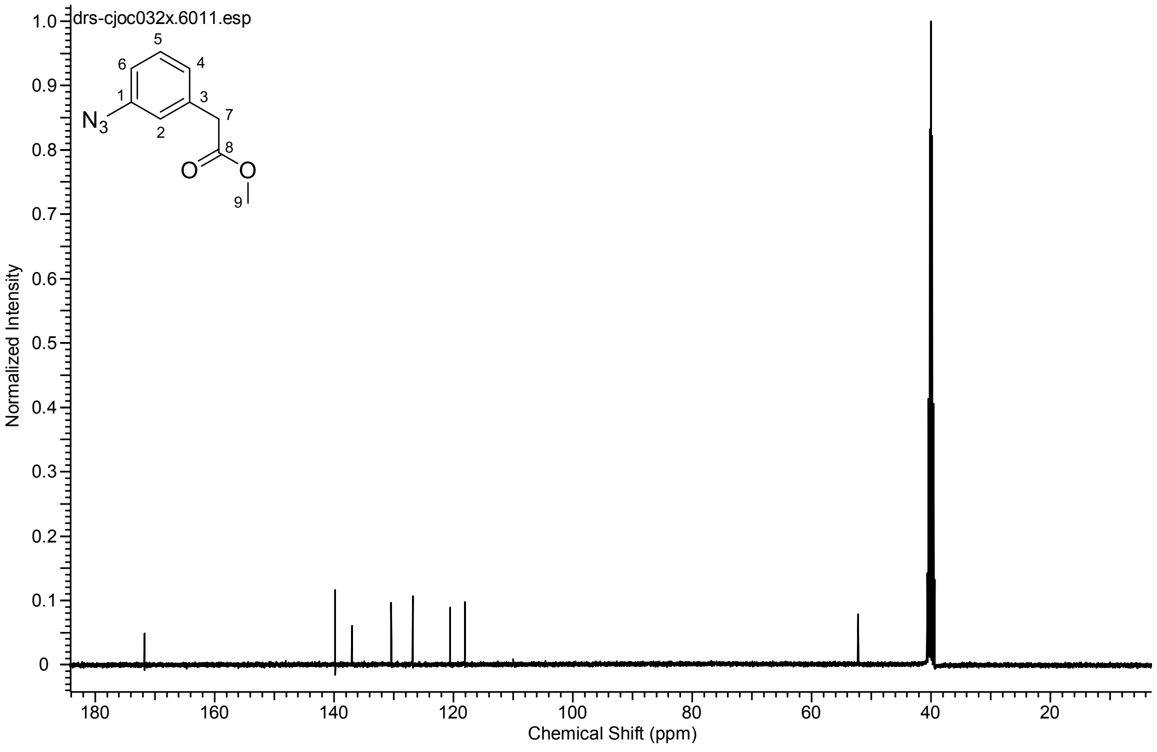
drs-cjoc032x.6011.esp
N3
Normalized Intensity
Chemical Shift (ppm)

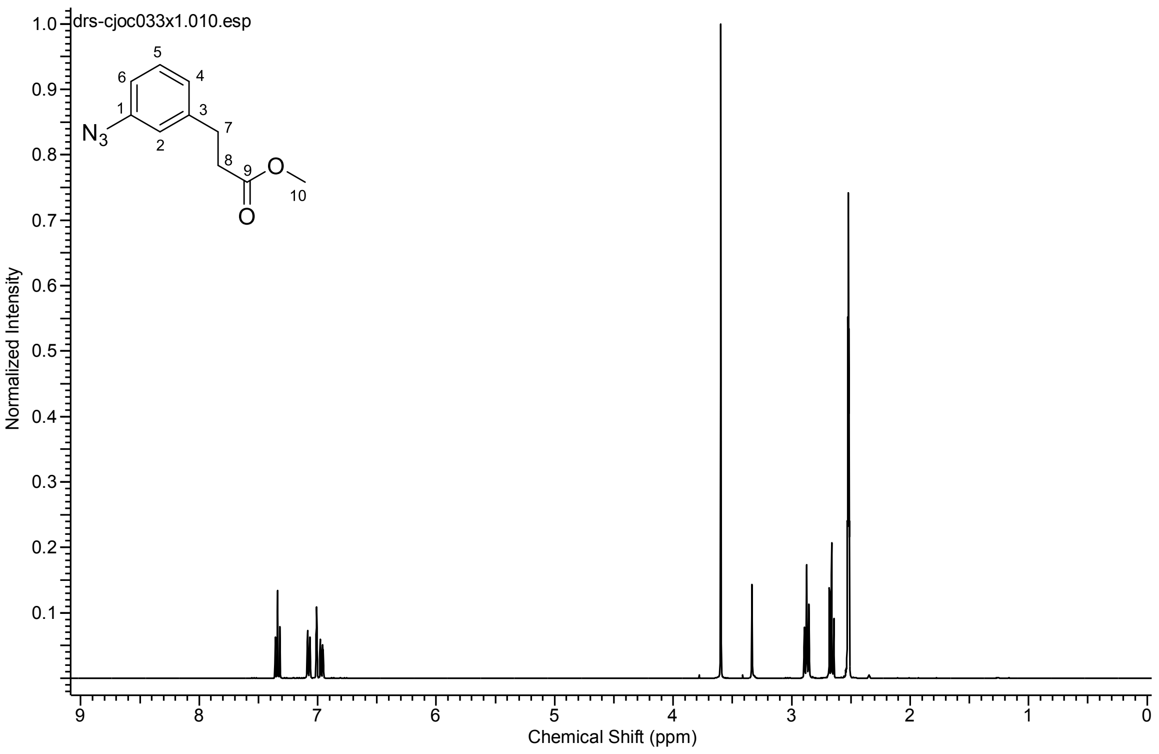
drs-cjoc033x1.010.esp
Normalized Intensity
Chemical Shift (ppm)

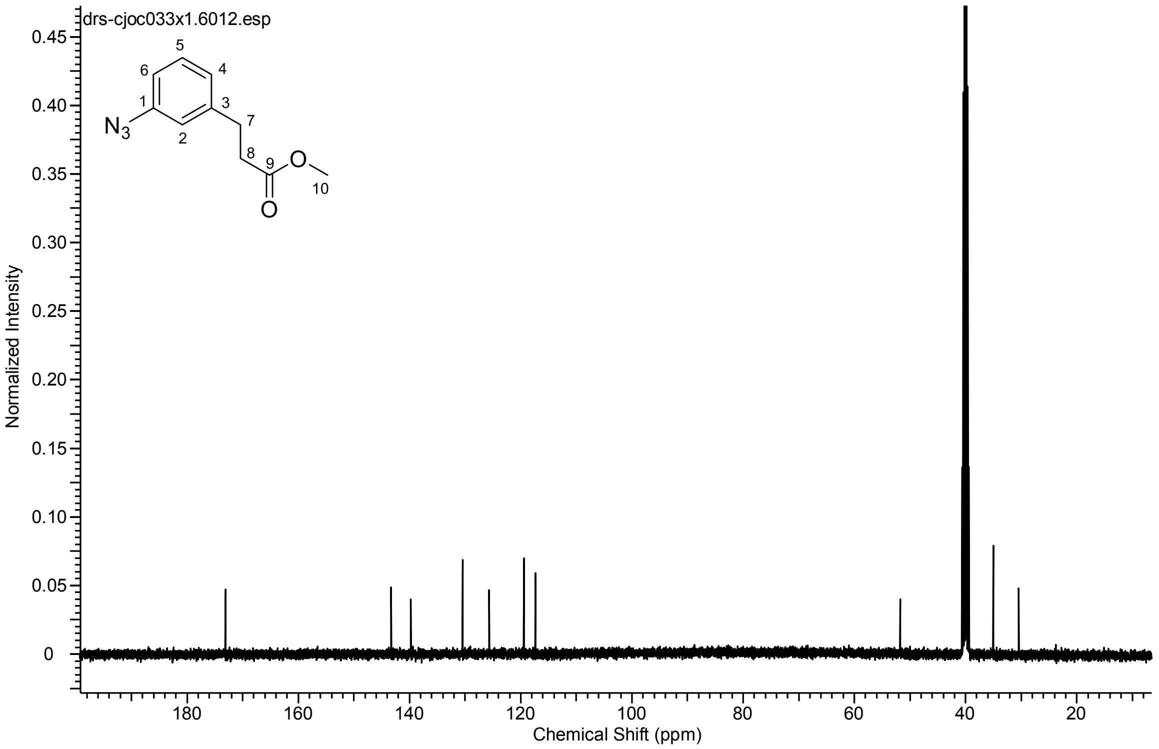
drs-cjoc033x1.6012.esp
Normalized Intensity
Chemical Shift (ppm)

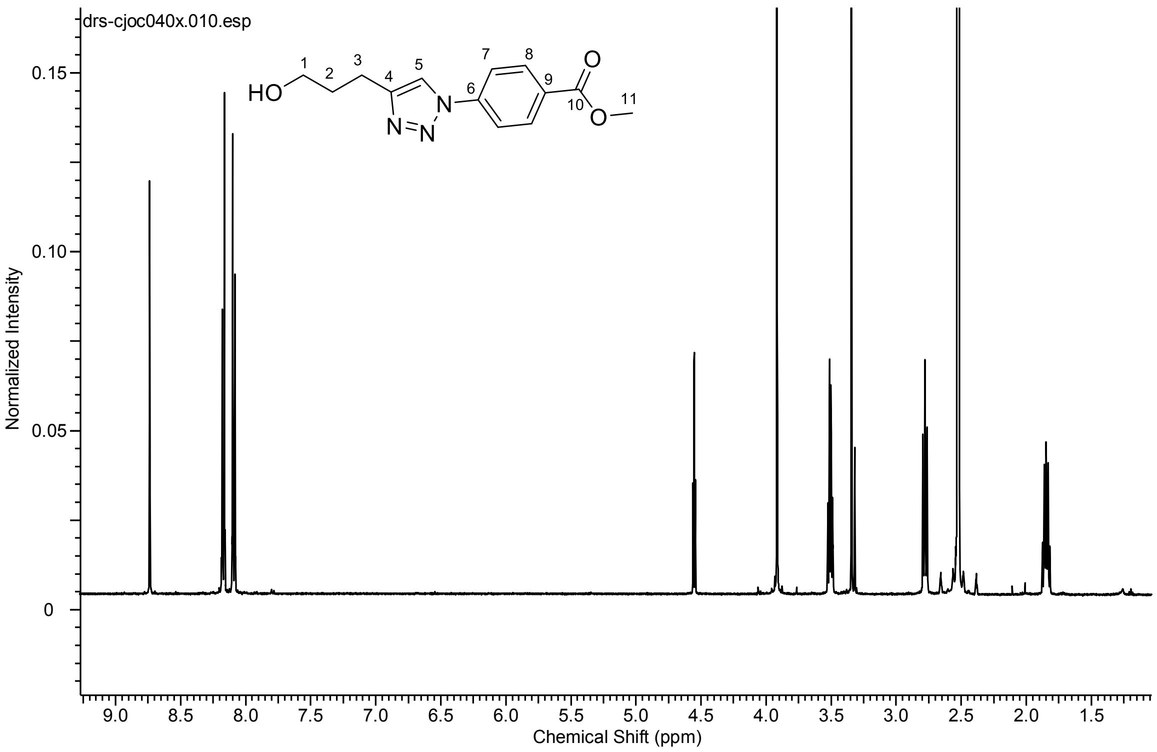
drs-cjoc040x.010.esp
Normalized Intensity
Chemical Shift (ppm)
HO
N
N=N
O
O
1
2
3
4
5
6
7
8
9
10
11

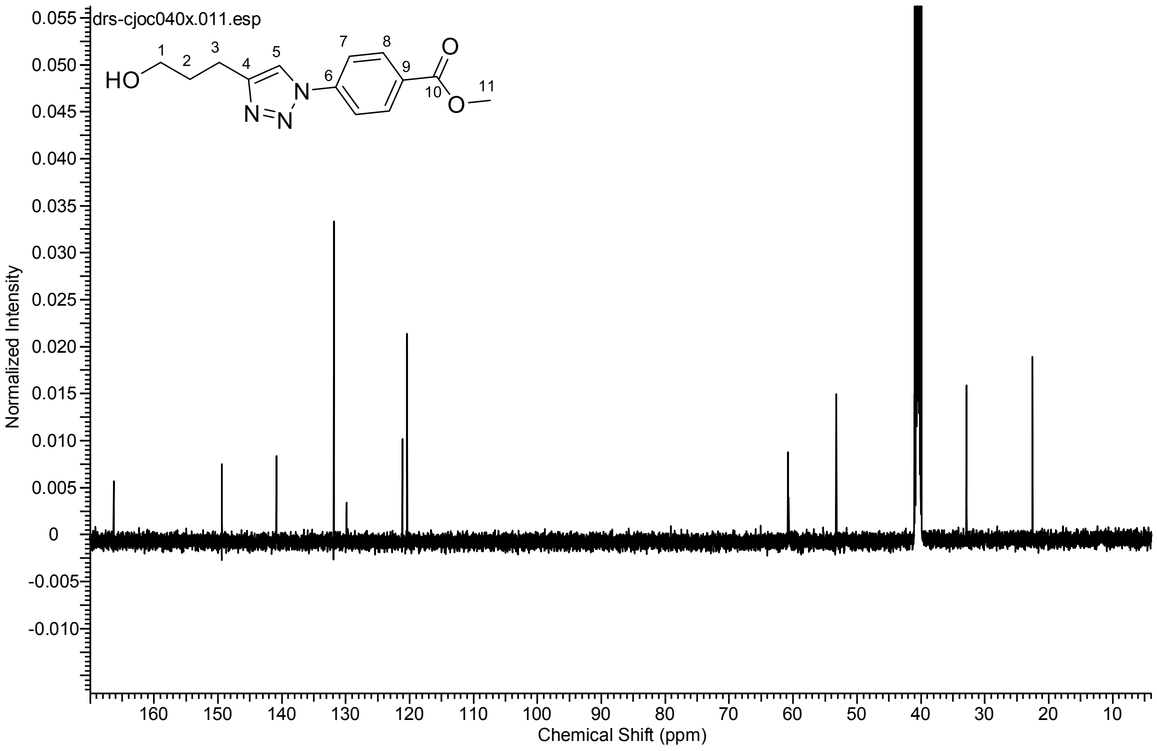
drs-cjoc040x.011.esp
Normalized Intensity
Chemical Shift (ppm)
HO
N
N=N
O
O
1
2
3
4
5
6
7
8
9
10
11

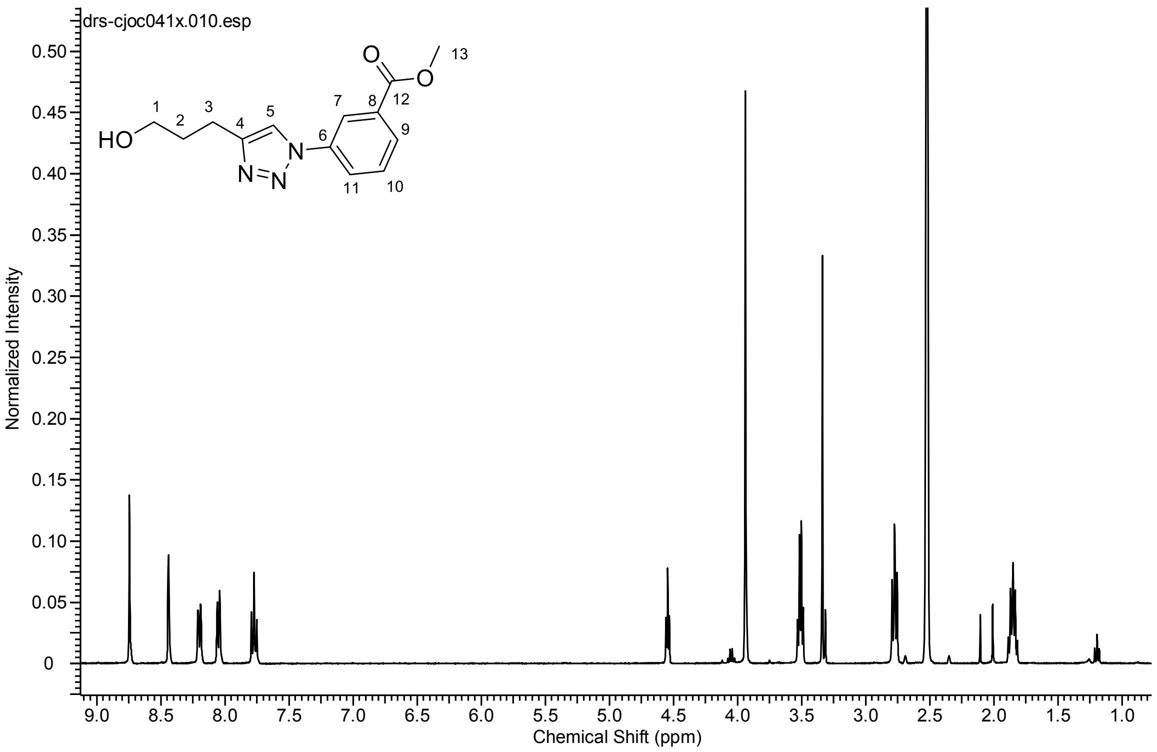
drs-cjoc041x.010.esp
Normalized Intensity
Chemical Shift (ppm)
HO
N
N=N
O
O
1
2
3
4
5
6
7
8
9
10
11
12
13

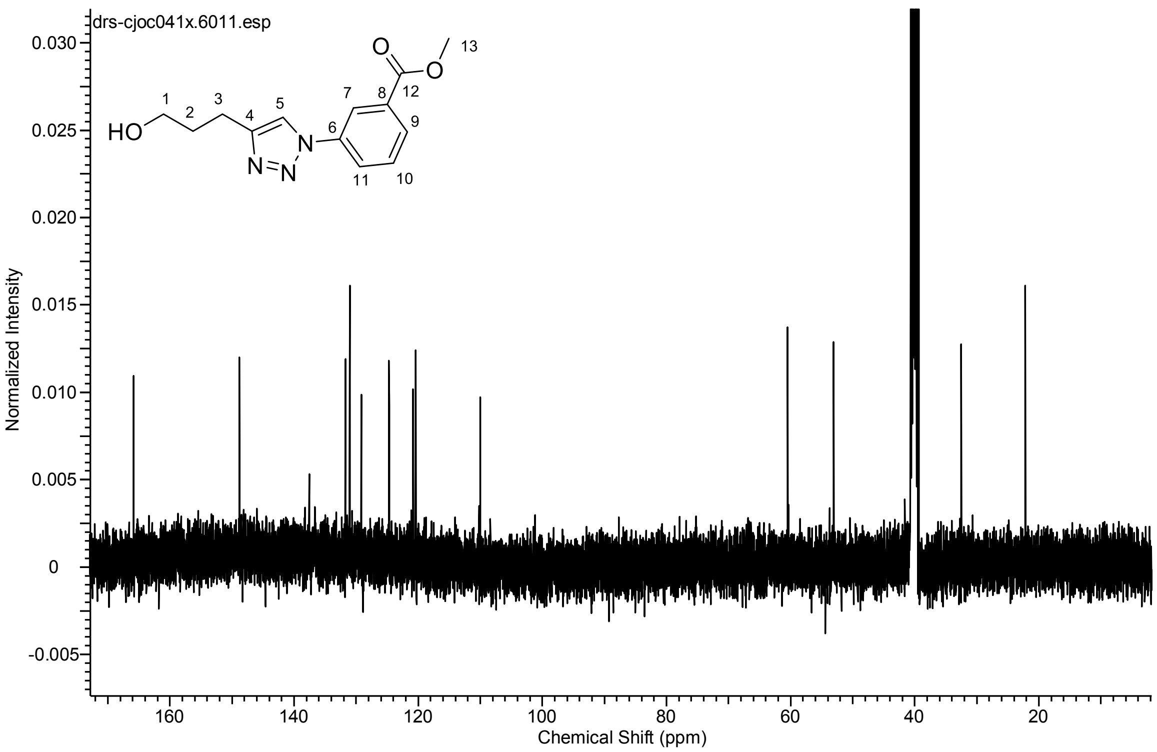
drs-cjoc041x.6011.esp
Normalized Intensity
Chemical Shift (ppm)
HO
N
N=N
O
O
1
2
3
4
5
6
7
8
9
10
11
12
13

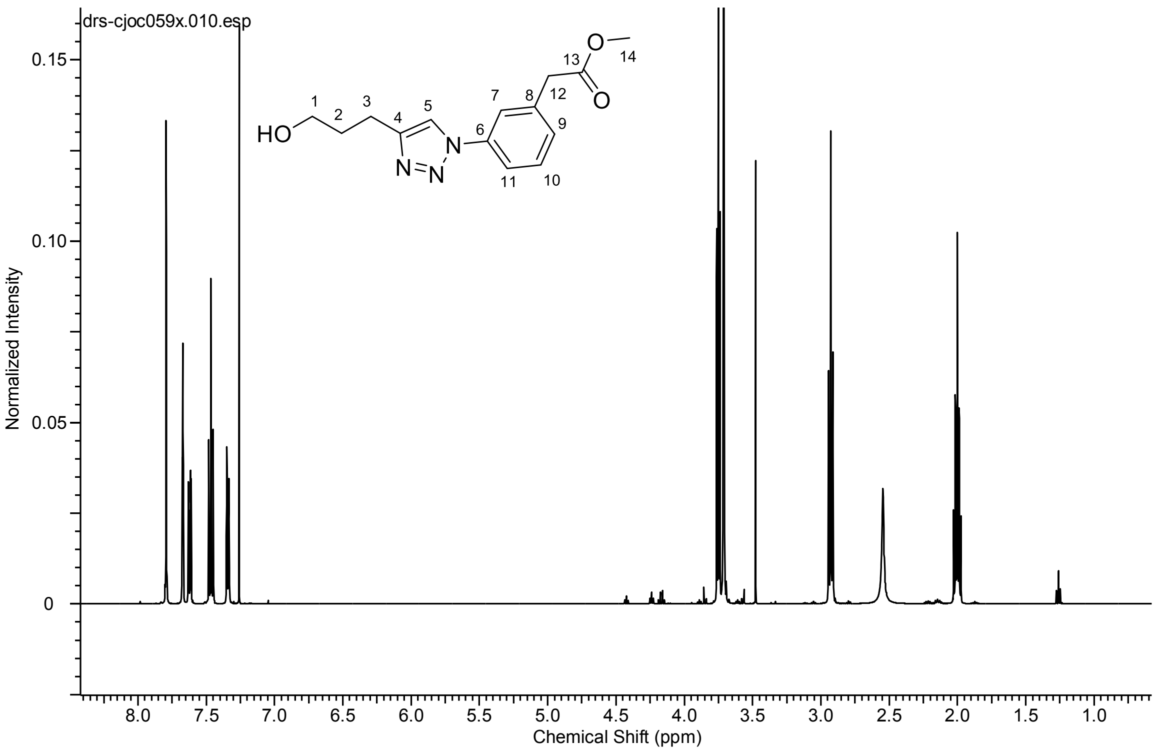
drs-cjoc059x.010.esp
Normalized Intensity
Chemical Shift (ppm)
HO
N
N
N
1
2
3
4
5
6
7
8
9
10
11
12
13
14
O
O

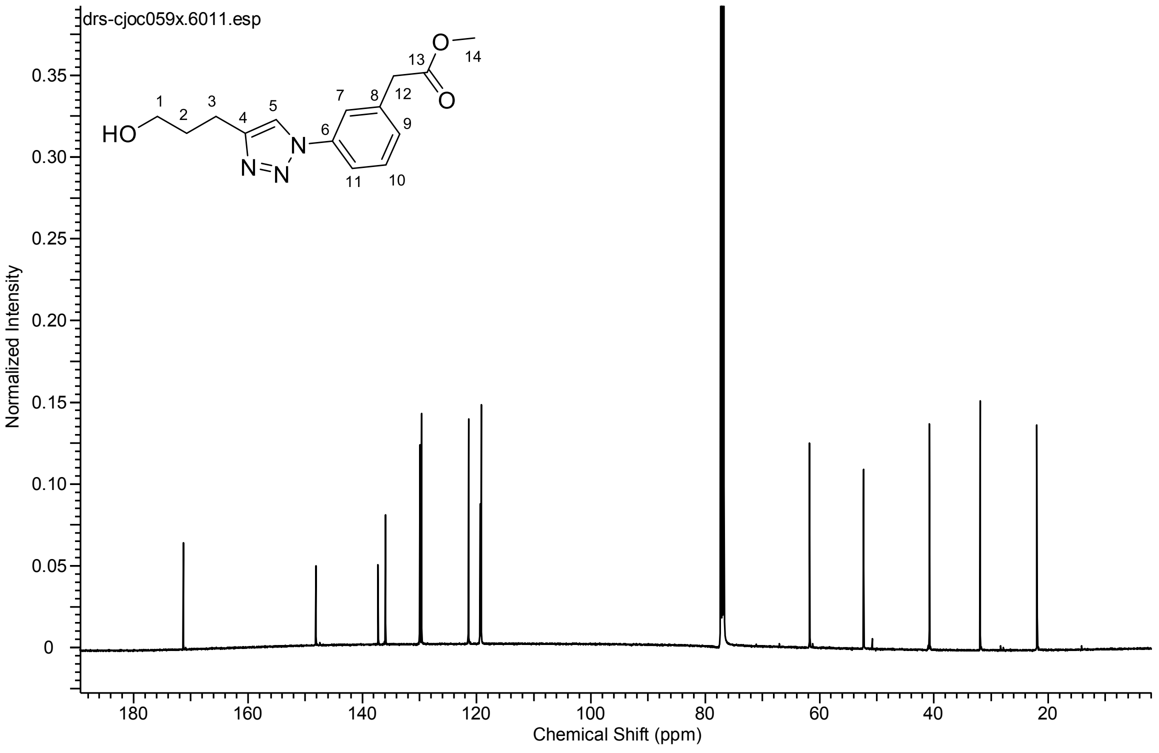
drs-cjoc059x.6011.esp
Normalized Intensity
Chemical Shift (ppm)
HO
N
N
N
1
2
3
4
5
6
7
8
9
10
11
12
13
14
O
O

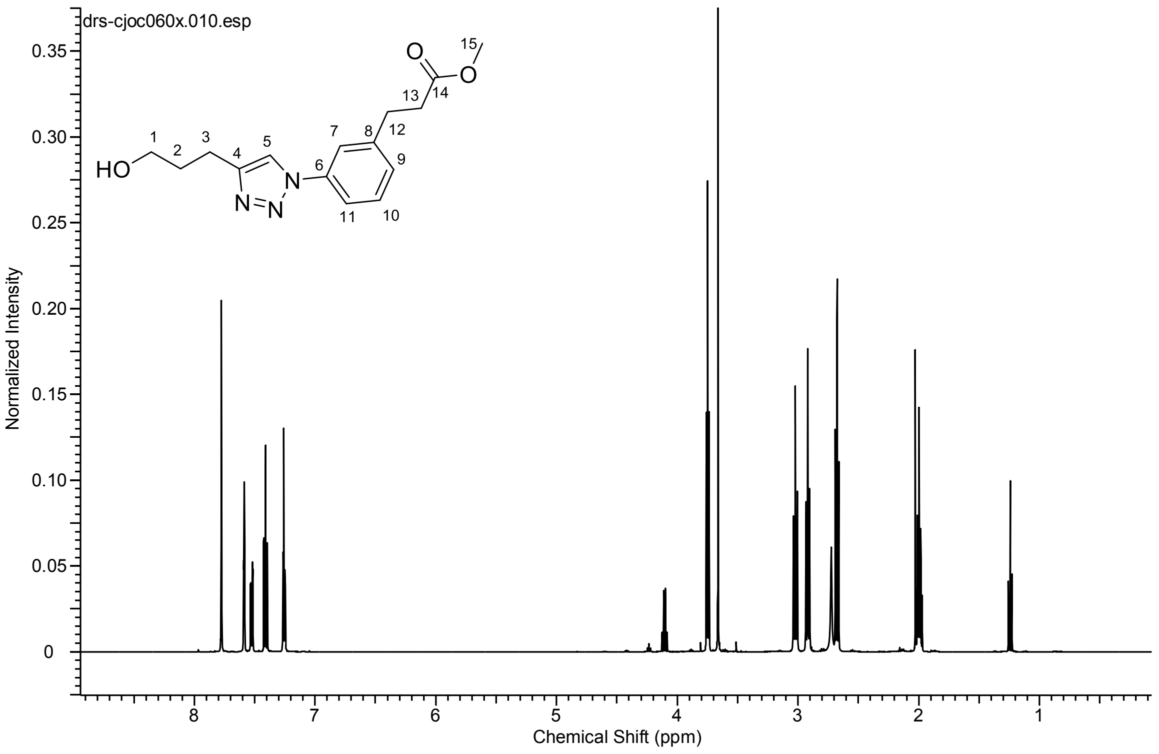
drs-cjoc060x.010.esp
Normalized Intensity
Chemical Shift (ppm)
HO
N
N=N
O
O
1
2
3
4
5
6
7
8
9
10
11
12
13
14
15

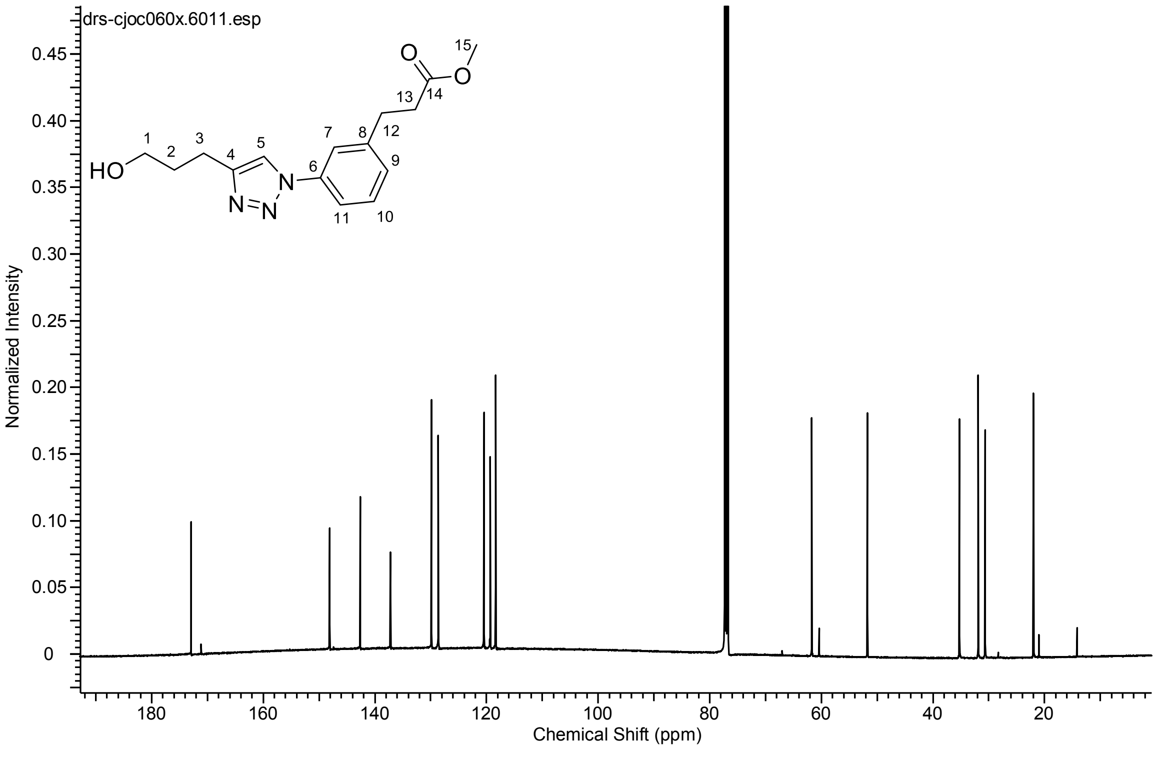
drs-cjoc060x.6011.esp
Normalized Intensity
Chemical Shift (ppm)
HO
N
N=N
O
O
1
2
3
4
5
6
7
8
9
10
11
12
13
14
15

cjoc42

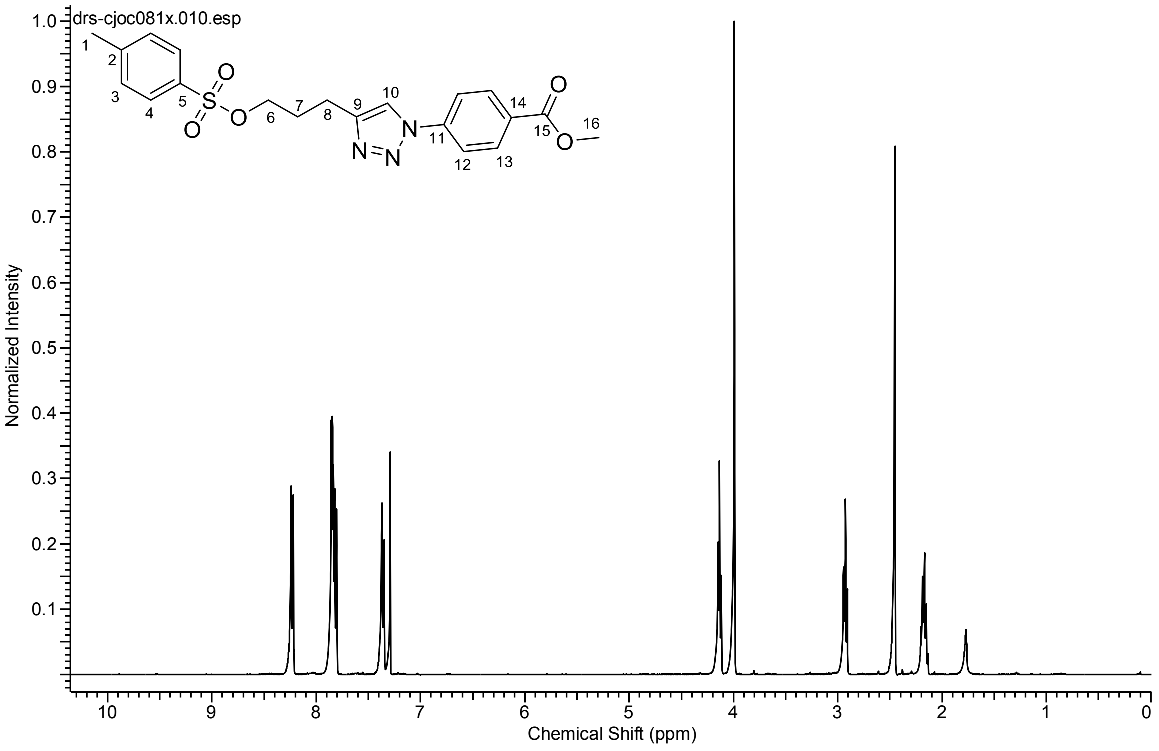
drs-cjoc081x.010.esp
Normalized Intensity
Chemical Shift (ppm)
1
2
3
4
5
6
7
8
9
10
11
12
13
14
15
16
S
O
O
O
N
N
N
O
O

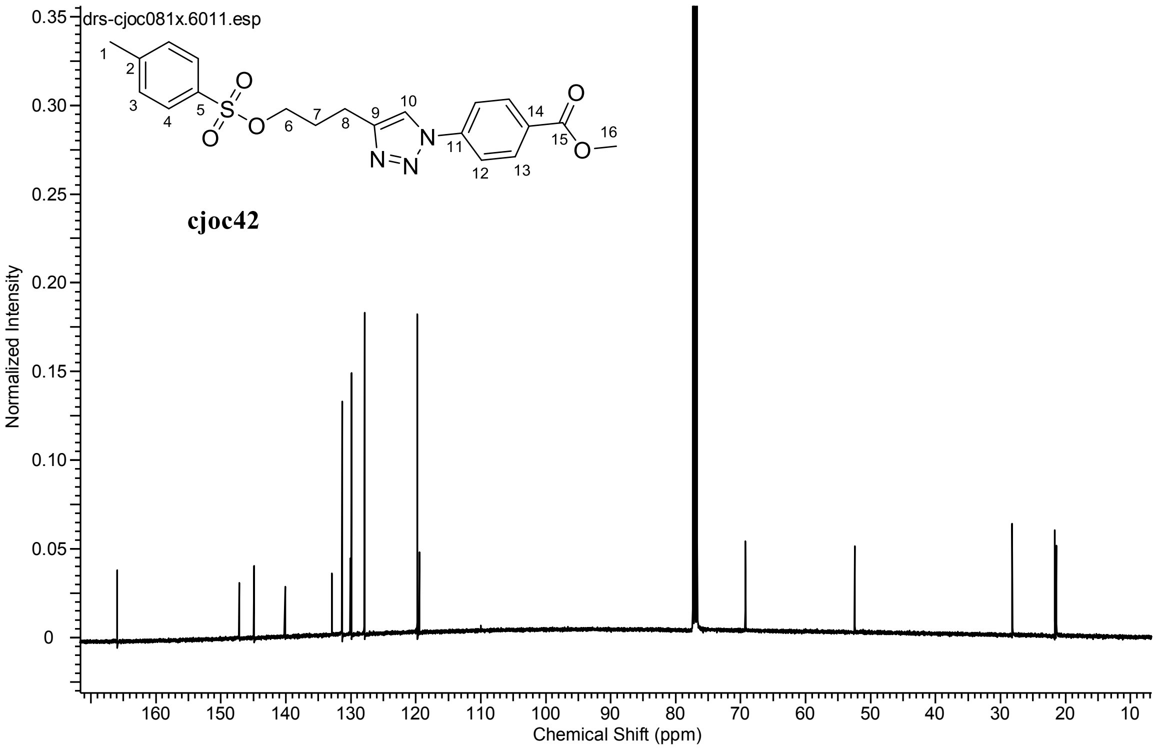
drs-cjoc081x.6011.esp
cjoc42
Normalized Intensity
Chemical Shift (ppm)

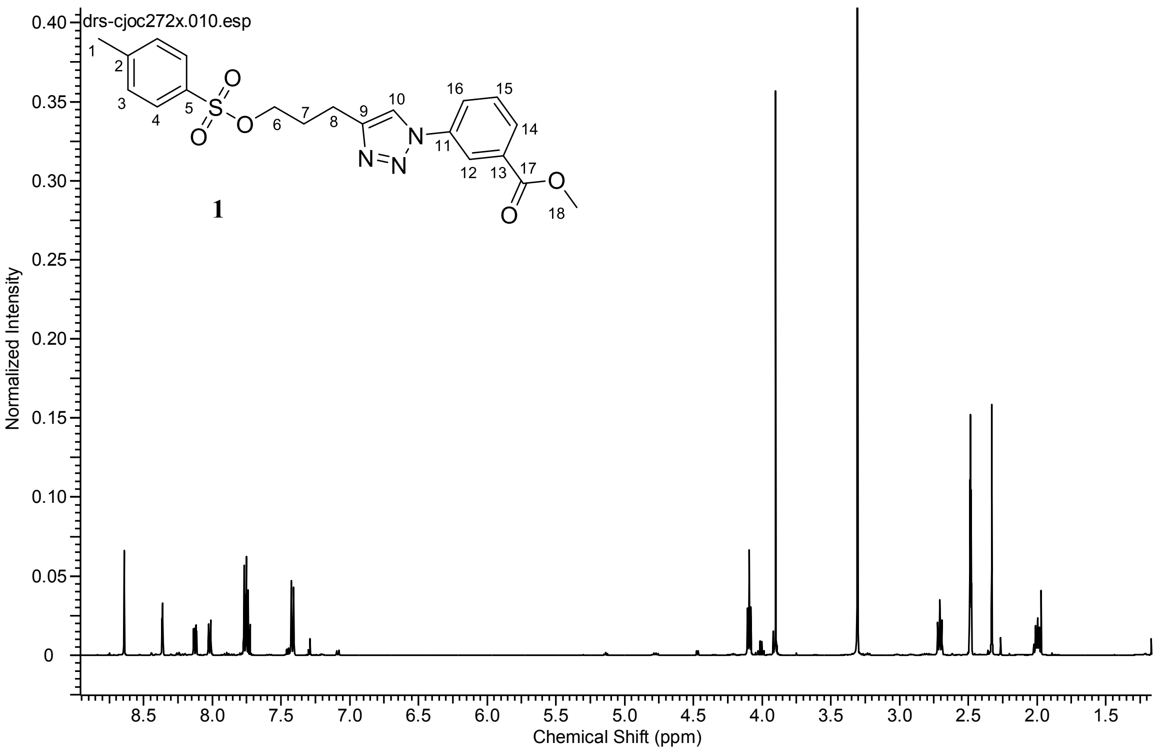
drs-cjoc272x.010.esp
1
Normalized Intensity
Chemical Shift (ppm)

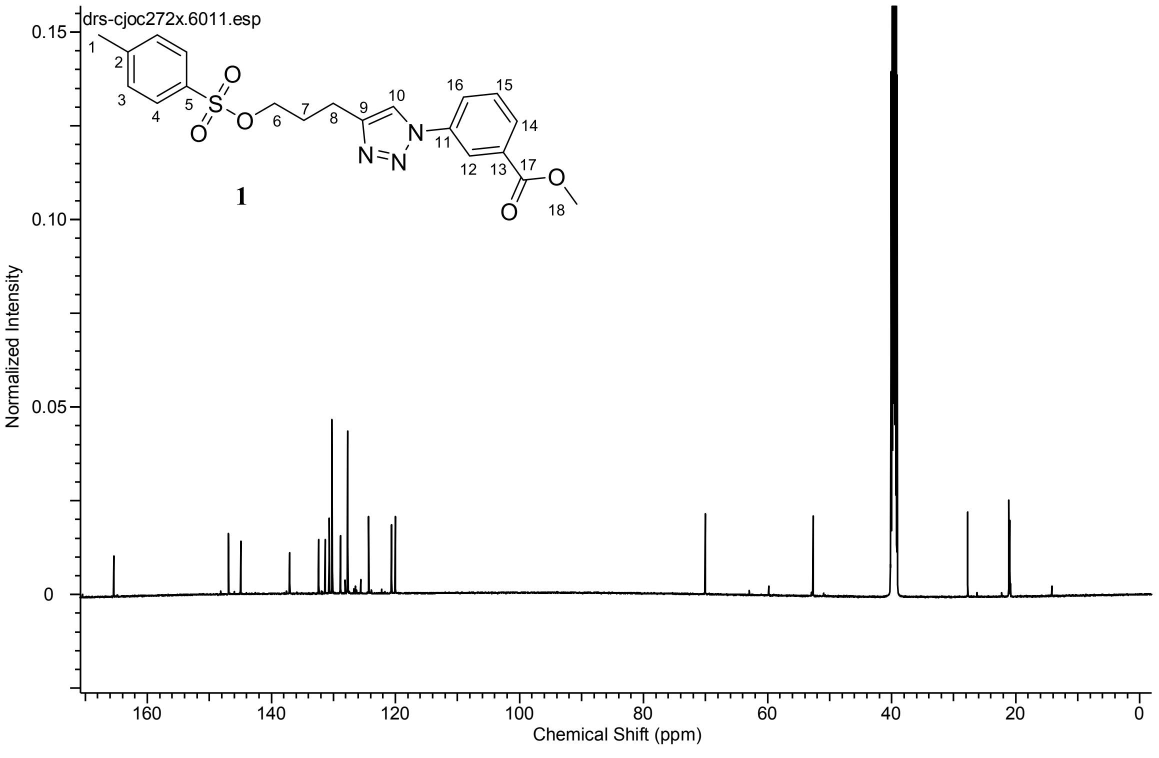
drs-cjoc272x.6011.esp
1
Normalized Intensity
Chemical Shift (ppm)

2

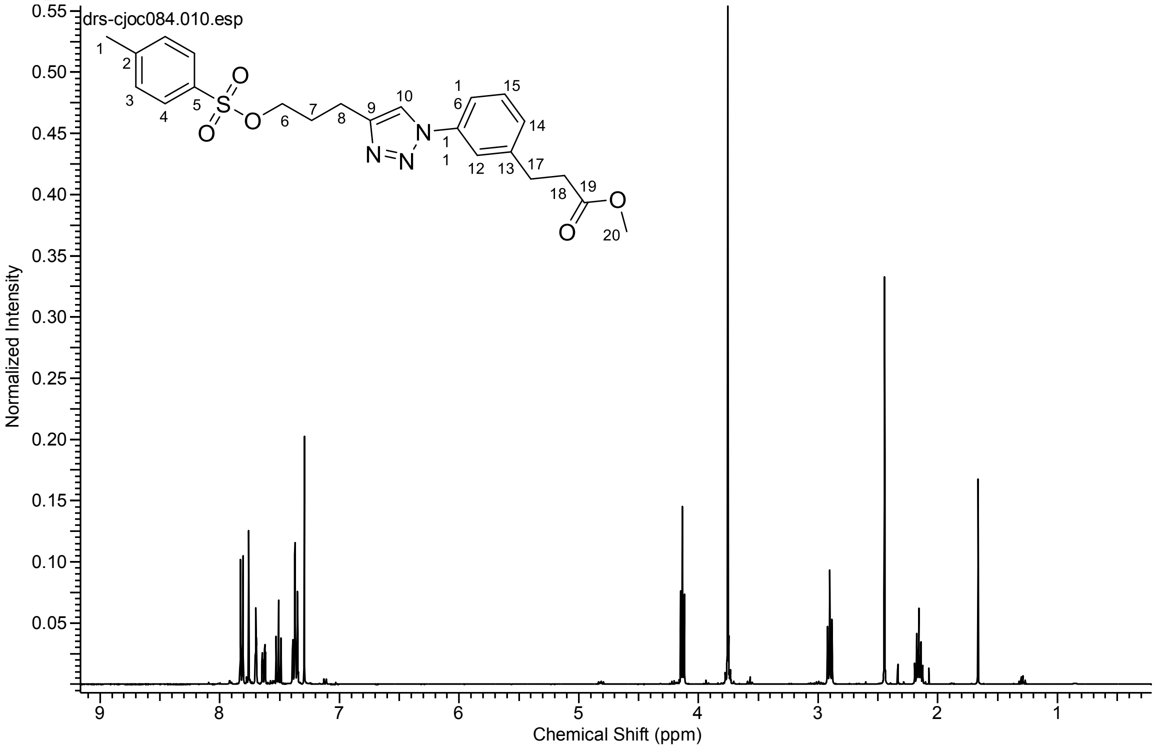
drs-cjoc084.010.esp
Normalized Intensity
Chemical Shift (ppm)

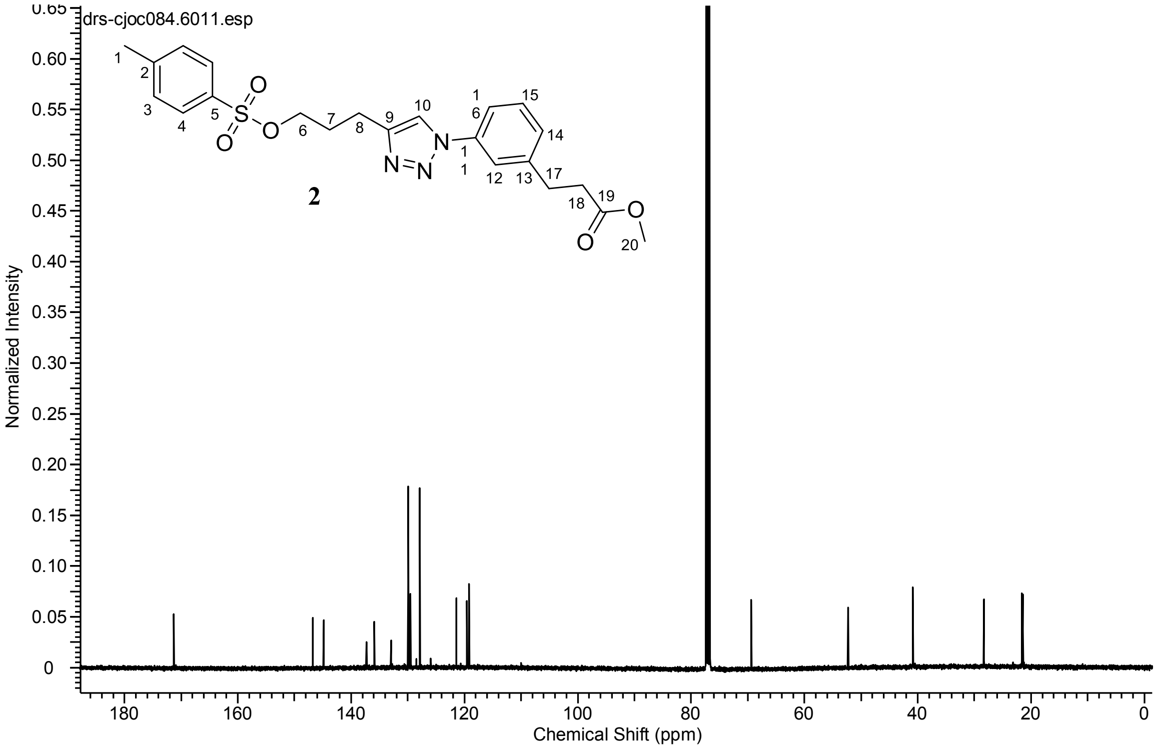
drs-cjoc084.6011.esp
2
Normalized Intensity
Chemical Shift (ppm)

3

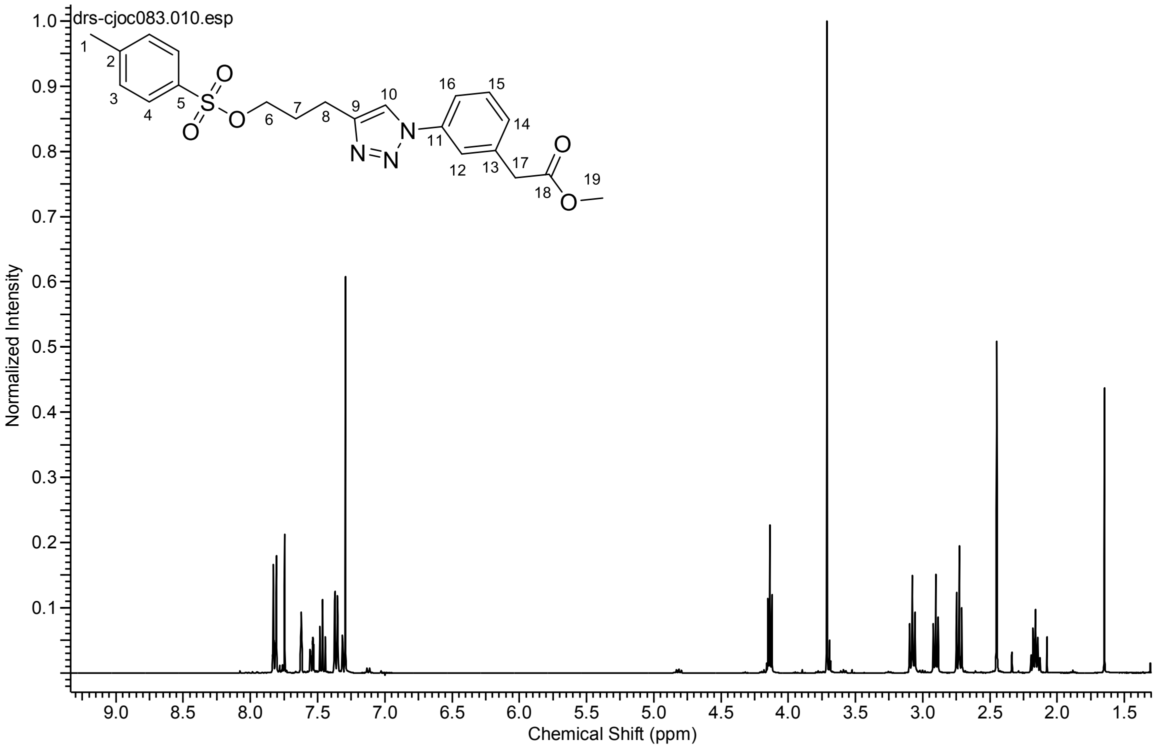
drs-cjoc083.010.esp
Normalized Intensity
Chemical Shift (ppm)

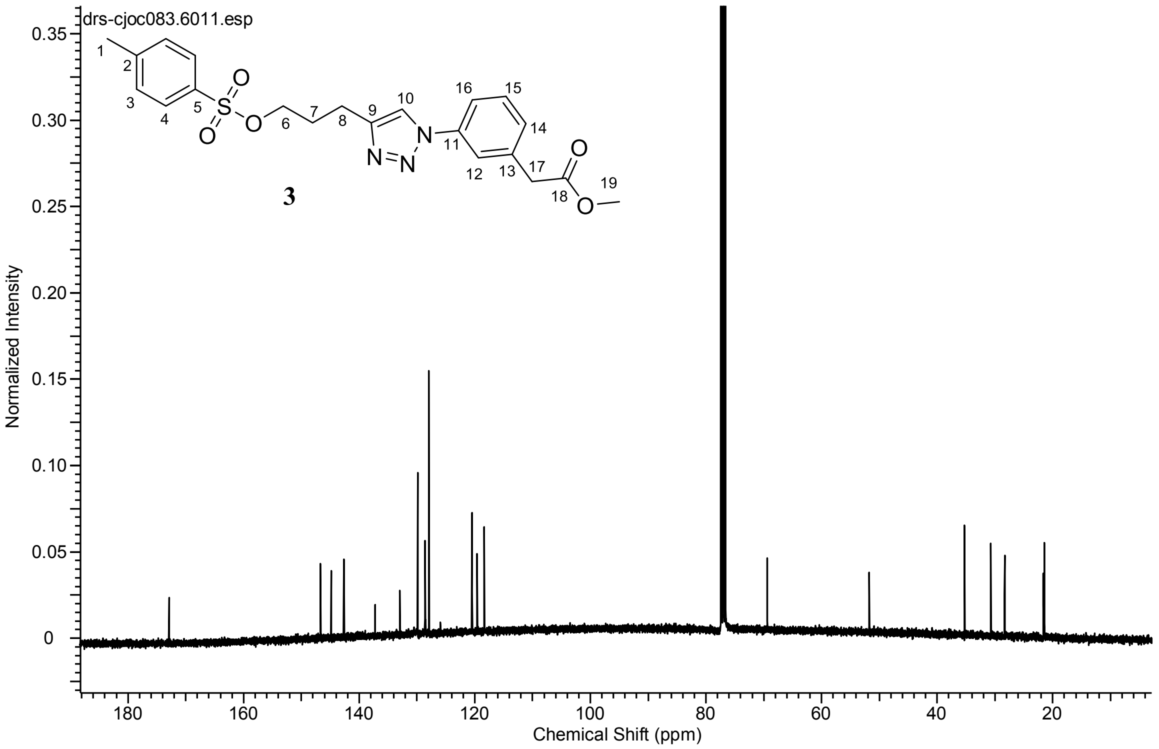
drs-cjoc083.6011.esp
3
Normalized Intensity
Chemical Shift (ppm)

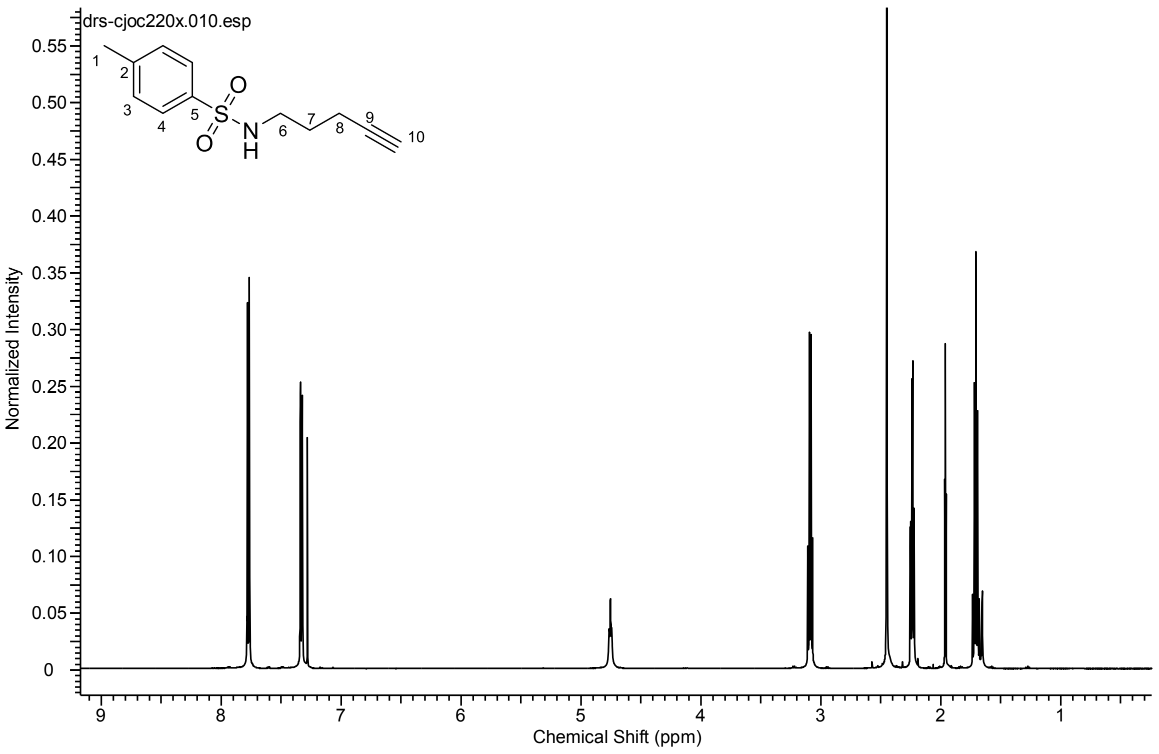
drs-cjoc220x.010.esp
Normalized Intensity
Chemical Shift (ppm)

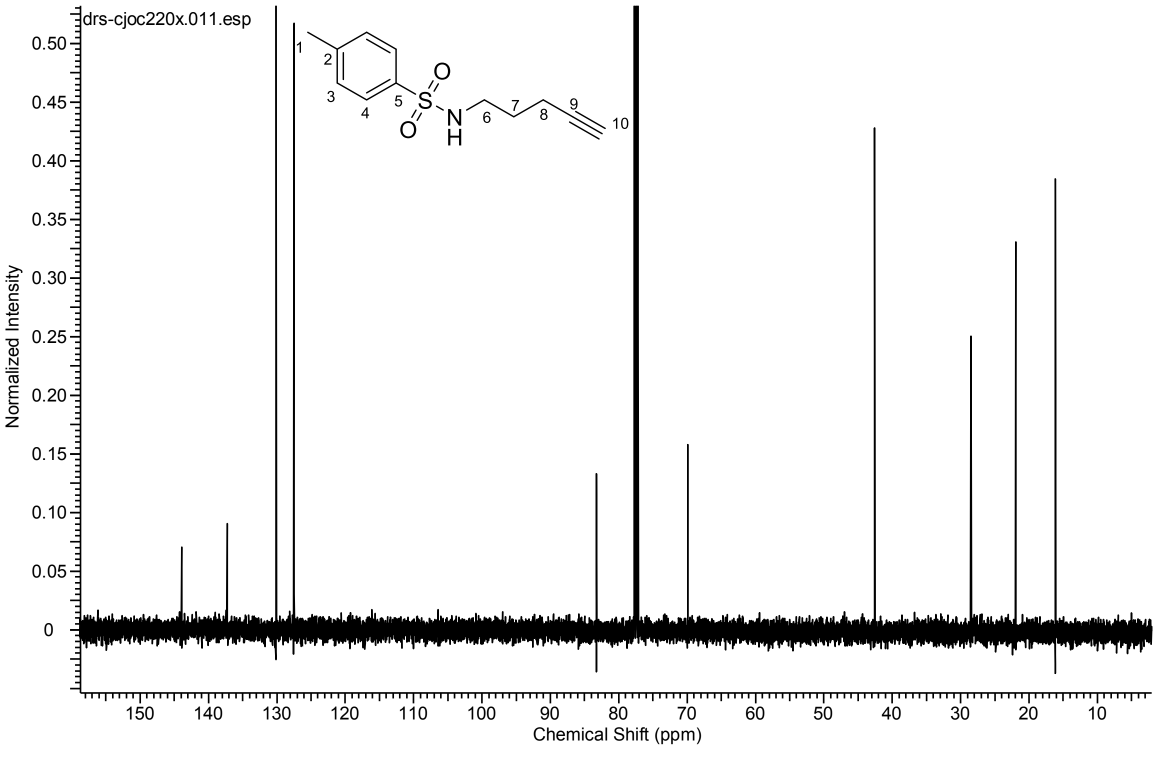
drs-cjoc220x.011.esp
Normalized Intensity
Chemical Shift (ppm)

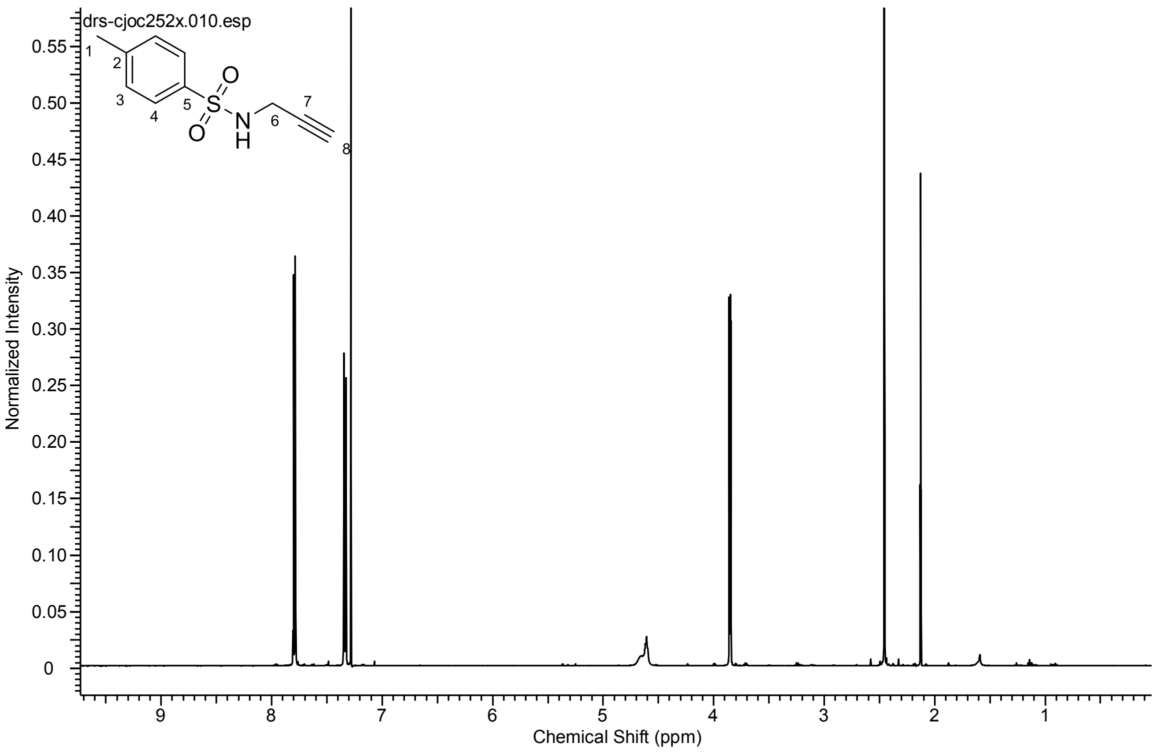
drs-cjoc252x.010.esp
Normalized Intensity
Chemical Shift (ppm)
1
2
3
4
5
6
7
8
O
S
O
N
H

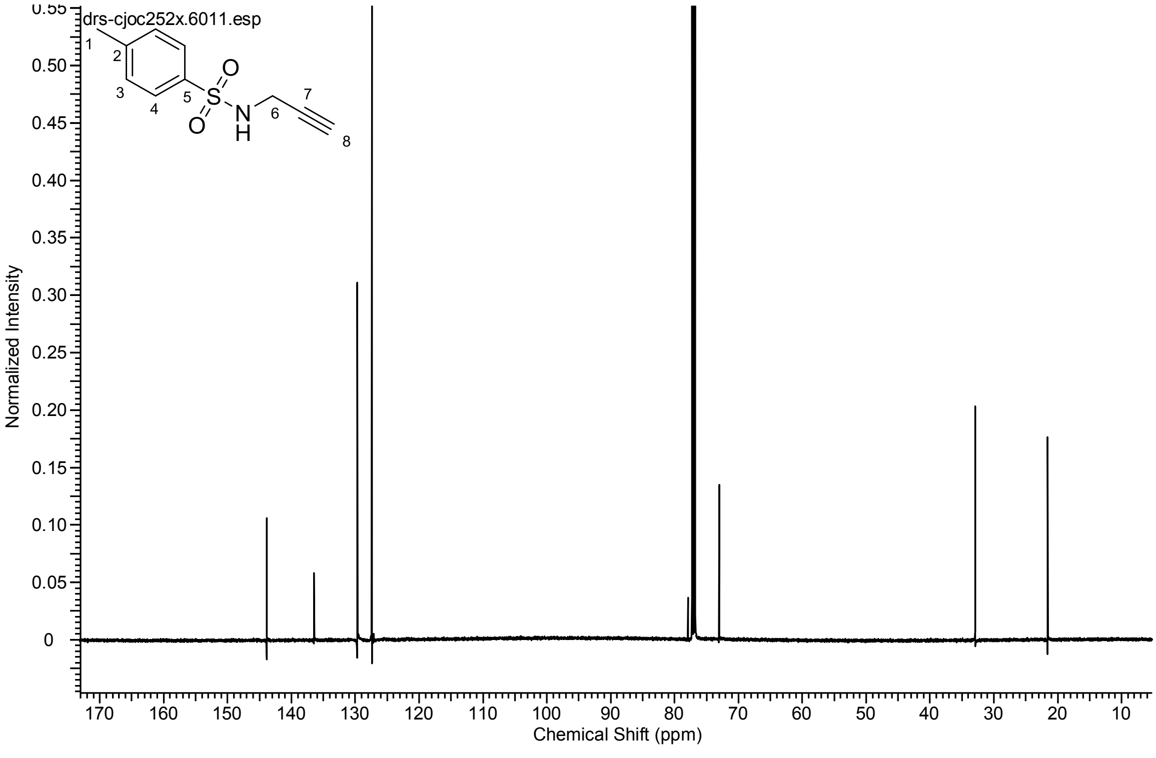
drs-cjoc252x.6011.esp
Normalized Intensity
Chemical Shift (ppm)
1
2
3
4
5
6
7
8
O
S
O
N
H

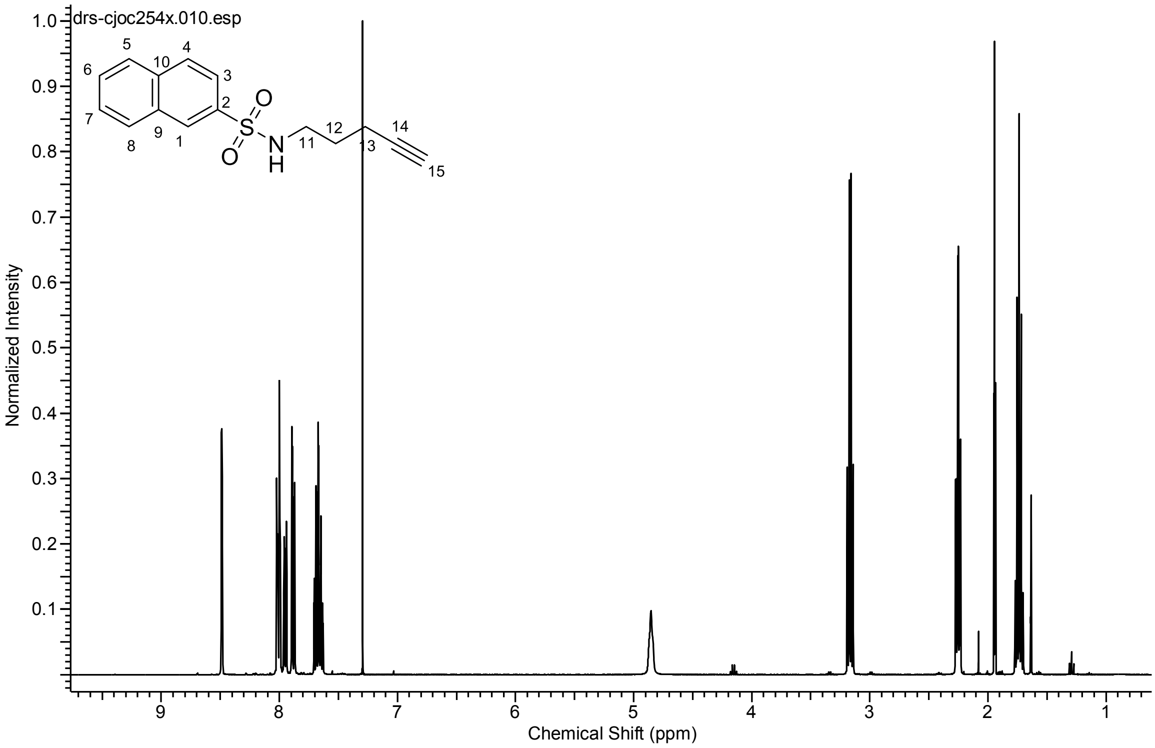
drs-cjoc254x.010.esp
Normalized Intensity
Chemical Shift (ppm)

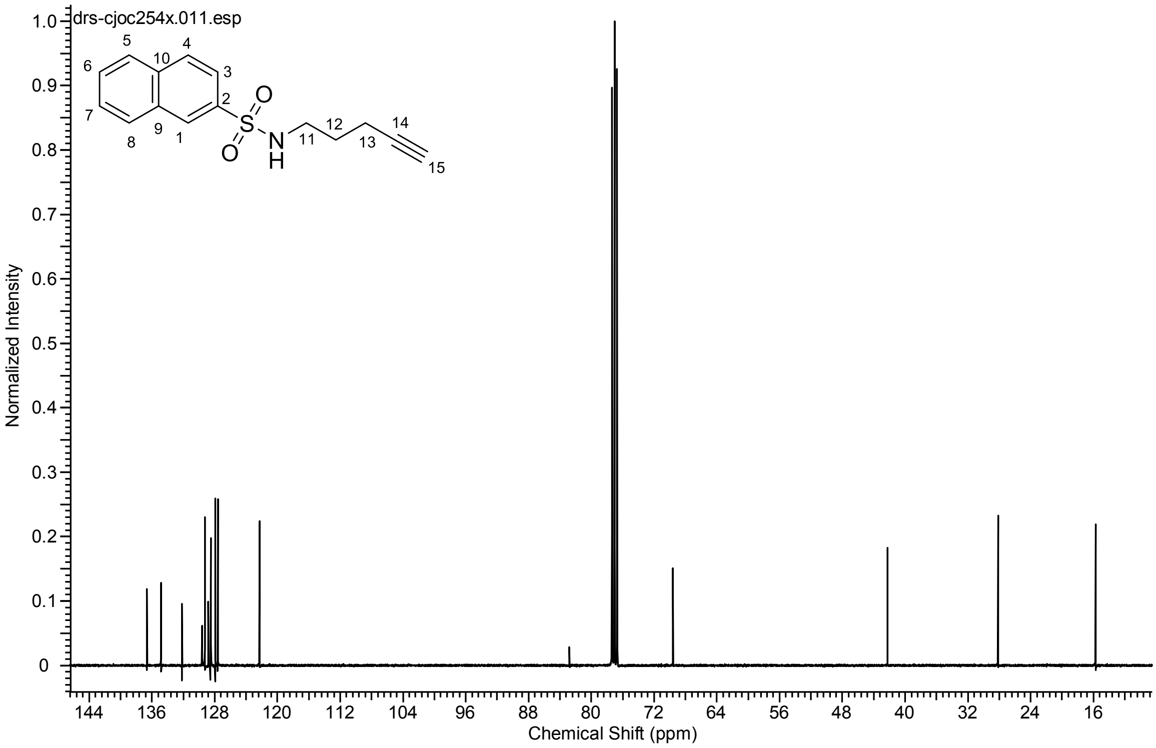
drs-cjoc254x.011.esp
Normalized Intensity
Chemical Shift (ppm)

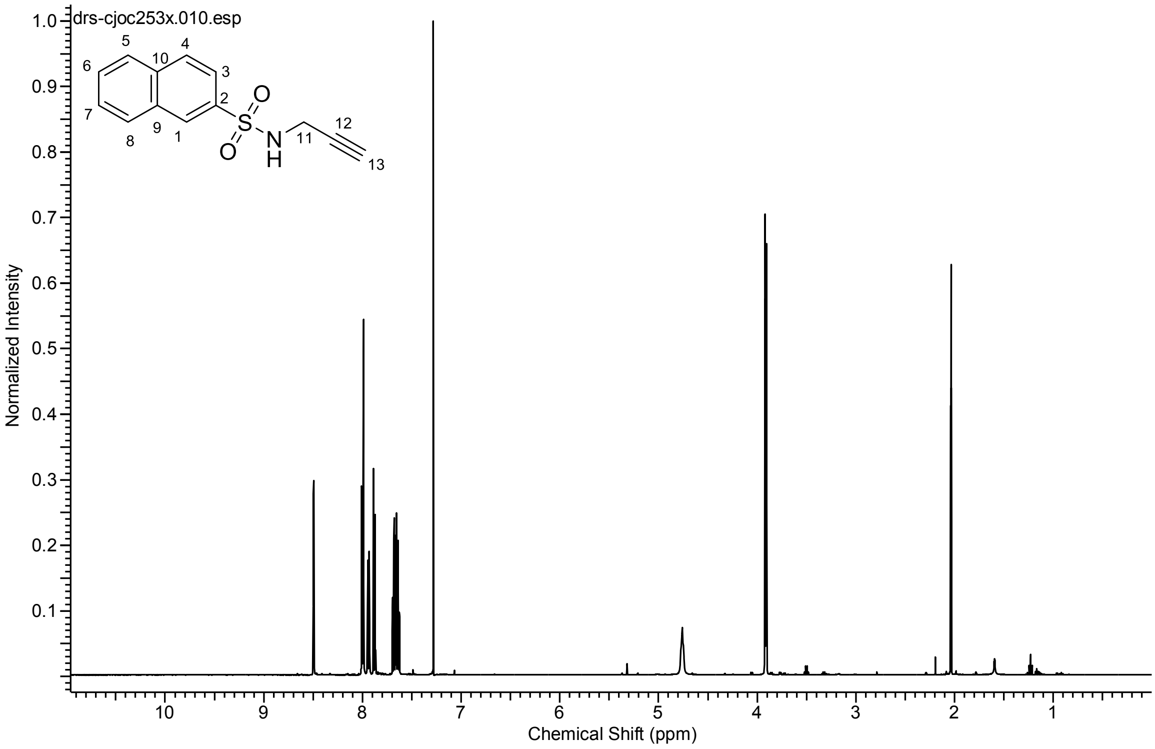
drs-cjoc253x.010.esp
Normalized Intensity
Chemical Shift (ppm)

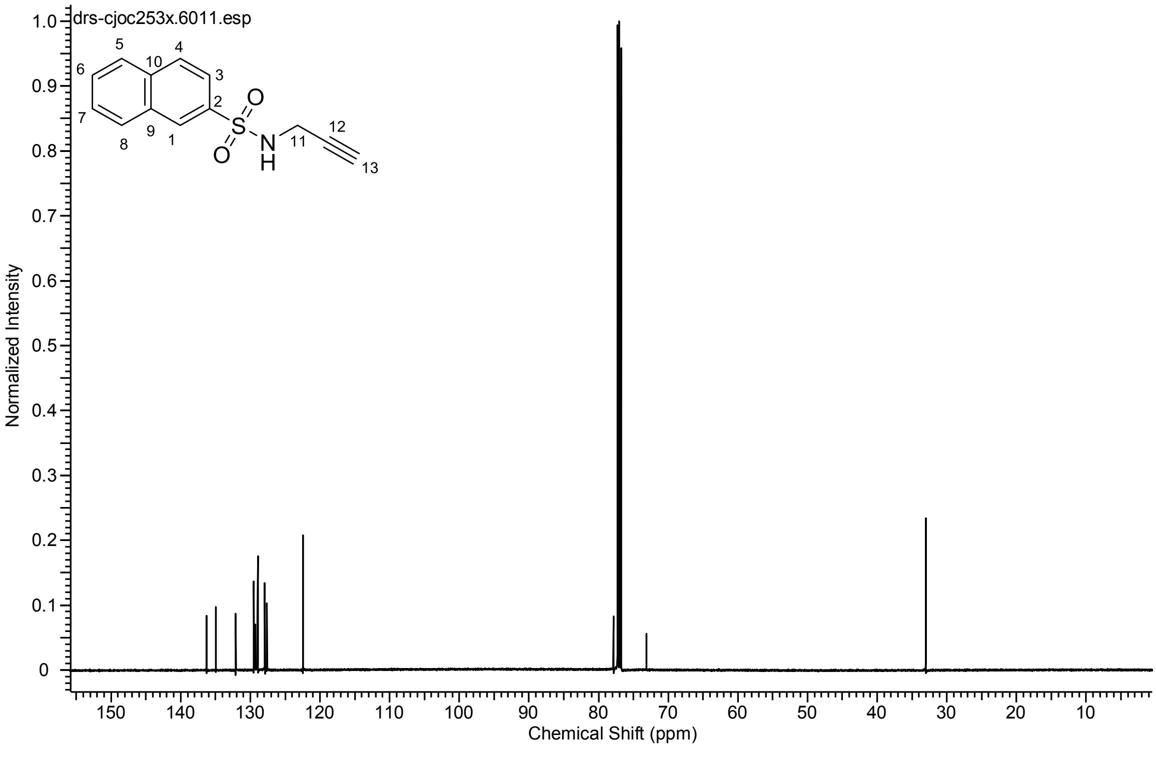
drs-cjoc253x.6011.esp
Normalized Intensity
Chemical Shift (ppm)

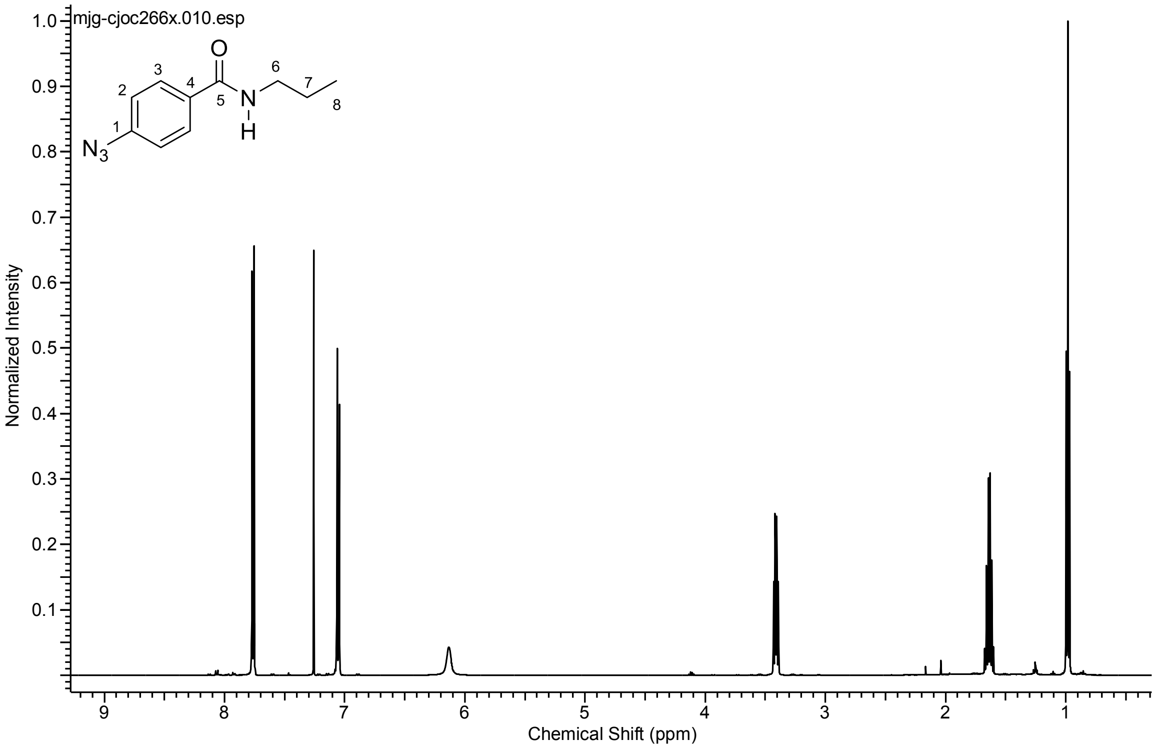
mjg-cjoc266x.010.esp
Normalized Intensity
Chemical Shift (ppm)
N3
O
N
H
1
2
3
4
5
6
7
8

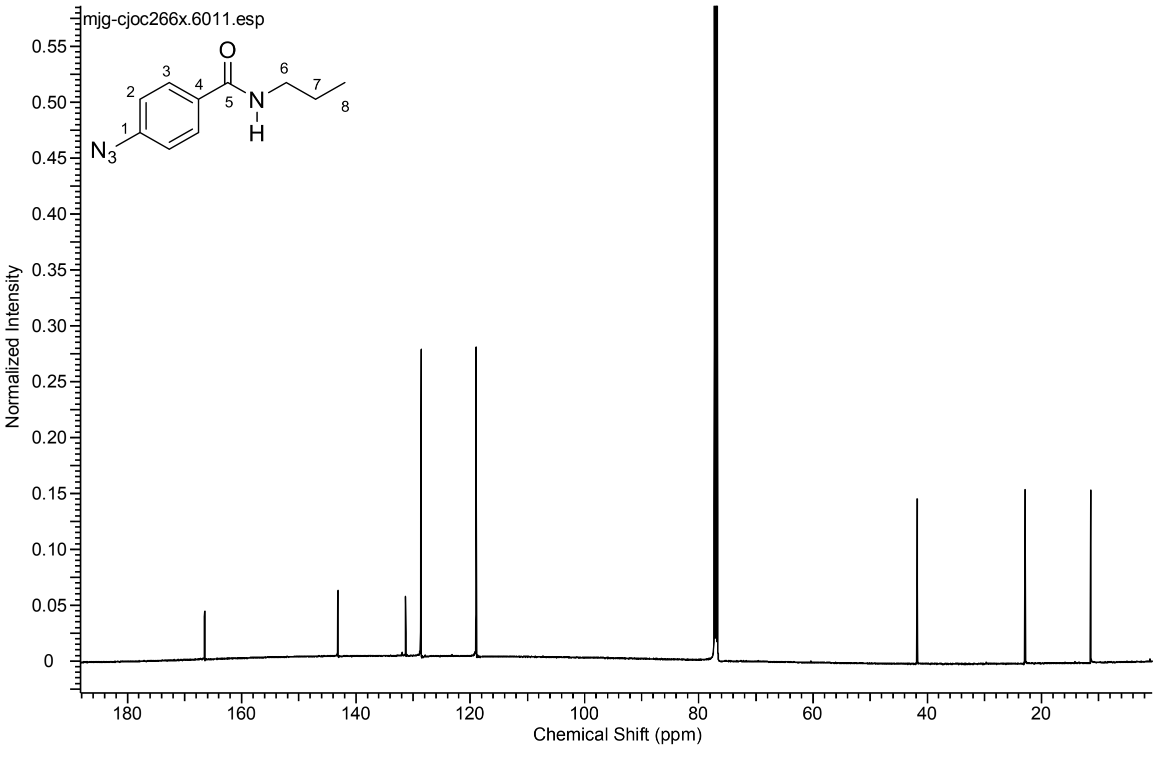
mjg-cjoc266x.6011.esp
Normalized Intensity
Chemical Shift (ppm)
N3
O
N
H
1
2
3
4
5
6
7
8

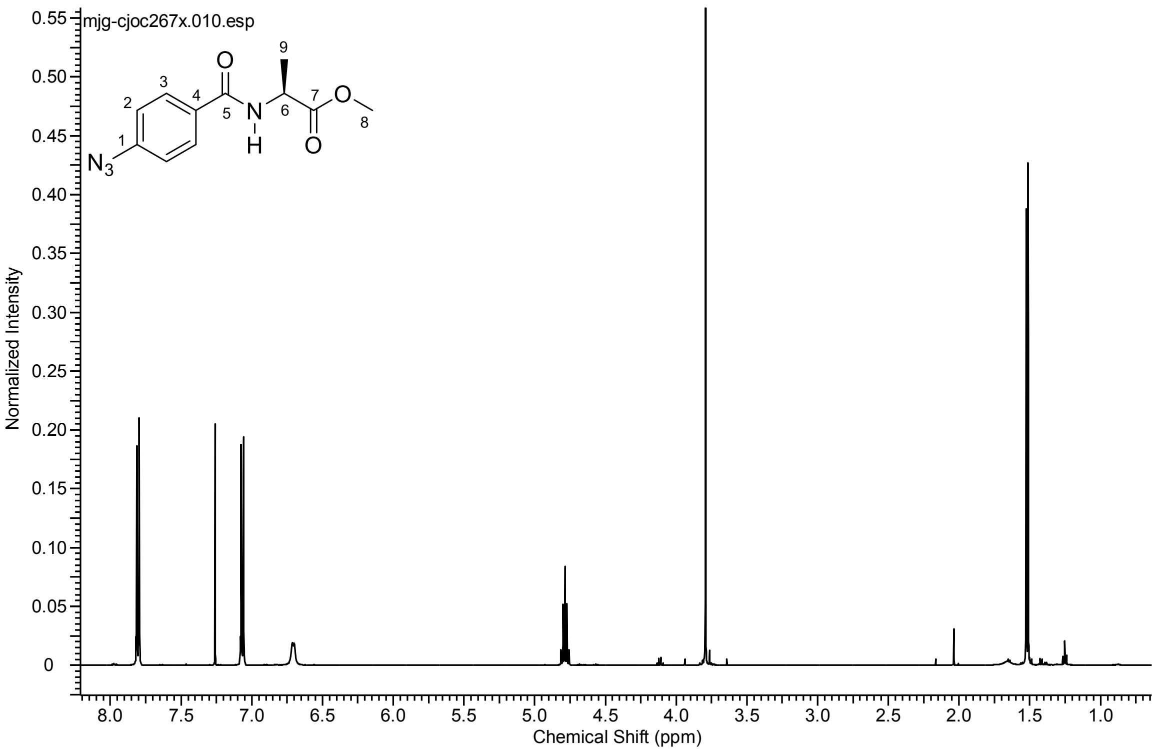
mjg-cjoc267x.010.esp
Normalized Intensity
Chemical Shift (ppm)
N3
O
N
H
O
O
1
2
3
4
5
6
7
8
9

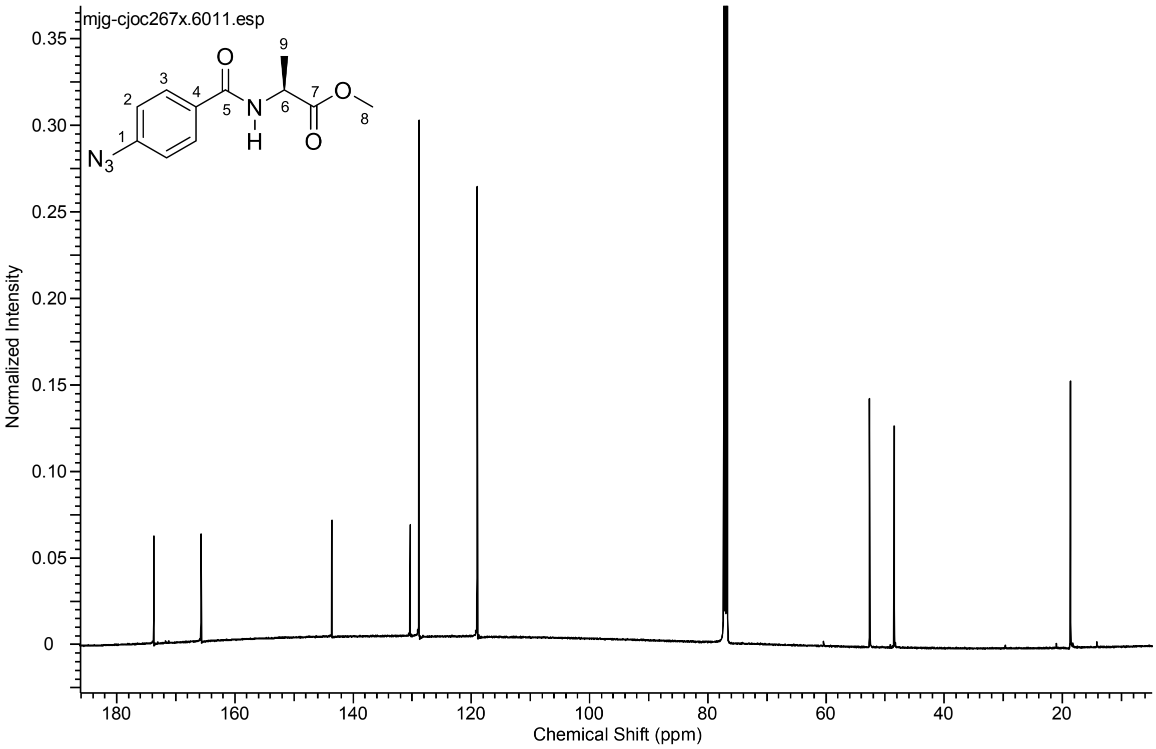
mjg-cjoc267x.6011.esp
Normalized Intensity
Chemical Shift (ppm)
N3
O
N
H
O
O
1
2
3
4
5
6
7
8
9

4

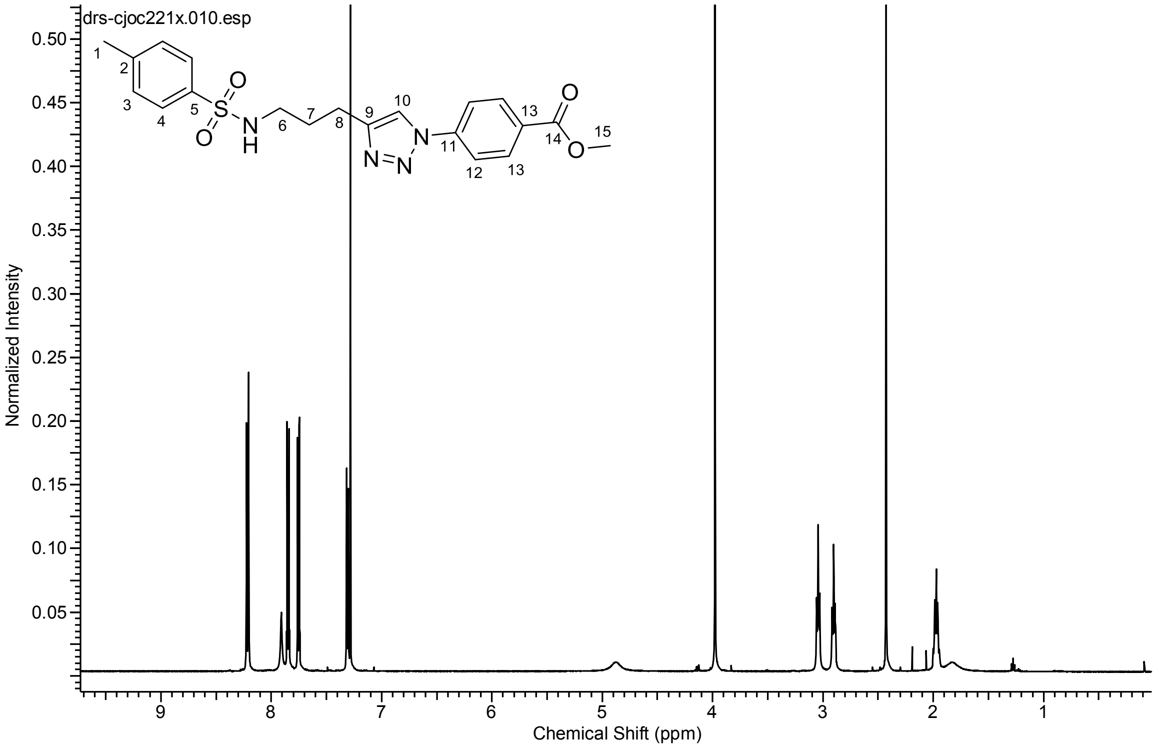
drs-cjoc221x.010.esp
Normalized Intensity
Chemical Shift (ppm)
0.50
0.45
0.40
0.35
0.30
0.25
0.20
0.15
0.10
0.05
9
8
7
6
5
4
3
2
1
O
S
N
H
N
N
N
O
O
1
2
3
4
5
6
7
8
9
10
11
12
13
14
15

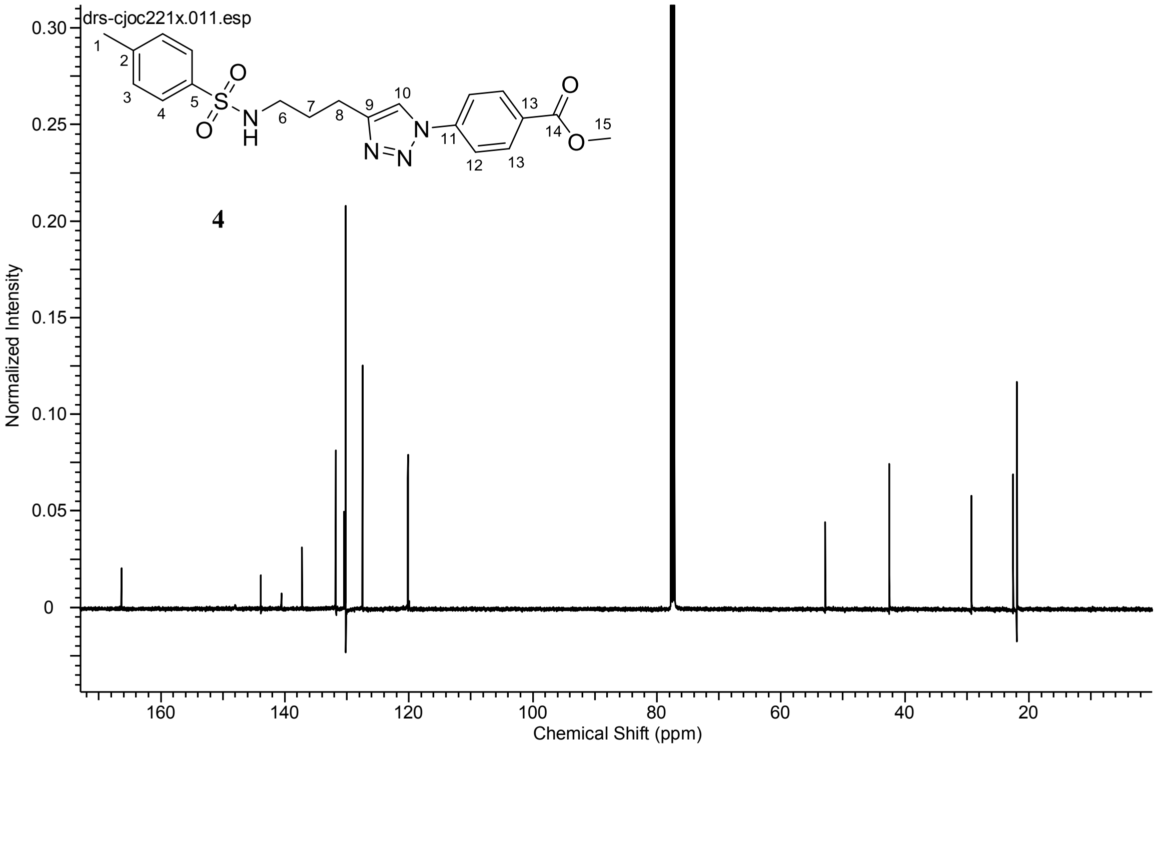
drs-cjoc221x.011.esp
Normalized Intensity
Chemical Shift (ppm)
0.30
0.25
0.20
0.15
0.10
0.05
0
160
140
120
100
80
60
40
20
4

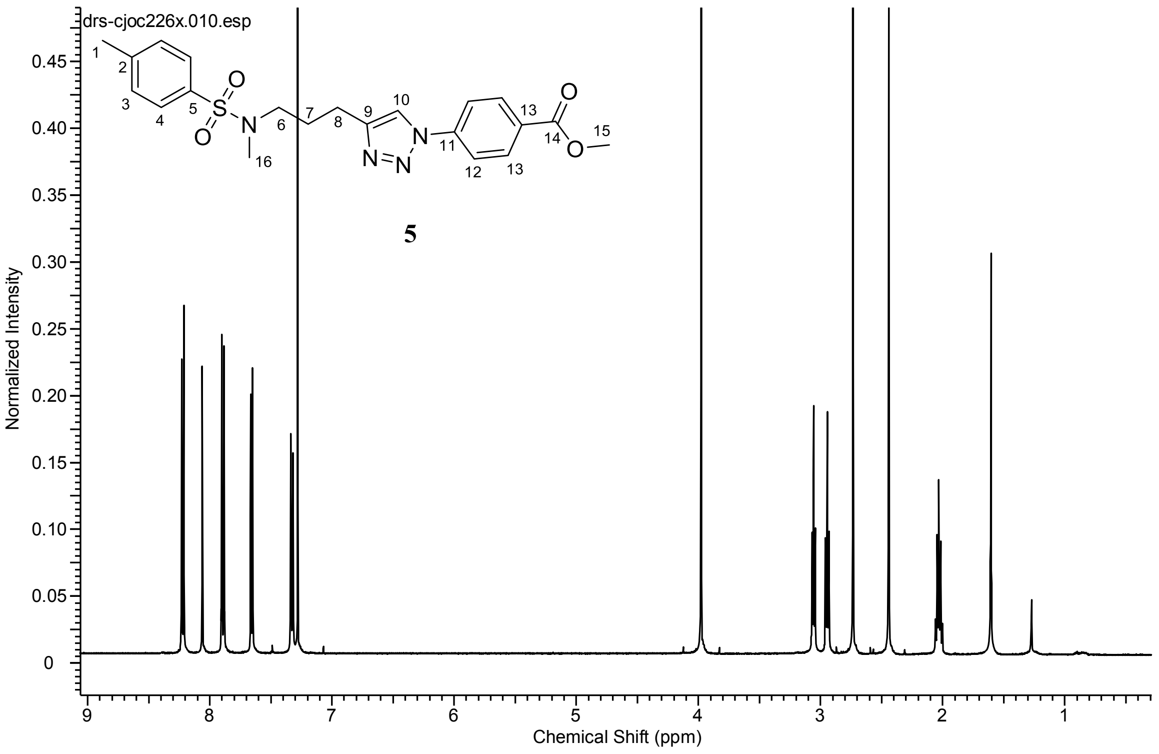
drs-cjoc226x.010.esp
Normalized Intensity
Chemical Shift (ppm)
5
1
2
3
4
5
6
7
8
9
10
11
12
13
14
15
16

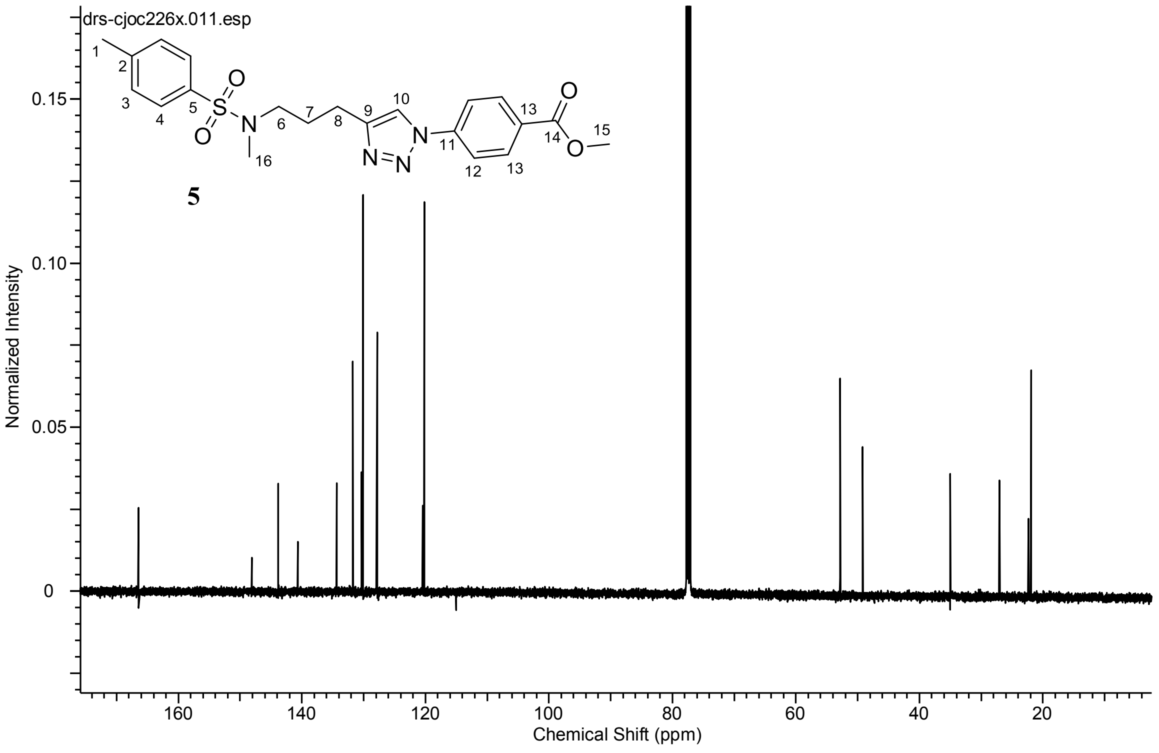
drs-cjoc226x.011.esp
Normalized Intensity
Chemical Shift (ppm)
5

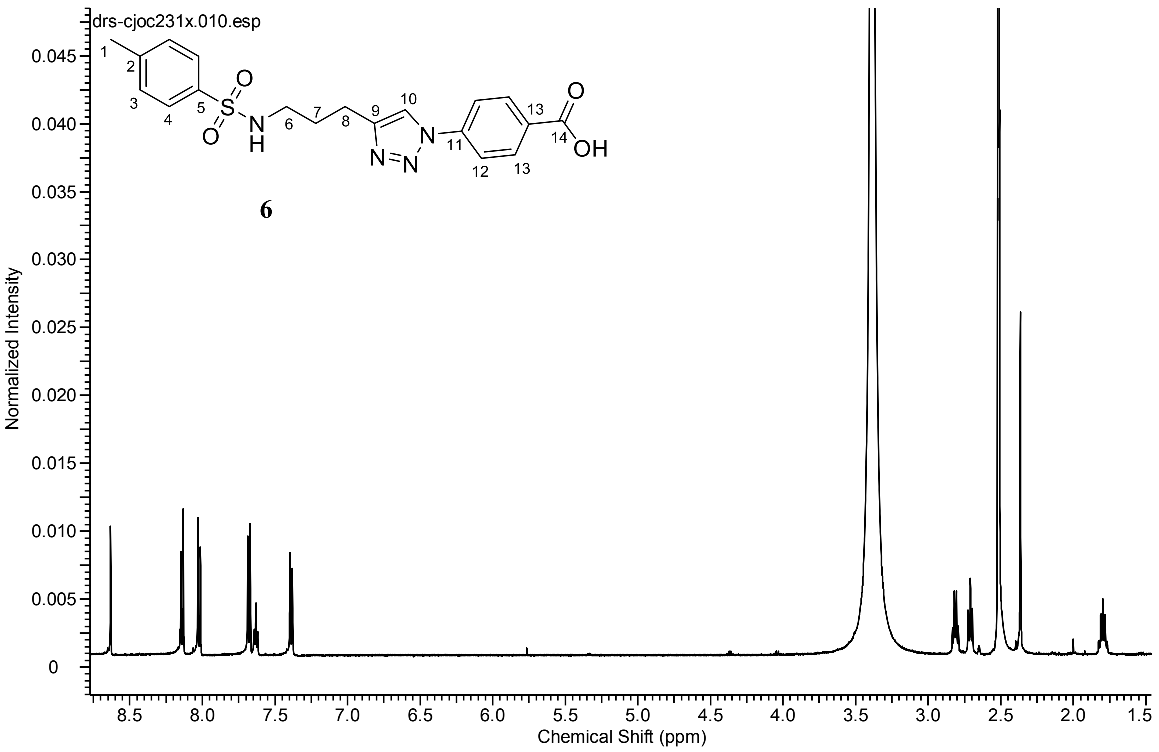
drs-cjoc231x.010.esp
6
Normalized Intensity
Chemical Shift (ppm)

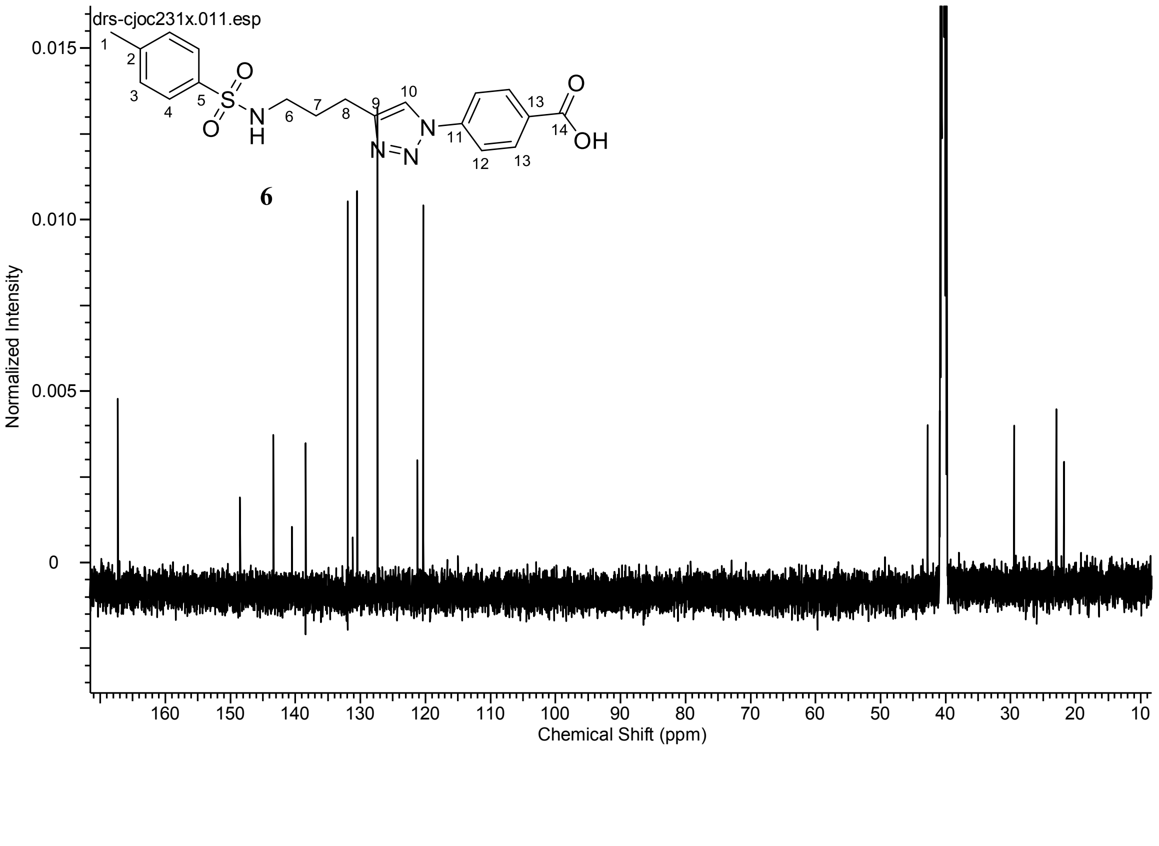
drs-cjoc231x.011.esp
6
Normalized Intensity
Chemical Shift (ppm)

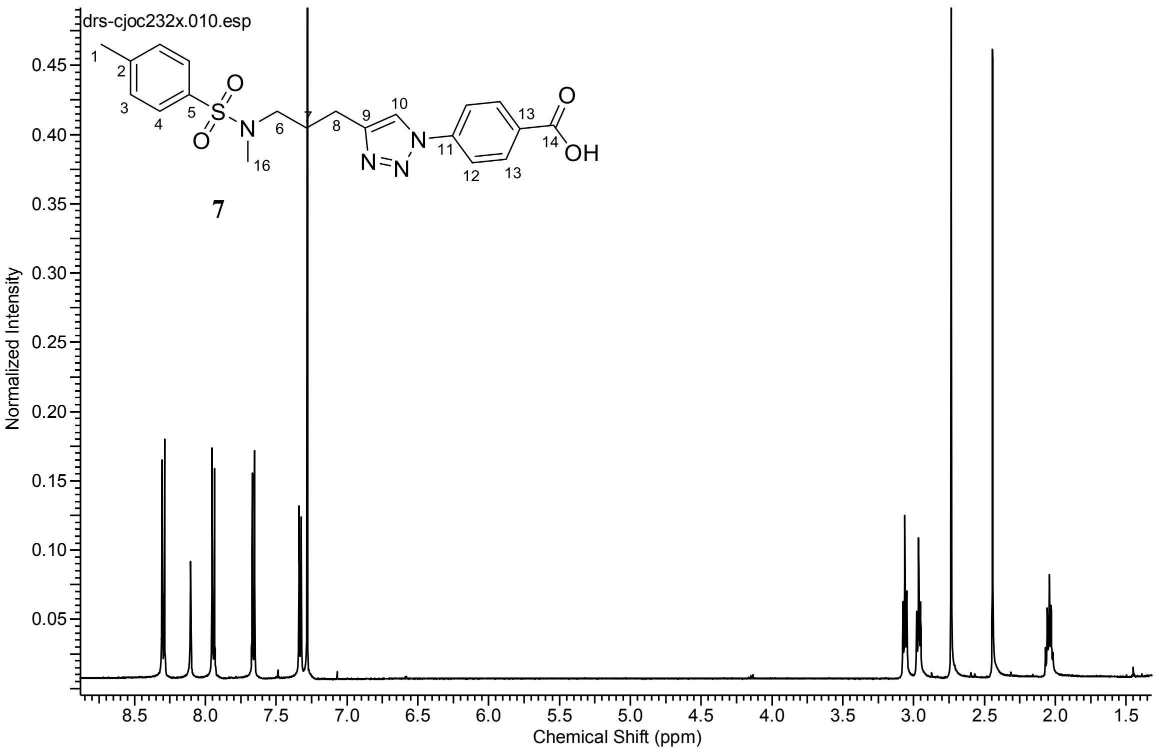
drs-cjoc232x.010.esp
Normalized Intensity
Chemical Shift (ppm)
7

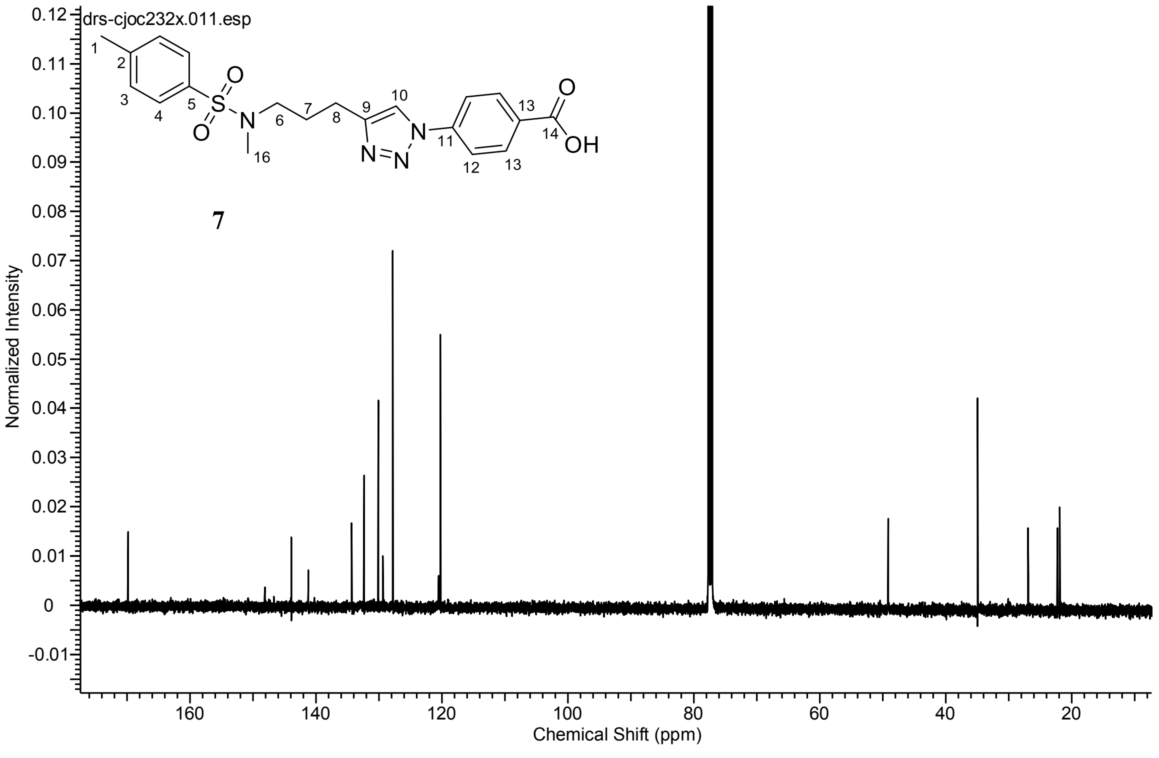
drs-cjoc232x.011.esp
Normalized Intensity
Chemical Shift (ppm)
7

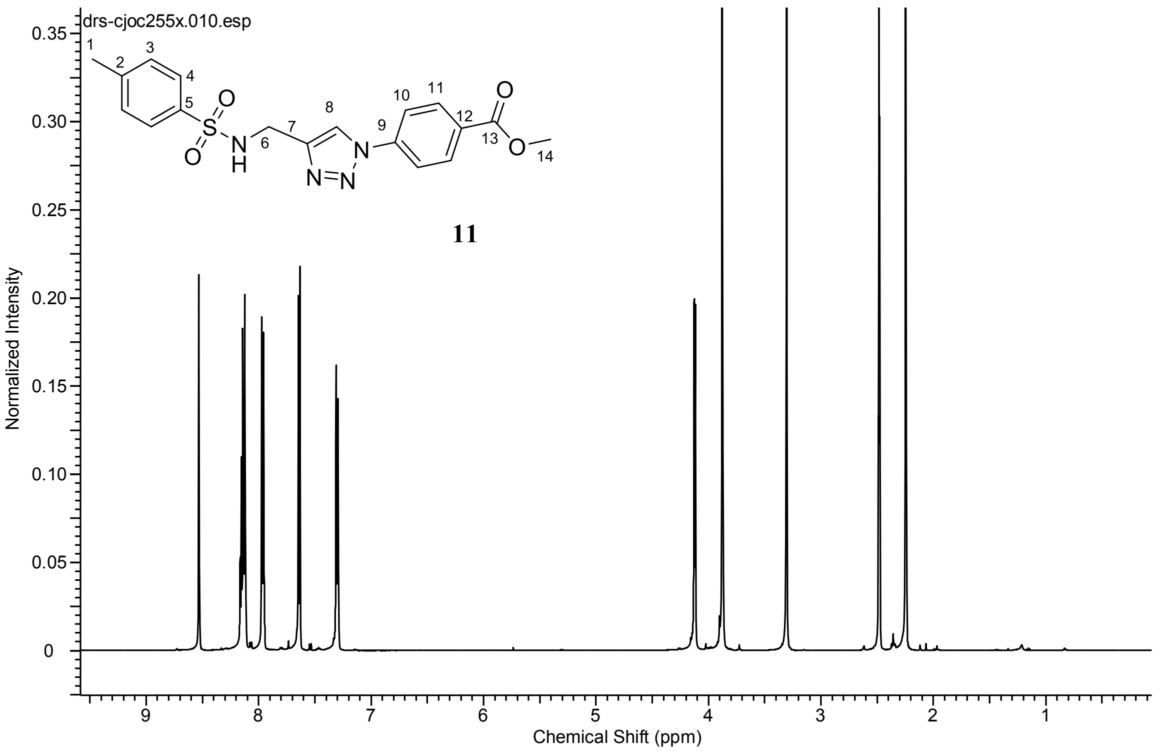
drs-cjoc255x.010.esp
11
Normalized Intensity
Chemical Shift (ppm)

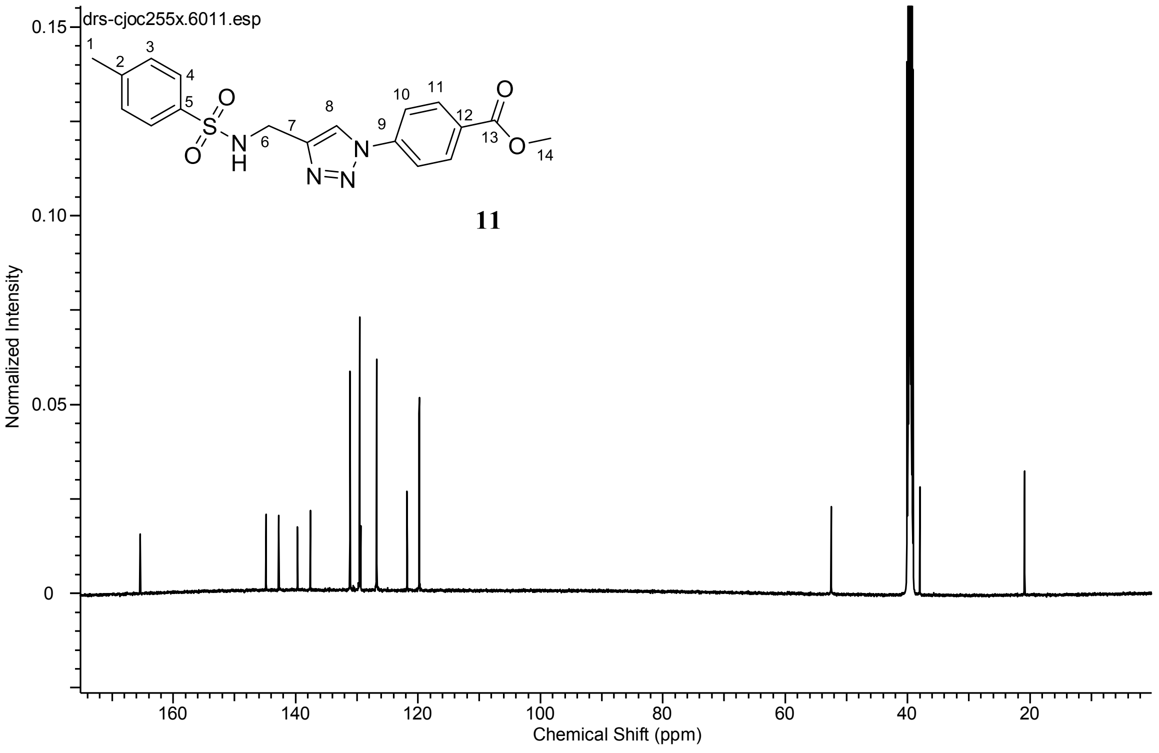
drs-cjoc255x.6011.esp
11
Normalized Intensity
Chemical Shift (ppm)

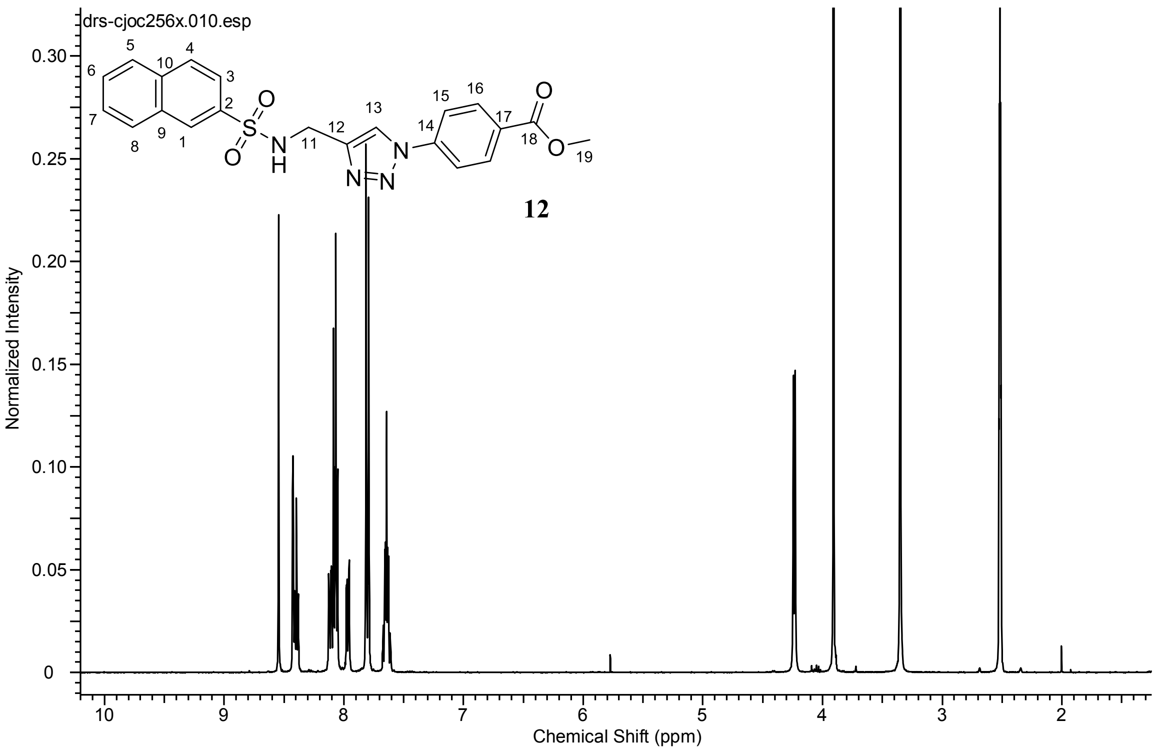
drs-cjoc256x.010.esp
12
Normalized Intensity
Chemical Shift (ppm)

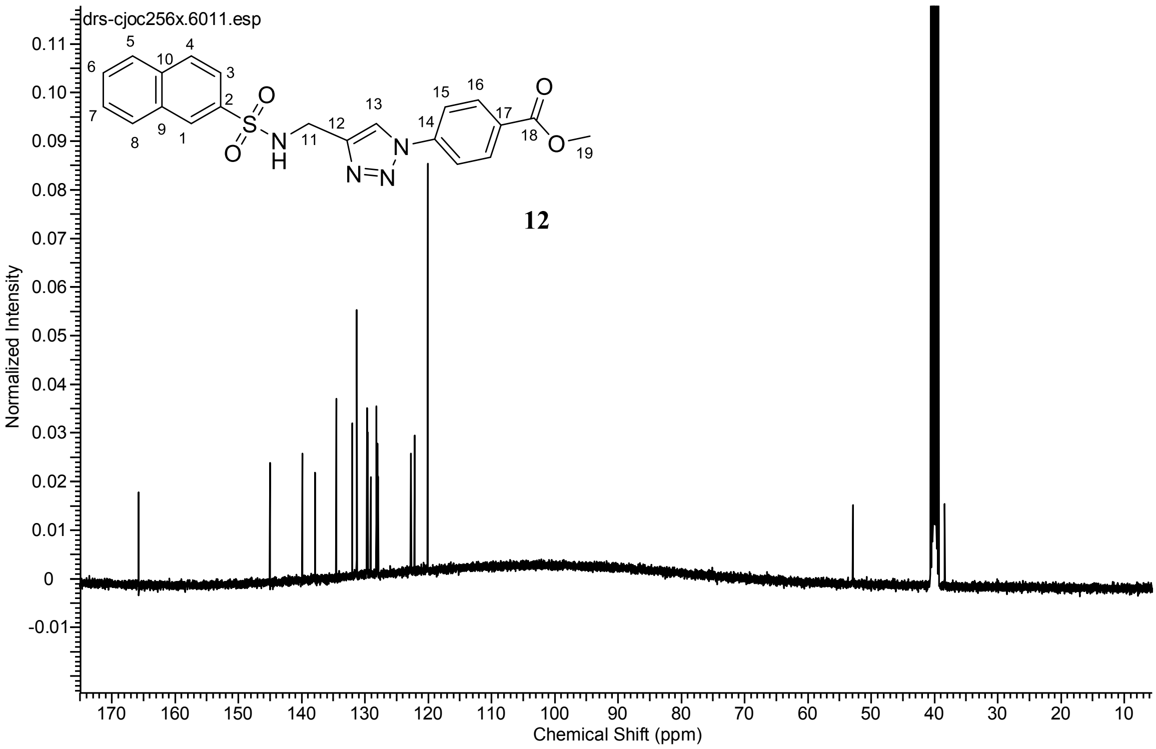
drs-cjoc256x.6011.esp
12
Normalized Intensity
Chemical Shift (ppm)

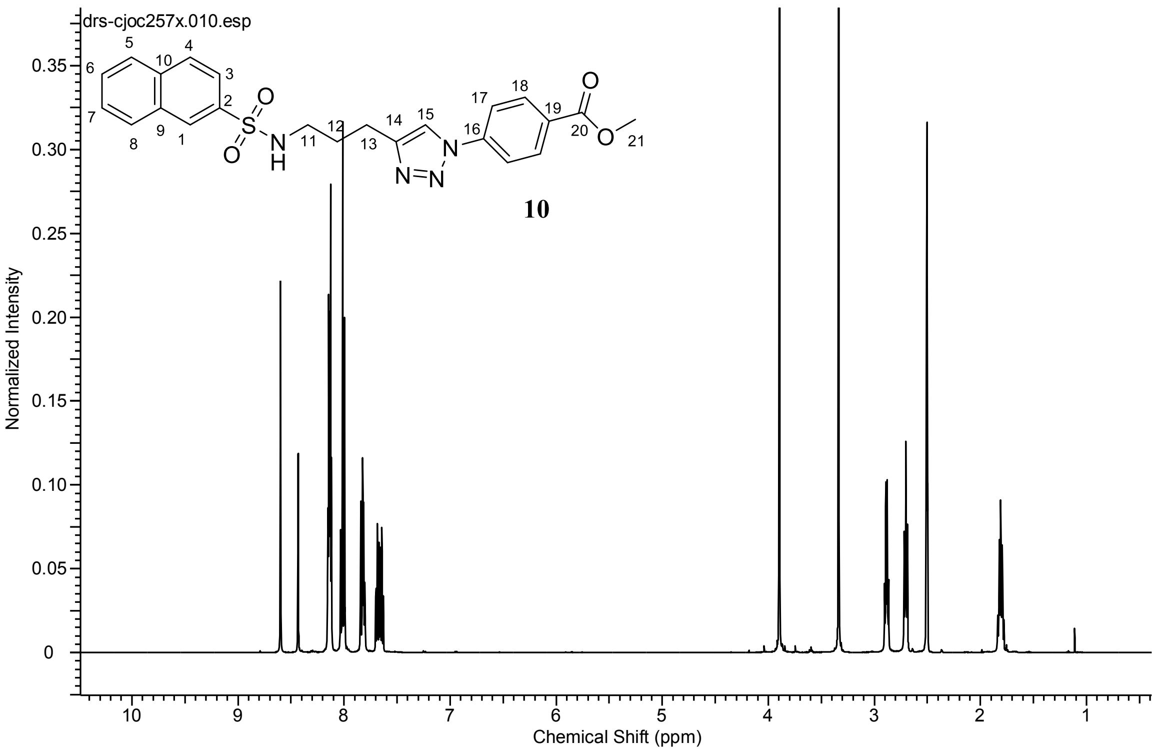
drs-cjoc257x.010.esp
10
Normalized Intensity
Chemical Shift (ppm)

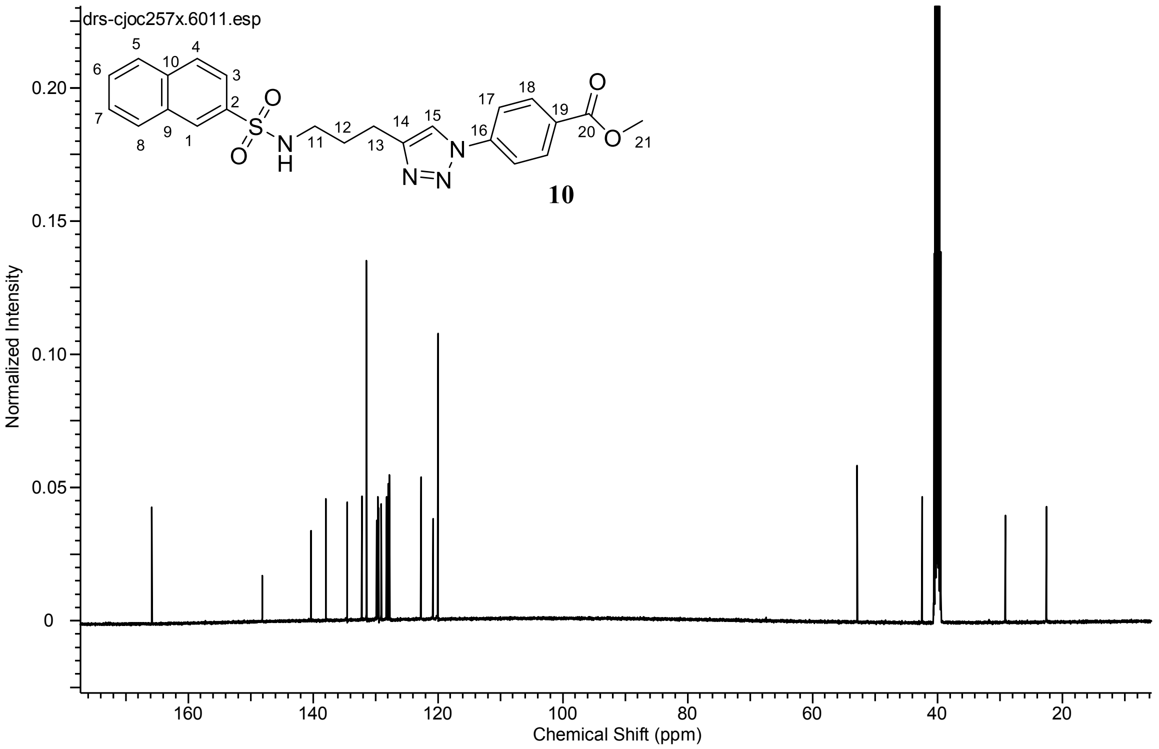
drs-cjoc257x.6011.esp
10
Normalized Intensity
Chemical Shift (ppm)

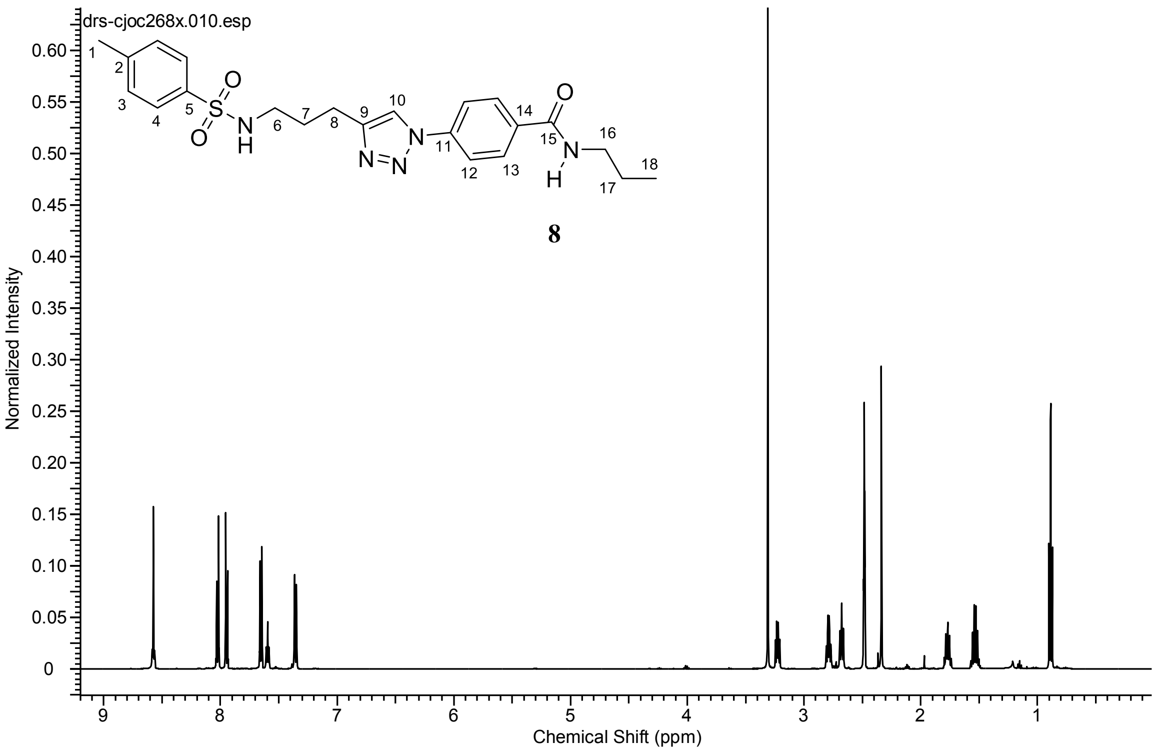

drs-cjoc268x.010.esp
8
Normalized Intensity
Chemical Shift (ppm)

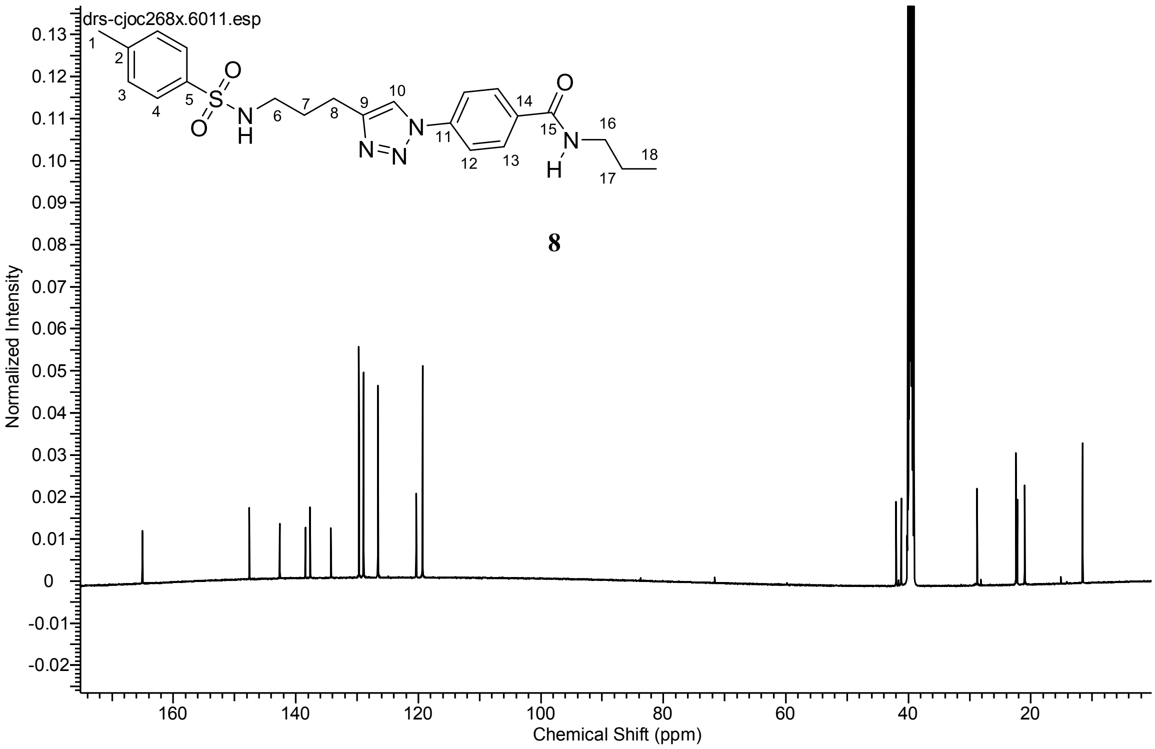

drs-cjoc268x.6011.esp
8
Normalized Intensity
Chemical Shift (ppm)

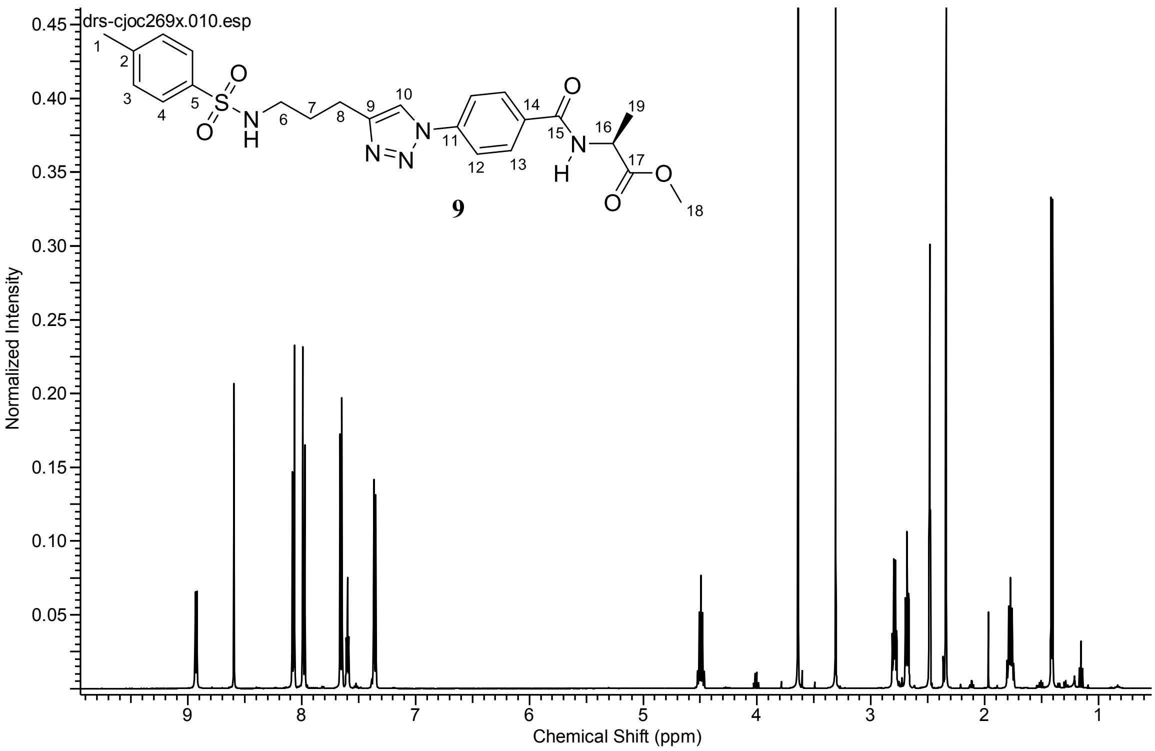
drs-cjoc269x.010.esp
9
Normalized Intensity
Chemical Shift (ppm)

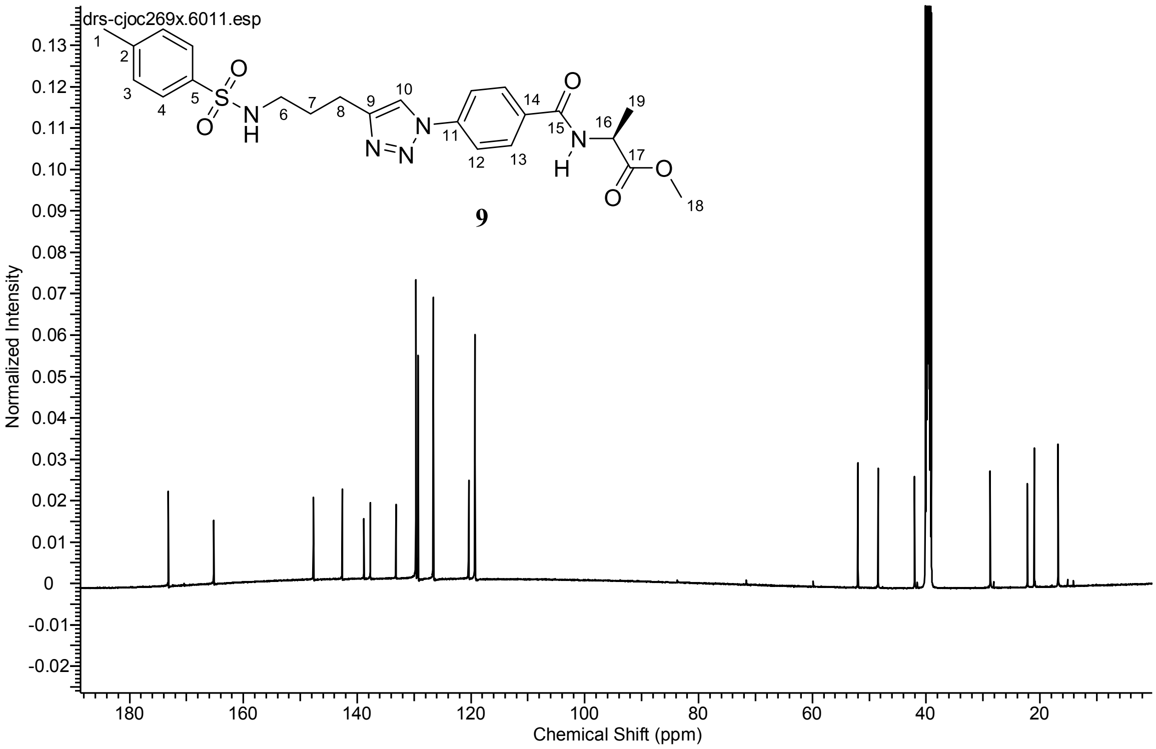
drs-cjoc269x.6011.esp
9
Normalized Intensity
Chemical Shift (ppm)
